# Allosteric drug transport mechanism of multidrug transporter AcrB

**DOI:** 10.1038/s41467-021-24151-3

**Published:** 2021-06-29

**Authors:** Heng-Keat Tam, Wuen Ee Foong, Christine Oswald, Andrea Herrmann, Hui Zeng, Klaas M. Pos

**Affiliations:** 1 Institute of Biochemistry, Goethe-University Frankfurt, Frankfurt am Main, Germany; 2 Present Address: Sosei Heptares, Steinmetz Building, Granta Park, Great Abington, Cambridge, UK; 3 Present Address: Hengyang Medical College, University of South China, Hengyang, Hunan Province China

**Keywords:** Enzyme mechanisms, Antimicrobial resistance, Bacterial structural biology, X-ray crystallography

## Abstract

Gram-negative bacteria maintain an intrinsic resistance mechanism against entry of noxious compounds by utilizing highly efficient efflux pumps. The *E. coli* AcrAB-TolC drug efflux pump contains the inner membrane H^+^/drug antiporter AcrB comprising three functionally interdependent protomers, cycling consecutively through the loose (L), tight (T) and open (O) state during cooperative catalysis. Here, we present 13 X-ray structures of AcrB in intermediate states of the transport cycle. Structure-based mutational analysis combined with drug susceptibility assays indicate that drugs are guided through dedicated transport channels toward the drug binding pockets. A co-structure obtained in the combined presence of erythromycin, linezolid, oxacillin and fusidic acid shows binding of fusidic acid deeply inside the T protomer transmembrane domain. Thiol cross-link substrate protection assays indicate that this transmembrane domain-binding site can also accommodate oxacillin or novobiocin but not erythromycin or linezolid. AcrB-mediated drug transport is suggested to be allosterically modulated in presence of multiple drugs.

## Abstract (web summary)

Gram-negative bacteria can display intrinsic antibiotic resistance due to the action of tripartite efflux pumps, which include a H^+^/drug antiporter component. Here, the authors present a structure-function analysis of antiporter AcrB in intermediate states of the transport cycle, showing novel drug-binding sites and transport pathways.

## Introduction

**Fig. 1:**
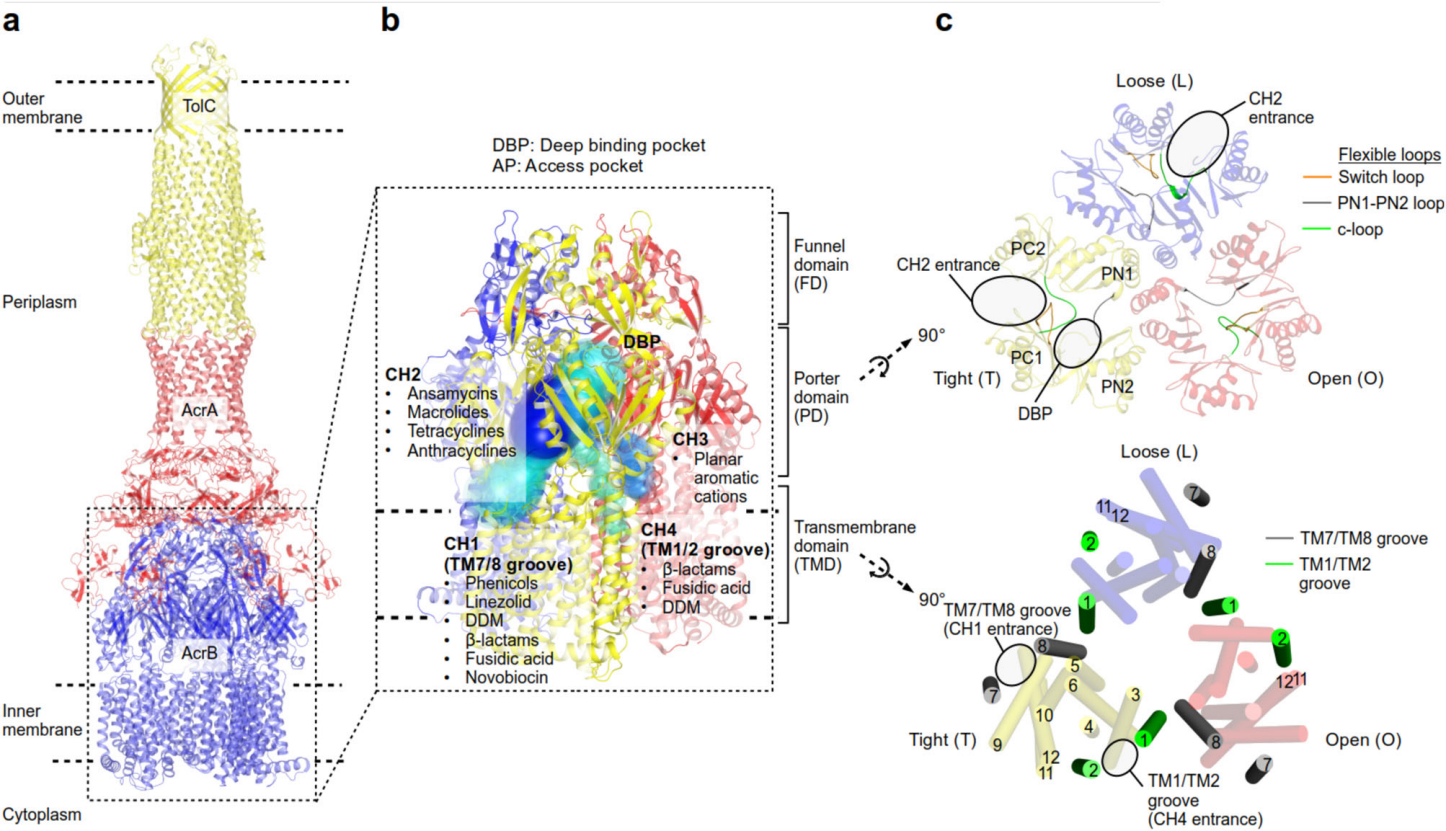
Visualization of the AcrB channel CH1–CH4. **a** AcrAB-TolC tripartite complex. **b** Putative AcrB drug transport pathways (CH1–4) and substrate preferences of each channels. **c** Top view from the periplasm on the AcrB porter (upper panel) and transmembrane domain (lower panel).

Gram-negative bacteria comprise a double-layered envelope, the inner and outer membrane (IM and OM) enclosing a compartment known as the periplasm. These bacteria are intrinsically resistant against cytotoxic substances due to the synergistic action of the IM and OM and a network of multidrug efflux systems^1^. Various efflux pumps with broad overlapping drug preferences sequester and transport drugs across the IM to supply the highly polyspecific tripartite resistance–nodulation–cell division (RND) efflux pump systems with compounds to be extruded across the OM barrier^2,3^. In clinical Gram-negative strains, RND efflux pumps are often upregulated and contribute to the overall multidrug resistance phenotype, leaving infections untreatable with our current arsenal of antibiotics^4^. AcrAB-TolC is the main drug efflux system in *Escherichia coli* (Fig. 1a)^2,5^. AcrB is the homotrimeric IM proton-motive force-driven H^+^/drug antiporter component (Supplementary Note 1.1) which recognizes and actively transports multiple drugs (antibiotics, detergents, dyes, and solvents) via the tightly connected AcrA and TolC channels across the OM, leading to an observable resistance phenotype (Fig. 1a)^6–17^. Four different entry channels (CH1–CH4) have been described with putative entrances from the periplasm or the outer leaflet of the IM toward the two drug-binding pockets, the access pocket (AP) and deep binding pocket (DBP) inside the AcrB pump (Fig. 1b, c)^8,9,17–19^. For one of these observed channels, CH3, specificity towards planar aromatic cationic drugs has been postulated on basis of mutational analysis of the CH3 entrance region and by blocking the path between the AP and DBP^18^. The drug specificity of the other channels is elusive, but binding of erythromycin, rifampicin^10^, and a doxorubicin-dimer^12^ to the AP indicates that CH2 prefers high molecular weight (HMW) drugs, and CH4 was postulated to transport carboxylated drugs^19^ (Fig. 1). The transport of drugs through the channels has been postulated to be mediated by a mechanism resembling the action of a peristaltic pump^8^. Moreover, the efflux of certain drugs was shown to be enhanced in the simultaneous presence of other substrates, indicative of a kinetically cooperative transport mechanism^20^.

In this work, we address three main questions: (1) How are drugs transported through the channels (CH1–CH4) towards the drug-binding pockets (AP and DBP) and is there a drug specificity for each channel? (2) Is the transport mechanism different for high molecular weight (HMW) drugs like macrolides and ansamycins compared to low molecular weight (LMW) compounds like β-lactams, detergents and dyes? And (3) what are molecular determinants of the observed cooperativity in the presence of more than one drug?^20^ We present co-structures of AcrB in different intermediate transport states on basis of which we conducted extensive mutational analysis combined with drug susceptibility and thiol cross-linking studies. The results suggest that drugs are transported via dedicated transport pathways through AcrB depending on their physicochemical properties, which results in a broad polyspecific drug resistance phenotype by the action of a single pump. We also found that AcrB comprises a deep transmembrane binding pocket for fusidic acid, oxacillin, or novobiocin. We suggest that it might act as an allosteric binding site, facilitating binding of other drugs to other sites and enhancing the catalytic efficiency of drug efflux.

## Results and discussion

### Substrate access from the membrane towards the deep binding pocket (DBP)

**Fig. 2:**
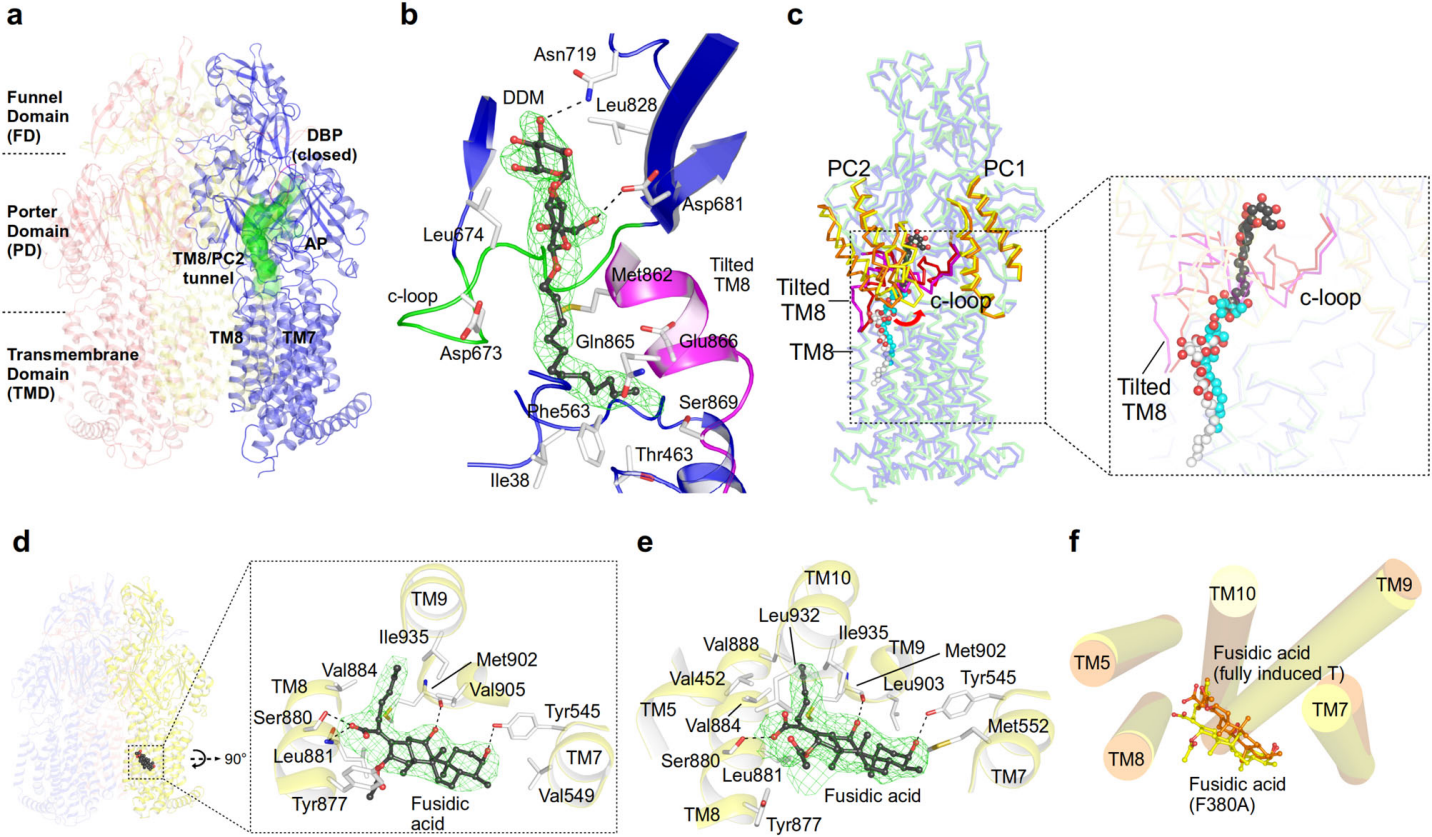
Transport of LMW/LPSA drugs by CH1 via the TM7/TM8 groove. **a** Side-view of the AcrB trimer with the TM8/PC2 tunnel of the L2 protomer (blue) starting from the TM7/TM8 groove to the access pocket (AP), and ending at the entrance of the (closed) deep binding pocket (DBP) underneath the switch loop (magenta). **b** DDM (carbon, black; oxygen, red) binding to the TM8/PC2 tunnel with the tilted TM8 (magenta) and c-loop (green). The Polder electron density map (green mesh) assigned to DDM is contoured at 4.0 σ. **c** Conformational changes of tilted TM8/c-loop (L protomer, magenta; L2: red) and PC1/PC2 subdomains (L, orange; L2: yellow). Inset: DDM binding to the L protomer (white), L2 protomer (black), and DDM bound to an AcrB/erythromycin complex (PDB ID: 4ZJL^16^) (cyan). **d** Binding of fusidic acid (FUA) to the TM7/TM8 groove of AcrB-Phe380Ala. Inset: Polder electron density map (green mesh) assigned to FUA (carbon, black; oxygen, red) is contoured at 4.2 σ. **e** Binding of FUA to the TM7/TM8 groove in the T^FI-LIG2^ (fully induced) protomer (carbon, black; oxygen, red). Polder map (green mesh) of FUA is contoured at 5.0 σ. **f** Superimposition of the TMD (TM5, TM7-10) of FUA bound to AcrB-Phe380Ala (yellow) and the AcrB T^FI-LIG2^ (orange) T protomers.

The transmembrane domains (TMDs) of the AcrB L and T protomers comprise two grooves (TM1/TM2 and TM7/TM8) which serve as putative entrance sites to channel CH1 and CH4, leading substrates to the access pocket (AP) or deep binding pocket (DBP) inside the porter domain (PD)^9,10,12,18,19,21^ (Fig. 1). In several crystal structures, the AcrB substrate dodecyl β-D-maltoside (DDM) is apparent at the TM7/TM8 groove entrance site of CH1 of the L protomer^9,12,19,22^ (Fig. 2a–c). Here, we present a crystal structure of the asymmetric LTO AcrB trimer (in complex with designed ankyrin repeat proteins (DARPins)^9,12^ as crystallization chaperones, used for all structures presented) with the L protomer in a L-to-T transition state (L2 protomer, see Supplementary Table 1 for differences between L and L2). A DDM molecule is trapped in a transient L to T state of the CH1 tunnel (which we designated TM8/PC2 tunnel, see Supplementary Note 1.2) towards the AP (Fig. 2a, b). The DDM maltose moiety is situated at the AP, proximal to a small flexible loop which separates the AP and the DBP (switch loop, Fig. 1c)^12^ and the closed DBP. DDM is sandwiched between Leu674 and Leu828 and forms hydrogen bonds with Asn719 and Asp681 (Fig. 2b). The DDM acyl chain localizes at the interface of the PC2 subdomain (in the PD, Fig. 1c) and the TM7/TM8 groove (in the TMD, Fig. 1c) and has several van der Waals interactions with non-polar side chains of the tilted TM8 and with a flexible loop which connects the PC1 and PC2 subdomains (the c-loop, Fig. 1c and Fig. 2b, c). A most prominent difference is the large conformational change of the c-loop, moving from a TM8 proximal orientation to a distal one (Fig. 2c). This intermediate conformation is in support of the suggested peristaltic pump hypothesis^8^, rather than by diffusion of drugs through the observed tunnels towards the AP and/or DBP. A comparison with two other structure snapshots^12,22^ featuring DDM in the TM7/TM8 groove a rather clear picture emerges of the peristaltic movement of DDM toward the observed intermediate binding site (Fig. 2c). Residues lining the transient CH1 pathway were systematically substituted by Ala and the substitution-variants analyzed via drug susceptibility tests with 14 different drugs (Supplementary Data 1a, b; Supplementary Fig. 7a, b; Supplementary Note 1.3). Substitution of residues facing the inside of the TM7/TM8 groove and those residing in the entrance area of CH1, i.e. Ile38^23^, Ile466, Leu393, Phe563, Ile671, or Leu674 (the latter also in combination with substitutions in juxtaposed Asp681, Asn719, and Met862) show a most prominent effect on susceptibility for drugs with low molecular weight (LMW) and low polar surface area (LPSA), such as β-lactams, linezolid, and phenicols. Susceptibility tests furthermore implicate that the transient state of CH1 is specific for DDM (Supplementary Fig. 1b) as simultaneous Ala-substitution of both DDM-interaction partners, Phe563 and Leu674 (Fig. 2b), results in complete sensitivity towards DDM (with the Asp407Asn substitution variant as negative control^24^). Sensitivity towards polyaromatic cationic drugs or to a lesser extent also erythromycin, on the other hand, was unchanged to all 40 out of 42 substitution variants compared to cells harbouring wild-type AcrB (Supplementary Data 1b; Supplementary Fig 7b), indicating that polyaromatic cationic drugs and erythromycin take other paths through AcrB (through CH2 and CH3, Fig. 1b).

**Fig. 3:**
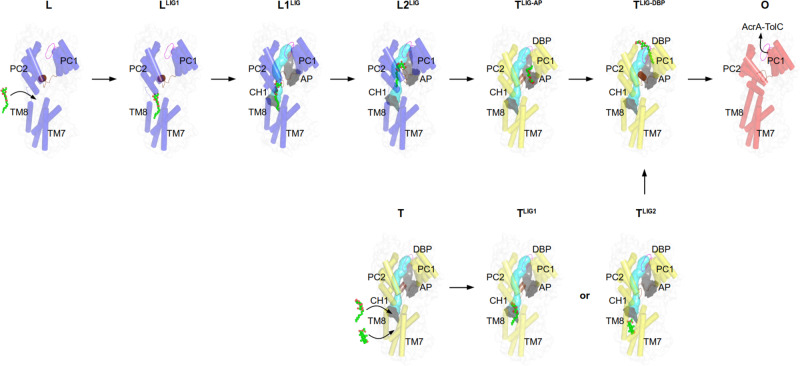
Proposed drug transport mechanism via CH1 and the TM8/PC2 pathway during L, T, and O functional rotation based on crystal structures. Dodecyl β-D-maltoside (DDM) and fusidic acid (FUA) are depicted as spheres (carbon, green; oxygen, red). The switch loop (magenta) and c-loop (brown) conformations are indicated in every protomer (AP access pocket, DBP deep binding pocket, CH1 channel 1). For further discussion on the mechanism see main text and Supplementary Note 1.7. PDB IDs of the displayed protomer structures: L, L^LIG1^, T, T^LIG1^, T^LIG-AP^, O: 4DX5; L1^LIG^: 4ZJL; L2^LIG^: 6ZOA; T^LIG2^: 6ZOF; T^LIG-DBP^: 3W9I.

The Phe563 side chain at the entrance to CH1 appears to act as a guarding residue for further CH1 entry^21^ (Supplementary Fig. 1a, d, e; Supplementary Note 1.4). Structural analysis of trimeric AcrB-Phe563Ala reveals a rather unexpected TTT conformation (Supplementary Fig. 1c; Supplementary Table 1a) and the c-loop with Leu674 appears to block the tunnel (Supplementary Fig. 1d). Indeed, cells harbouring Phe563Ala are growth-compromised in presence of phenicols, β-lactams, fusidic acid (FUA), and novobiocin (Supplementary Data 1b; Supplementary Fig. 7b; Supplementary Note 1.5). Since β-lactams and FUA have been shown to be transported via CH4 as well^19,25^, we realized that the c-loop may play an important role in drug transport not only via CH1 but also via CH4 (Fig. 2c; Supplementary Fig. 1e; Supplementary Note 1.6). We therefore compromised the entrances to both CH1 and CH4 (Phe380Ala_Phe563Ala), and observed indeed a strong sensitivity towards FUA, β-lactams, and the failure to grow on DDM by cells harbouring this variant (Supplementary Data 1c; Supplementary Fig. 1b; Supplementary Fig. 7c), whereas wild-type activity is retained towards doxorubicin and rhodamine 6 G (R6G), presumably transported by the unaffected CH3^18^ (Fig. 1b; Supplementary Data 1c; Supplementary Fig. 7c). CH4 transport-compromised AcrB-Phe380Ala shows binding of FUA at the lower end of the TM7/TM8 groove proximal to the cytoplasmic side (Fig. 2d; Supplementary Fig. 1f), surrounded by TM5 and TM7-TM10. This implies that FUA may enter via CH1, but also via CH4^19^ and is in line with the observed substitution phenotypes (Supplementary Data 1c; Supplementary Fig. 7c). In sum, the results suggest that CH1, with its TM7/TM8 entrance groove, is the main transport pathway for LMW/LPSA drugs, DDM, as well as FUA and novobiocin, starting with binding at the TM7/TM8 groove and peristaltic movement of the drugs to the DBP (Fig. 1b, c and Fig. 3 and Supplementary Data 1; Supplementary Figs. 1a and 7a; Supplementary Note 1.7). β-Lactams, DDM and FUA are transported independently via both CH1 and CH4^19,25^ (Fig. 1b). As planar polyaromatic cations (like doxorubicin and R6G) are transported via CH3^18^, we embarked on investigating the transport of larger (HMW) drugs via CH2.

### Drug binding and transport of HMW drugs and the role of the switch loop

**Fig. 4:**
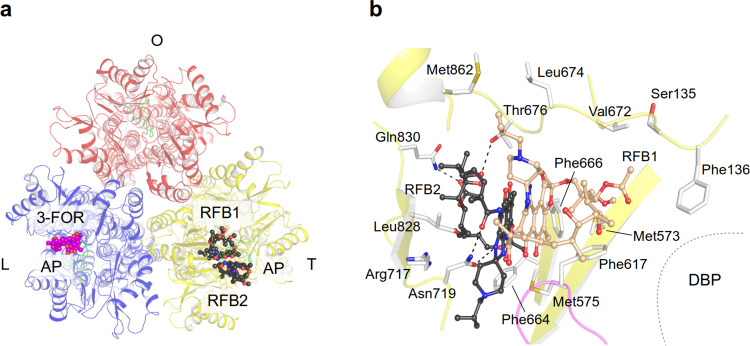
Binding of HMW drugs to the access pocket of the L and T protomers. **a** AcrB porter domain top view with 3-formylrifamycin SV (3-FOR, carbon, magenta; oxygen, red; nitrogen, blue) and rifabutin (RFB1/RFB2, carbon, black) bound to the access pocket (AP) of AcrB adopted from two different AcrB co-crystal structures (PDB ID: 6ZOB, 6ZO9, respectively). Switch loop is colored in green. **b** Binding of two rifabutin (carbon, white (RFB1) or black (RFB2)) to the AP of the T protomer. The switch loop is coloured in magenta (DBP deep binding pocket).

High molecular weight (HMW) drugs such as erythromycin and rifampicin bind in the AP of the L protomer^10^ along the CH2 pathway (Fig. 1b). Our susceptibility tests (Supplementary Data 2a; Supplementary Fig. 7d) and a co-crystal AcrB/3-formylrifamycin SV (3-FOR) structure (Fig. 4a and Supplementary Fig. 2a) show that other ansamycins (rifampicin derivatives) are also substrates for AcrB (Supplementary Note 2.1). The interaction of the drug with the short glycine-rich switch loop mediates the accommodation of 3-FOR inside the AP (Supplementary Fig. 2a). In an attempt to prevent ansamycin binding, we made the switch loop more rigid by Gly619 and/or Gly621 to Pro substitutions. Surprisingly, the crystal structure of these variants showed propensity to bind either 3-FOR to both L protomer AP and the smaller AP in the T protomer in the same AcrB trimer (Fig. 1b, c and Supplementary Fig. 2b–f) or two rifabutin (RFB) molecules to the AP of the T protomer (Fig. 1b, c and Fig. 4a, b and Supplementary Fig. 2g; Supplementary Note 2.2). Both switch loop single-Pro-substitution variants retain full activity for the co-crystallized substrates, 3-FOR (except Gly621Pro: 46%) and RFB (Supplementary Data 2a; Supplementary Fig. 7d) and bind minocycline as well as FUA (Gly619Pro_Gly621Pro) in a congruent manner in the T protomer DBP or TM1/TM2 groove (as shown for wild-type AcrB^12,25^, respectively, (Supplementary Fig. 2h–k). Support for both the observed ansamycin (Supplementary Fig. 2; Supplementary Note 2.3) and erythromycin binding (Supplementary Fig. 2l-m; Supplementary Note 2.4) and transport comes from substitutions of residues at and near the observed binding sites, which resulted in severe (Phe136, Phe615) or strong (Met575, Phe617, Thr676, and Glu826) increase in susceptibility towards ansamycins (Supplementary Data 2a; Supplementary Fig. 7d). Residues Phe136/Phe615/Phe617/Met575 appear to form a gate for HMW drugs from the AP towards the DBP and the exit tunnel, whereas Phe666/Thr676/Glu826 might be involved in the CH2 entry toward the AP.

**Fig. 5:**
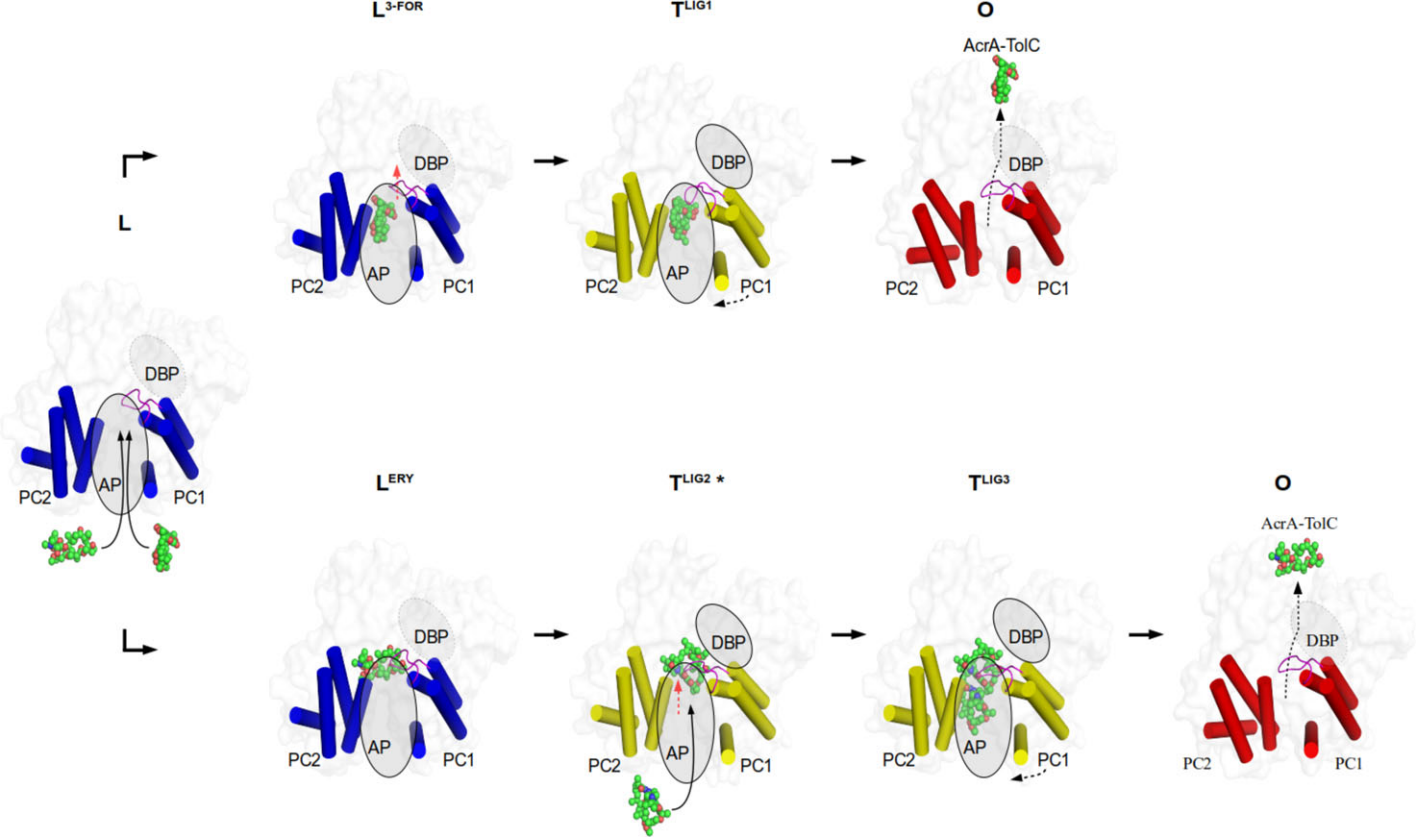
Proposed transport mechanism for HMW drugs bypassing the DBP. Top view on the AcrB porter domain. Only the α-helical part of the PC1 and PC2 subdomains is shown (blue, L protomer; yellow, T protomer, red, O protomer). Binding of rifampicin-type (top row) and rifabutin/macrolide-type (bottom row) drugs to the access pocket (AP) is shown from left to right. The switch loop is colored in magenta. The dotted boundary of the ovals represents a closed deep binding pocket (DBP). Red arrow: the movement of switch loop. For further discussion on the mechanism see main text and Supplementary Note 2.5. PDB IDs of the displayed protomer structures: L, O: 4DX5; L^3-FOR^: 6ZOB; L^ERY^: 6ZOC; T^LIG1^: 6ZO7; T^LIG2*^, T^LIG3^: 6ZO9.

Based on this quite unique structural insight, we propose that HMW drugs like macrolides and ansamycins are initially captured in the AP of the L protomer (via CH2) where the switch loop accommodates their binding (Fig. 5 and Supplementary Fig. 3a–c; Supplementary Note 2.5). We assume that the Gly619Pro and Gly621Pro substitutions induce a switch loop conformation mimicking a transition state during wild-type L to T conformer conversion while the HMW drug is bound to the AP. During the L to T transition, the HMW drug accommodates in the AP/DBP region, mediated by the flexible switch loop of the T protomer, in an induced fit manner (Fig. 5 and Supplementary Fig. 3d–h). The intermediate positions of the ansamycins observed in these structures appears to precede the binding to the DBP as has been shown for erythromycin in the MtrD homolog RND pump from *Neisseria gonorrhoeae*^26^. During the T to O transition, these drugs are then exported via the DBP area through the O protomer exit tunnel (Fig. 5). The binding of multiple substrates^10,12^ (Fig. 4b and Supplementary Fig. 2a–f) suggest a possible role in the observed cooperativity in presence of more than one drug^20,27^. Hence, we started to experiment with antibiotic cocktails to observe multidrug binding to AcrB.

### Transmembrane domain allosteric substrate binding

**Fig. 6:**
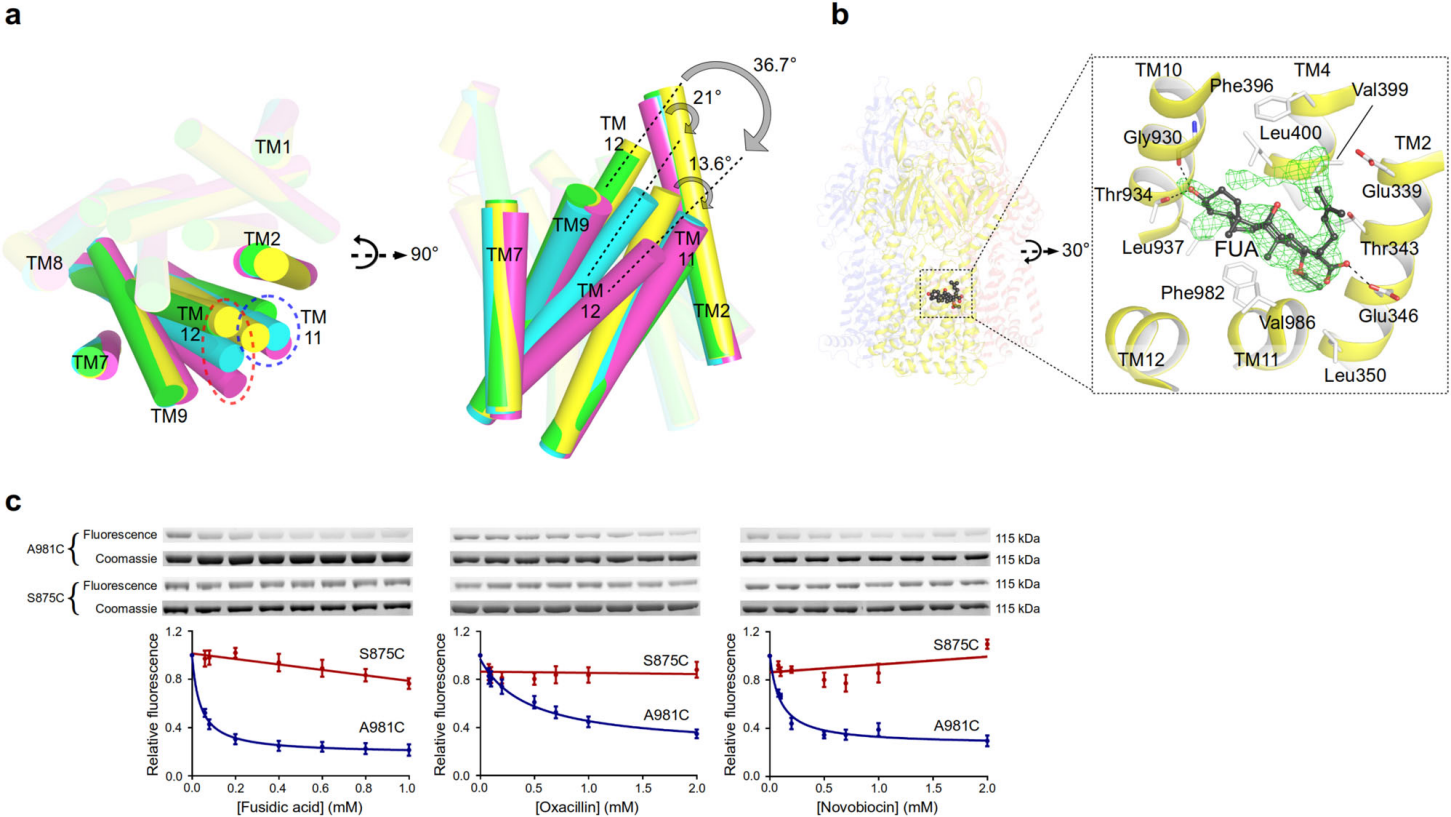
Deep transmembrane domain-binding pocket (TMD-BP) and a model for allosteric drug binding and transport. **a** Conformational changes of TM11/TM12 in the transmembrane domain of the T protomer induced by drug binding (yellow, unliganded T; cyan, partially induced T in presence of β-lactams; green, partially induced T of 3-FOR bound Gly619Pro; magenta, fully induced T in presence of fusidic acid (FUA) bound to the TMD-BP. **b** FUA (carbon, black; oxygen, red) binding to TMD-BP of the T protomer (yellow). Inset: Polder electron density map assigned to FUA is contoured at 4.5 σ. **c** Substrate protection experiment of AcrB-cl_Cys981 and AcrB-cl_Cys875 (negative control) by in-gel fluorescence of MTS-rhodamine modified proteins in dependence of the drug concentration (from left to right increasing concentration of drugs). Data are presented as mean ± s.e.m. of *N* ≥ 4 independent experiments. The calculated apparent *K*_i_^app^ for fusidic acid, oxacillin, or novobiocin is 38, 446, or 95 μM, respectively (Source Data Fig. 6c).

We used several drug combinations for co-crystallization assays, and co-crystallization of wild-type AcrB with an antibiotic cocktail of erythromycin, linezolid, oxacillin, and FUA (1 mM each) led to the elucidation of a co-structure with FUA bound to a deep concave crevice surrounded by TM2, TM4, TM10-12, and near the Asp407/Asp408/Lys940 proton relay residues^6–9^ (Fig. 6a, b and Supplementary Fig. 4a; Supplementary Table 1b; Supplementary Note 3). This was unexpected, since the TMDs of the AcrB protomers are known thus far to exclusively facilitate the transport of protons via the proton relay residues^3,15^.

The structure displays a substantial displacement of TM11 and TM12 (~13.6° and ~36.7° tilting, respectively, Fig. 6a) towards the periphery of the TMD in the T protomer creating a ~1700 Å^3^ cavity to accommodate FUA (we call this cavity the deep TMD binding pocket, TMD-BP) (Fig. 6a, b and Supplementary Fig 4b). No major conformational changes are observed in the L and O protomer (Supplementary Table 1a). Interestingly, two further FUA molecules bind to this T protomer as well. One FUA binds to a site akin to the previously reported TM1/2 groove site^19,25^ (Supplementary Fig. 2j, k). The other FUA molecule binds inside the TM7/TM8 groove (Fig. 2e), similar to the binding of FUA to the Phe380Ala variant (as discussed above, Fig. 2d), but deeper into the TMD core (Fig. 2f). Most peculiar, under FUA-only co-crystallization conditions leading to binding of FUA only to the TM1/TM2 groove^25^, high FUA concentrations (4 or 5 mM) were mandatory. In the structure presented here, FUA, erythromycin, oxacillin, and linezolid were only present at 1 mM concentration each, and we observe binding of FUA at the TM1/TM2 groove, the TM7/TM8 groove and TMD-BP. These observations suggest allosteric drug binding, and therefore the observed TMD-BP is proposed to be an allosteric site. A structure derived from AcrB co-crystallization with a mixture of β-lactams (dicloxacillin, oxacillin, and piperacillin), resulting in the structure with the DDM-bound L2 protomer (see above) displays a similar, albeit less extensive movement of TM11 in the T protomer. This results in a notable, but smaller cavity of ~304 Å^3^ within the T protomer (Supplementary Fig. 4c). We propose that the latter structure represents a state prior to binding of drug in the TMD-BP (partially induced vs. fully induced in case of drug binding). The putative TMD-BP was probed using MTS-rhodamine (MTS-R), a thiol-reactive rhodamine crosslinker^25^. Cys-substitution of Ala981 in an otherwise active Cys-less AcrB background^28^ was selected for probing AcrB variants. The Ala981Cys variant showed no compromised activity to all the tested drugs (Supplementary Data 2c; Supplementary Fig. 7f). MTS-R, but not *N*-(1-pyrenyl) maleimide (P-MAL)^29^ reacts efficiently with Cys981 (*K*_*d*_^app^ = 2.68 μM, Supplementary Fig. 4e), indicating that the putative allosteric TMD-BP is specific towards MTS-R. A concentration-dependent reduction of MTS-R labelling of C981 in presence of FUA, oxacillin, or novobiocin (*K*_i_^app^ = 38, 446, or 95 μM, respectively) is observed (Fig. 6c). Linezolid and chloramphenicol were unable to protect against MTS-R labelling, whereas erythromycin induced a concentration-dependent increased labeling of Cys981 (Supplementary Fig. 4f). The latter observation might be due to erythromycin-specific TMD alteration of AcrB and thereby facilitation of MTS-R binding.

**Fig. 7:**
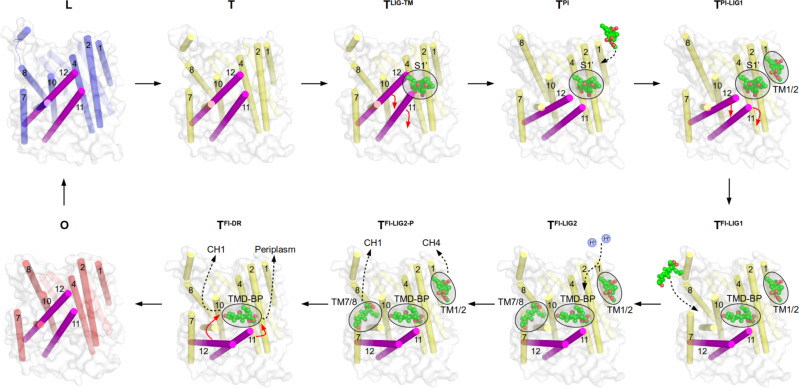
Proposed allosteric binding of fusidic acid to the transmembrane domain-binding pocket (TMD-BP) upon conformational changes of TM11/TM12 (magenta). Concomitant cooperative binding of further fusidic acid (FUA) molecules (spheres) is indicated. Binding of FUA at the TMD-BP is suggested to facilitate H^+^-translocation in the T protomer transmembrane domain (TMD) triggering the T to O transition. Structural differences between the T protomer TMDs in T^PI^ and T^FI^ states are accompanied with changes in the PC1 and PC2 subdomains reflecting intermediate in-between T and O states (Supplementary Fig. 4d; Supplementary Note 3). PDB IDs of the displayed protomer structures: L, T, T^LIG-TM^, O: 4DX5; T^PI^, T^PI-LIG^: 6ZOA; T^FI^: 6ZOD. The ligands of T^LIG-TM^, T^PI^, T^PI-LIG1^, are modelled and not part of the indicated PDB structures.

Substitution of TMD-BP residues in contact with FUA such as Leu400 (TM4) results in severe increased susceptibilities for phenicols, β-lactams, linezolid, FUA, erythromycin, and to a much lesser degree novobiocin (Supplementary Data 2c; Supplementary Fig. 7f); however, there is no change in susceptibility towards doxorubicin and R6G. Thr934Ala and Leu937Ala (both TM10) show severe susceptibility effects for β-lactams, FUA, novobiocin, and erythromycin, moderate for phenicols (Thr934Ala only), and linezolid (Supplementary Data 2c; Supplementary Fig. 7f). Doxorubicin, R6G, and tetraphenylphosphonium (TPP) susceptibilities were not (Thr934Ala) or mildly to moderately (Leu937Ala) affected. These complex phenotypes can be viewed in the line of (a) a general loss of activity, which can be excluded since doxorubicin and R6G (and for most substitutions also TPP) are showing in most cases wild-type susceptibilities (Supplementary Data 2c; Supplementary Fig. 7f). A second interpretation (b) assumes that the observed TMD-BP is a substrate binding site for the drugs to access the TM1/TM2 or TM7/TM8 grooves, from where the drugs are further transported to the drug-binding AP and DBP. A third option (c) is to assume an allosteric binding site (Fig. 7). We only observed the binding of additional FUA molecules to both of the TM1/TM2 or TM7/TM8 grooves in case FUA was bound to the TMD-BP. Therefore, binding of drugs to the TMD-BP might facilitate initial binding of other drugs to other sites. Furthermore, the TMD-BP exposes a large cavity accessible from the periplasmic side towards the Asp407/Asp408/Lys940 proton relay residues. Binding of drugs near these residues and tilting of TM11/12, as observed in the FUA-bound structure, might be a facilitator for the binding of protons to the Asp407 and/or Asp408 protonation sites or the subsequent conformational change after proton binding, leading to a strong increase in pump activity. This allosteric hyperactivation of the pump might be suitable in case drug concentrations reach levels affecting the integrity of the IM, which would impede the survival of the cell. Since drug efflux pumps like AcrAB-TolC consume a considerable amount of energy (proton-motive force) in presence of drugs^25^, allosteric regulation of its activity might represent a resource-saving measure.

Our results highlight the flexibility of the AcrB RND transporter to recognize, bind, and transport multiple drugs. It employs multiple binding sites and pathways comprising different substrate specificities resulting in the observed polyspecific drug resistance phenotype. Moreover, the H^+^-conducting transmembrane domain of AcrB might include an allosteric TMD-BP which, if occupied with drug, may cause hyperactivation or even cooperative multidrug transport.

## Methods

### Bacterial strains and growth conditions

*E. coli* MachT1 (Life Technologies) cells were routinely used for cloning. *E. coli* C43 (DE3)^30^ Δ*acrAB* harbouring pET24acrB_His_ was routinely grown in LB medium in the presence of 50 μg ml^−1^ kanamycin at 37 °C^31,32^.

### Cloning of *acrB* gene and site-directed mutagenesis

The *acrB* gene was amplified from chromosomal *E. coli* DNA with primers listed in Supplementary Table 2. The amplified *acrB* gene was cloned into pET24a (Novagen) via NdeI and XhoI restriction sites^31^. Site-directed mutagenesis was achieved using the ExSite protocol (Stratagene) with 5′-phosphorylated primers (Supplementary Table 2). All the plasmids were verified by sequencing (Eurofins).

### Overproduction and purification of DARPins

A single colony of *E. coli* XL1-Blue cells harbouring pQE30-DARPin^9,32^ was used to inoculate LB supplemented with 50 mg ml^−1^ kanamycin at 37 °C and cultivated overnight. Overnight cultures were used to inoculate fresh LB supplemented with antibiotic as above. Gene expression was induced by addition of 0.5 mM (final concentration) isopropyl-β-D-thiogalactoside at OD_600_ of 0.7 and the induced cultures were grown overnight at 37 °C. Cells were harvested by centrifugation and suspended in 50 mM Tris-HCl buffer at pH 7.5, 400 mM NaCl, and 10 mM Imidazole). Cells were lysed by a Pressure Cell Homogeniser (Stansted Fluid Power Ltd, United Kingdom) at 15,000 psi and cleared by centrifugation at 160,000 × *g* for 1 h. Supernatant was loaded onto a HisTrap HP Ni^2+^ affinity column (5 ml bed volume, GE Healthcare). After two wash steps with the same buffer supplemented with 20 mM and 50 mM of imidazole, DARPin proteins were eluted with 50 mM Tris-HCl buffer at pH 7.5, 400 mM NaCl, 250 mM Imidazole and 10% Glycerol).

### Overproduction and purification of AcrB and AcrB mutants

*E. coli* C43 (DE3) Δ*acrAB* harbouring pET24acrB_His_^31,32^ (WT or various AcrB variants, respectively) was grown overnight in LB or Terrific Broth supplemented with 50 mg ml^−1^ kanamycin at 37 °C. Overnight cultures were inoculated into fresh LB or Terrific Broth supplemented with antibiotic as above and grown until OD_600_ of 0.8 before 0.5 mM (final concentration) isopropyl-β-D-thiogalactoside was added to the culture. Cultures were grown at 20 °C for another 16 h and cells subsequently harvested. The cell pellet was resuspended in Buffer A (20 mM Tris-HCl at pH 8.0, 500 mM NaCl, 2 mM MgCl_2_, and 0.2 mM diisopropyl fluorophosphate) and lysed by a Pressure Cell Homogeniser (Stansted, United Kingdom). Cell debris was removed by centrifugation at 23,000 × *g* for 15 min and cell membranes were collected by centrifugation at 160,000 × *g* for 2 h. Cell membranes were suspended in Buffer B [20 mM Tris/HCl buffer at pH 7.5, 150 mM NaCl, 20 mM Imidazole, and 10% Glycerol and solubilized with 1% dodecyl maltoside (D-97002-C, DDM, Glycon)] at 4 °C for 1 h. Solubilized membranes were cleared at 160,000 × *g* for 30 min and the supernatant was loaded onto a HisTrap HP Ni^2+^ affinity column (1 mL bed volume, GE Healthcare). The column was washed twice with Buffer B supplemented with 0.02% DDM in addition to 60 mM or 90 mM imidazole, respectively. AcrB protein was eluted with Buffer D (20 mM Tris/HCl at pH 7.5, 150 mM NaCl, 220 mM Imidazole, 10% Glycerol, and 0.02% DDM).

### Crystallization of AcrB/DARPins

Before setting up crystallization, AcrB was mixed with DARPins^9,32^ in a molar ratio of 1:2 and incubated on ice for 15 min. Excess DARPins were removed by size-exclusion chromatography (Superose 6, GE Healthcare) with buffer containing 20 mM Tris pH 7.5, 150 mM NaCl, 0.03% DDM, and 0.05% DDAO (Anatrace). Co-crystallization of AcrB/DARPins with various substrates were obtained by hanging drop crystallization within 1–2 weeks. Briefly, AcrB substrates (wild-type AcrB fully induced T protomer, 1 mM erythromycin + 1 mM fusidic acid + 1 mM linezolid + 1 mM oxacillin; wild-type AcrB L2-T-O protomer, 1 mM dicloxacillin + 1 mM oxacillin + 1 mM piperacillin; 3-formylrifamycin SV bound wild-type AcrB, 3 mM rifampicin; minocycline bound Ile38Phe-Ile671Thr, 2 mM minocycline; erythromycin and 3-formylrifamycin SV bound Gly616Pro, 3 mM rifampicin + 1 mM erythromycin + 1 mM fusidic acid + 1 mM linezolid + 1 mM oxacillin; 3-formylrifamycin SV bound Gly619Pro, 3 mM rifampicin quinone + 1.2 mM minocycline; minocycline bound Gly619Pro, 1.2 mM minocycline; minocycline bound Gly621Pro, 2 mM minocycline; rifabutin bound Gly621Pro, 3 mM rifabutin; 3-formylrifamycin SV bound Gly619Pro_Gly621Pro, 3 mM rifampicin quinone; fusidic acid bound Gly619Pro_Gly621Pro, 1 mM erythromycin + 1 mM fusidic acid + 1 mM linezolid + 1 mM oxacillin) were mixed with protein solution (13 mg/ml in final concentration), respectively, and subsequently, all the mixtures were centrifuged at 13,000 × *g* for 10 min at 4 °C to remove the insoluble materials, before setting up the crystallization. The reservoir contained 50 mM ADA pH 6.6, 5% Glycerol, 8-9% PEG4000 and 110–220 mM ammonium sulfate. Fusidic acid bound Phe380Ala was obtained by soaking unliganded crystals with 5 mM fusidic acid for 4–5 days. Crystals were cryo-protected by serial transfer of crystals into reservoir supplemented with increasing of glycerol concentration to 28% before flash-cooling in liquid nitrogen. Crystals of AcrB-Phe563Ala in the presence of DARPins were obtained by sitting drop vapor diffusion method within 1–2 weeks by equal volume of the protein solution (12 mg ml^−1^) and precipitant solution containing 0.1 M sodium acetate, pH 5.5 and 12% PEG400. Crystals were cryo-protected into reservoir supplemented with increasing PEG400 concentrations up to 20% PEG400 before flash-cooling in liquid nitrogen.

### Diffraction data collection and refinement

Data were collected on beamline PROXIMA 1 (Pilatus 6 M or Eiger X 16 M detector), PROXIMA 2 A (ADSC Q315r Area or Eiger X 9 M detector), Soleil Synchrotron, Saint Aubin, France, P13 (Pilatus 6 M detector), Petra III, Deutsches Elektronen Synchrotron, Hamburg, Germany or X06DA (Pilatus 6 M detector), Swiss Light Source, Paul Scherrer Insitute, Villigen, Switzerland, indexed and integrated with XDS^33^. The dataset of AcrB-G616P structure in complex with erythromycin and 3-formyrifamycin SV was processed by STARANISO server (Global Phasing). All the structural models were iteratively built in COOT^34^ and refined with REFMAC5^35^ within CCP4i package^36^, phenix.refine^37^ within Phenix package^38^ or BUSTER (Global Phasing)^39^. The structure was validated with MolProbity^40^. Polder maps were calculated by phenix.polder^41^ within Phenix package^38^. All figures were generated by Pymol (Schrödinger, LLC). Tunnels and cavities were calculated using Caver^42^. Data collection and refinement statistics are listed in Supplementary Data 3.

### Thermal shift assay/differential scanning fluorimetry

Differential scanning fluorimetry (DSF) was performed according to Alexandrov et al.^43^ with Rotor-Gene Q instrument (Qiagen, Hilden, Germany). The thiol-reactive fluorochrome N-[4-(7-diethylamino- 4-methyl-3-coumarinyl)phenyl]maleimide CPM dye (Sigma, Ex: 380 ± 20 nm; Em: 460 ± 20 nm) was used to study the thermal denaturation of AcrB and variants (0.1 mg ml^−1^) in the presence and absence of the AcrB inhibitor MBX3132^10^ (250 μM final concentration). Protein (±inhibitor) sample (59 ul, 0.1 mg ml^−1^) + 1 ul CPM (10 mg/ml) was incubated at RT for 2 min before centrifugation of the sample at 13,000 × *g* for 10 min at 4 °C. Supernatant (25 ul) was transferred into a PCR tube and thermal denaturation scanning was conducted between 25 and 80 °C with 1 °C/20 s. After collection of data, the fluorescence intensity was plotted against temperature and differential plot (dF (fluorescence)/dT (temperature)) of the curve produced a peak representing protein denaturation state with respect to temperature. The highest point on the peak was defined as the melting temperature (T_m_ in °C) of the protein.

### Drug agar plate assay

Drug agar plate assay was performed as previously described^19^. A colony of *E. coli* BW25113 ∆*acrB* harboring pET24*acrB*-His wild-type and AcrB variants were grown overnight in LB containing 50 μg ml^−1^ kanamycin at 37 °C. Dilution of the cultures to OD_600_ 10^−1^–10^−6^ were prepared and 4 μl of each diluted cultures were spotted on an LB agar plate containing 50 μg ml^−1^ kanamycin, supplemented with AcrB drugs (0.75–1.0 μg ml^−1^ chloramphenicol, 12.0–18.0 μg ml^−1^ clarithromycin, 28.0–50.0 μg ml^−1^ cloxacillin, 60.0–95.0 μg ml^−1^ dicloxacillin, 10.0–24.0 μg ml^−1^ dirithromycin, 12.0–15.0 μg ml^−1^ doxorubicin, 7.0–20.0 μg ml^−1^ erythromycin, 0.75–1.0 μg ml^−1^ florfenicol, 17.0–30.0 μg ml^−1^ 3-formylrifamycin SV, 12.5–18.0 μg ml^−1^ fusidic acid, 12.0–16.0 μg ml^−1^ linezolid, 8.0–10.0 μg ml^−1^ novobiocin, 70.0–120.0 μg ml^−1^ oleandomycin, 20.0–30.0 μg ml^−1^ oxacillin, 0.12–0.20 μg ml^−1^ piperacillin, 25.0 μg ml^−1^ rhodamine 6 G, 15.0–70.0 μg ml^−1^ roxithromycin, 3.0–4.5 μg ml^−1^ rifabutin, 18.0–26.0 μg ml^−1^ rifamycin SV, 3.5–5.0 μg ml^−1^ rifaximin, 15.0–100.0 μg ml^−1^ spiramycin, 18.0–20.0 μg ml^−1^ thiamphenicol, 80.0–130.0 μg ml^−1^ TPP, 15.0–80.0 μg ml^−1^ tylosin). Plates were incubated at 37 °C for 14–16 h. Drug agar plates were imaged by ImageQuant (GE Healthcare BioSciences AB, Uppsala, Sweden). The intensity of the cell growth was quantified by ImageJ 1.52o software. The cell growth of WT at first dilution is set to 1. The intensity of cell growth at each dilution step higher than 10% of cell growth of WT at first dilution is considered as cell growth. To calculate the relative cell growth, the cell growth of each mutant is normalized with the cell growth of WT (e.g. if the cell growth of WT is four dilution steps, the fourth dilution step is set to 100% growth). Growth of AcrB variants lower than 75% compared to wild-type AcrB growth is considered significantly affected if *p* value ≤ 0.005 or slightly affected if *p-*value = 0.005–0.05. Growth of AcrB variants in between 75 and 85% of growth of wild-type AcrB is considered as slightly affected if *p-*value ≤ 0.005. *p-*value was calculated by two-sided Student’s *t*-test.

### Agar plate assay supplemented with DDM

A colony of *E. coli* BW25113 ∆*acrB* harboring pET24acrB_His_ wild-type or AcrB variants were grown overnight in LB containing 50 μg ml^−1^ kanamycin at 37 °C. Dilution of the cultures to OD_600_ 10^−3^–10^−8^ were prepared and 1.5 μl of each diluted cultures were spotted on an LB agar plate containing 50 μg ml^−1^ kanamycin, supplemented with 56 mg ml^−1^ DDM or 12 μg ml^−1^ doxorubicin. Plates were incubated at 30 °C for 14–16 h. Drug agar plates were imaged by ImageQuant TL (GE Healthcare BioSciences AB, Uppsala, Sweden).

### Western blot analysis

Western blot analysis of the production of AcrB or AcrB variants was performed as previously described^25^. Briefly, overnight cultures were suspended in 20 μl of Lysis Buffer (50 mM Tris-HCl, pH 7.5, 500 mM NaCl, 10% Glycerol) to obtain an OD_600_ = 15.0, and subsequently, incubated with 2× SDS-lysis buffer. The cell suspensions were incubated at 95 °C for 10 min. The cell lysates were centrifuged at 13,000 × *g* for 10 min. The supernatant was resolved by 12.5% SDS-PAGE gels and transferred onto nitrocellullose membrane. The membrane was incubated with anti-AcrB antibody (dilution of 1:10,000; Neosystems, France, custom-antibody) and then, with anti-rabbit IgG (whole molecule)-alkaline phosphatase antibody (dilution of 1:1,500; A3687, Sigma–Aldrich, St. Louis, USA). Finally, the blot development was performed with NBT (nitro-blue tetrazolium chloride) and BCIP (5-bromo-4-chloro-3’-indolyphosphate p-toluidine salt). The wild-type AcrB and all the substitution variants except AcrB-Tyr35Ala, show equal expression/production in *E. coli* BW25113(DE3) Δ*acrB* harbouring the complemented gene on a plasmid. Western blot analysis results are given in Source Data Supplementary Data 1 and 2.

### Substrate(s) protection cross-linking assay

AcrB cysteine-less variant proteins with amino acid substitution of Ala981Cys (cl_Cys981) or Ser875Cys (cl_Cys875) were purified with the same procedure as shown above until Ni^2+^-affinity chromatography. Finally, cl_Cys981 or cl_Cys875 was purified by size-exclusion chromatography (Superose 6, GE Healthcare) with buffer containing 20 mM Tris pH 7.5, 150 mM NaCl, 0.02% DDM. To determine the efficiency of MTS-rhodamine labeling to cl_C981 located at the TMD-BP, approximately 2 μM cl_Cys981 protein was incubated with various concentration of MTS-rhodamine (0.1, 0.3, 0.5, 0.7, 1.0, 2.0, 5.0, and 10.0 μM) in total reaction of 100 μl in Protein Cross-linking Buffer (PCB, 20 mM Tris/HCl, pH 7.1, 150 mM NaCl, 0.02% DDM) and incubated on ice for 5 s. Subsequently, the reaction was diluted with 100 μl ice-cold PCB in addition of 2 mM *N*-ethylmaleimide (NEM) (final concentration of NEM is 1 mM in the final mixture) to stop the reaction. For *N*-(1-pyrenyl) maleimide (P-MAL) experiment, 2 μM cl_Cys981 protein was incubated with various concentration of P-MAL (0.1, 0.3, 0.5, 0.7, 1.0, 2.0, 5.0, and 10.0 μM) in total reaction of 100 μl in PCB buffer and incubated at room temperature for 10 min. Subsequently, the reaction was diluted with 100 μl ice-cold PCB in addition of 20 μM MTS-rhodamine (final concentration: 10 μM of MTS-rhodamine in the final mixture) and incubated at room temperature for 5 min. For substrate protection experiment, 2 μM of each AcrB variants, cl_Cys981 or cl_Cys875, were mixed with various concentration of AcrB substrates (erythromycin, fusidic acid, linezolid, novobiocin, oxacillin, and chloramphenicol) in total reaction of 100 μl in PCB buffer and incubated at 22 °C for 3 h. Subsequently, 0.5 μM of MTS-rhodamine was added to the reaction and incubated on ice for 10 s. The labelling reaction was stopped by addition of 100 μl ice-cold PCB in the presence of 2 mM NEM (final concentration: 1 mM NEM the reaction mixture). Immediately, all samples were mixed with 2× Laemmli sample buffer in the presence of 2 mM NEM and subjected to SDS-PAGE analysis. The fluorescence signal was detected on an ImageQuant LAS 4000 [Excitation with Epi-Green (Cy3) and emission filter of 575DF20 (Cy3)] (GE Healthcare BioSciences AB, Uppsala, Sweden) before staining with Coomassie Brilliant Blue. All images were analyzed with ImageQuant TL 8.1 software. The relative fluorescence with the signal in the absence of drugs set to 1 and normalized with the quantified Coomassie stained protein fragments (Source Data Fig. 6c, Source Data Supplementary Fig. 4e, f). The apparent inhibition constant of MTS-rhodamine labeling for fusidic acid, oxacillin, or novobiocin was calculated by fitting the curves to the nonlinear regression fit with one-site saturation binding function using GraphPad Prism 7.0.

### Reporting summary

Further information on research design is available in the Nature Research Reporting Summary linked to this article.

## Supplementary information

Supplementary Information

Peer Review File

Description of Additional Supplementary Files

Supplementary Data 1

Supplementary Data 2

Supplementary Data 3

Reporting Summary

## Data Availability

Atomic coordinates and structure factors reported in this paper have been deposited in the Protein Data Bank under accession numbers 6ZO5 (AcrB-Gly619Pro_Gly621Pro/DARPin in the presence of fusidic acid), 6ZO6 (AcrB-Gly619Pro/DARPin in the presence of minocycline), 6ZO7 (AcrB-Gly619Pro/DARPin in the presence of 3-formylrifamycin SV), 6ZO8 (AcrB-Gly621Pro/DARPin in the presence of minocycline), 6ZO9 (AcrB-Gly621Pro/DARPin in the presence of rifabutin), 6ZOA (AcrB/DARPin L2-T-O conformation with DDM bound to TM8/PC2 tunnel), 6ZOB (AcrB/DARPin in the presence of 3-formylrifamycin SV), 6ZOC (AcrB-Gly616Pro/DARPin in the presence of erythromycin and 3-formylrifamycin SV), 6ZOD (AcrB/DARPin in the presence of fusidic acid, fully induced T state), 6ZOE (AcrB-Phe563Ala/DARPin), 6ZOF (AcrB-Phe380Ala/DARPin in the presence of fusidic acid), 6ZOG (AcrB-Ile38Phe_Ile671Thr/DARPin in the presence of minocycline), and 6ZOH (AcrB-Gly619Pro_Gly621Pro/DARPin in the presence of 3-formylrifamycin SV). Atomic coordinates that support the findings of this study are available in the Protein Data Bank under accession numbers [2J8S], [3AOC], [3W9I], [4DX5], [4ZJL], [5JMN], [6ZO5], [6ZO6], [6ZO7], [6ZO8], [6ZO9], [6ZOA], [6ZOB], [6ZOC], [6ZOD], [6ZOE], [6ZOF], [6ZOG], and [6ZOH]. Source Data for Fig. 6c, Supplementary Fig. 1b, Supplementary Fig. 4e, f, and Supplementary Data 1-2 are provided with this paper. Source data are provided with this paper.

## Source data

Source Data
