# Supplementary material for: Allosteric drug transport mechanism of multidrug transporter AcrB
Source: Nat Commun. 2021 Jun 29;12:3889. doi: 10.1038/s41467-021-24151-3 (PMC8242077; doi:10.1038/s41467-021-24151-3)
Supplement: Supplementary file 1 — Supplementary Information [file 41467_2021_24151_MOESM1_ESM.pdf]

Supplementary Information

Allosteric drug transport mechanism of multidrug transporter AcrB

Heng-Keat Tam[1,§,†,*], Wuen Ee Foong[1,§], Christine Oswald[1#], Andrea Hermann[1], Hui Zeng[1] and Klaas M. Pos[1*]

[1]Institute of Biochemistry, Goethe-University Frankfurt, Max-von-Laue-Str. 9, D-60438 Frankfurt am Main, Germany.

[#]present address: Sosei Heptares, Steinmetz Building, Granta Park, Great Abington, Cambridge, CB21 6DG, UK.

†present address: Hengyang Medical College, University of South China, Hengyang 421002, Hunan Province, China.

[§]Contributed equally

*Corresponding author

Heng-Keat Tam (tamhk60@hotmail.com)
Klaas M. Pos (pos@em.uni-frankfurt.de)

Supplementary Note 1. Substrate access from the membrane towards the deep binding pocket (DBP)

Supplementary Note 1.1 AcrB structure

Inner membrane proteins of the resistance nodulation and cell division (RND) superfamily are ubiquitously present in all domains of life[1]. In Gram-negative bacteria, these transporters have been characterized as H^+/solute antiporter efflux pumps which are only active in the presence of a periplasmic adaptor protein (PAP) and an outer membrane channel/factor (OMF) (Fig. 1a). Several pump systems have been characterized and many are involved in the resistance against multiple drugs. A single pump system recognizes and exports multiple drugs, often including antibiotics from different classes; these pumps are therefore considered polyspecific. Every Gram-negative bacterium comprises genes encoding at least one of these pump systems, *E. coli* K12 encodes 5, *Pseudomonas aeruginosa* PAO1 encodes 12 of these drug pumping systems[2] (hydrophobe/amphipathic exporter family, HAE-1, www.membranetransport.org). The inner membrane RND pump AcrB is a H^+/drug antiporter component of the main multidrug resistance pump AcrAB-TolC in *Escherichia coli*. AcrA is the PAP, and TolC the OMF (Fig. 1a). Homotrimeric AcrB comprises three protomers, and each protomer consists of a transmembrane domain (TMD), a periplasmic porter domain (PD) and a periplasmic funnel domain (FD) (Fig. 1b). During a drug transport cycle, the three protomers adopt three different conformations, which are designated Loose (L), Tight (T) and Open (O), or Access, Binding, and Extrusion conformation[3,4]. In the PD, two multidrug binding pockets have been characterized, the proximal access pocket (AP) in the L conformation and the distal deep binding pocket (DBP) in the T conformation[3,5,6] (Fig. 1c). Of note, also in T conformation an AP is available for drug binding, but this pocket is less voluminous compared to the AP in the L conformation. In the

O conformation, all pockets are closed, and an open exit channel guides the drugs into the AcrA and TolC channels towards the outside of the cell[4] (Supplementary Fig 5a).

The conformational changes from L to T is caused by initial drug binding to the L conformation (Supplementary Fig 5b). In the T conformation the DBP opens and allows drugs to bind at this location. In the drug-bound T conformation, the transmembrane domain (TMD) is open for water molecules to establish a water-network to facilitate H^+ transport toward the titratable residues D407 and D408 located inside the TMD[7]. Upon H^+ binding from the periplasm, D407 and D408 are protonated and cause a change in electrostatics in the TMD leading to the conformational change from T to O and drug efflux. Upon H^+ release to the cytoplasm, the protomer changes from O to L (Supplementary Fig 5b). The conformational changes elicited by drug binding in the DBP and H^+ binding in the TMD are transduced to the TMD and PD, respectively. The conformational changes in the PD are mediated by rigid body movements of the four subdomains, designated PN1, PN2, PC1 and PC2 (Fig. 1c). The subdomains are connected via loop regions, including the connecting c-loop between the PC1 and PC2 subdomain (Fig. 1c). The flexibility of the c-loop is important for the binding of dodecyl β-D-maltoside (DDM) as shown in Fig. 2a.

Supplementary Note 1.2 Channel 1 (CH1) and the TM8/PC2 pathway structural analysis

The DDM-bound L2 protomer within the asymmetric L2-T-O AcrB trimer adopts an L-to-T transition state (Fig. 2a). Structural comparison of the AcrB L and L2 protomers shows the conformational change of TM8 and the connecting c-loop (residues 668-678, Fig. 1c) to accommodate the DDM in a transient state of channel 1 (CH1). We call this transient tunnel, the TM8/PC2 tunnel, because the tunnel starts at TM8 in the TMD and follows a path along the PC2 subdomain in the periplasmic part of AcrB (Fig. 2a). The conformational change from the L into the L2 state is most prominent for the c-loop residues Ile671 and Leu674, moving 6.0 Å (C_α) or 8.9 Å, respectively, from their position in

the L protomer (Supplementary Fig 1a). Leu674 appears therefore to be a trigger residue for opening the PC2/TM8 pathway. The c-loop itself moves from a TM8 proximal orientation to a distal one and closes in on the PN1-PN2 loop (residues 131-137, Fig. 1c) distance reduction ~5 Å between Cα of Glu673 (c-loop) and Ser135 (PN1-PN2 loop) (Fig. 2c). Binding of DDM in the PC2/TM8 tunnel is mainly contributed by van der Waals interactions (Fig. 2b). Even though hydrophilic residues are lining this tunnel, the polar side chains of hydrophilic residues like Thr463, Gln865, Glu866, Ser869, and Glu673 are in defined rotamer states such that the acyl chain of DDM is in van der Waals contact with their side chain carbon atoms (Fig. 2b). The overall conformational difference compared to AcrB in the conventional LTO state[3,6] is most apparent for the L protomer (rmsd: 1.1 Å), but also prominent for the T protomer (rmsd: 1.1 A) (Supplementary Table 1a).

Supplementary Note 1.3 Channel 1 (CH1) and the TM8/PC2 pathway functional analysis

Strikingly, a clear substrate preference for the TM8/PC2 pathway (channel 1, CH1, Supplementary Fig 1a) toward low molecular weight (LMW)/low polar surface area (LPSA) drugs (e. g. β-lactams, linezolid, and phenicols) was observed. Approximately 60% of the selected single-alanine AcrB substitution variants lining this pathway affect β-lactam, linezolid, and phenicol susceptibility (Supplementary Data 1a, b; Supplementary Fig 7a, b). Ala-substitution of residues Gln34, Ile38, Thr93, Phe453, Ile466, Phe470, Phe563, Leu564, Glu567, Ile671, Leu674, Ser863, Gln865, Glu866, Phe927, or Val929, together with residues involved in fusidic acid binding to the TM7/TM8 groove (discussed later), all cause increased susceptibility towards β-lactams, linezolid, and phenicols. Substitutions Ile466Ala, Tyr467Ala, Phe563Ala, Ile671Ala, Asp924Ala, or Tyr926Ala variants in addition caused susceptibility toward erythromycin, fusidic acid, novobiocin, and/or tetraphenylphosphonium. All substitution variants showed, however, no or minor growth inhibition effects in the presence of doxorubicin, rhodamine 6G, and/or tetraphenylphosphonium, indicating

that the results imply that the TM8/PC2 pathway is specifically important for the binding and/or transport of LMW/LPSA drugs.

Substitutions Tyr467Ala, Asp924Ala, and Tyr926Ala confer increased susceptibility towards β-lactams, linezolid, but not against phenicols (Supplementary Data 1a, b; Supplementary Fig 7a, b). These residues are located at the interface between the membrane located TM7/TM8 groove and the TM8/PC2 pathway. However, substitutions of residues below the interface region in the TM7/TM8 groove again render the cells more susceptible against both β-lactams and phenicols (Supplementary Data 1b; Supplementary Fig 7b). We therefore assume that phenicols are recruited via the TM7/TM8 groove and further transported through the TM8/PC2 pathway but might have different interactions compared to β-lactams when entering the TM8/PC2 pathway.

As has been shown previously[8] and confirmed here (Supplementary Data 1b; Supplementary Fig 7b), substitution of Ile38 and/or Ile671 causes a decreased resistance against LMW/LPSA drugs, whereas an increased resistance against clarithromycin[8] and also fusidic acid (Supplementary Data 1b; Supplementary Fig 7b) is observed. Ile38 is located on a flexible loop between the C-terminus of TM1 and the N-terminus of the PN1 subdomain (residues 30-41), and Ile671 is on the c-loop (Fig. 1c and Supplementary Fig 1e). Because of the pronounced effects caused by these substitutions, we structurally elucidated the Ile38Phe-Ile671Thr double substitution variant, which, however, does not show conformational difference compared to wildtype AcrB (Supplementary Table 1a).

The wildtype AcrB and all the AcrB variants described above and below, show equal expression/production in *E. coli* BW25113(DE3) Δ*acrB* harbouring the complemented gene on a plasmid, except the AcrB-Tyr35Ala variant (located at the same loop as Ile38) which was not expressed. Western blot analysis results are provided in Source Data Supplementary Data 1 and 2.

Supplementary Note 1.4 Guarding residues for CH1 entry

The Phe563 side chain at the entrance to CH1 appears to act as a guarding residue for further CH1 entry[9] (Fig. 2b, Supplementary Fig 1a). Structural analysis of trimeric AcrB-Phe563Ala reveals a rather unexpected TTT conformation (Supplementary Fig 1c, d). Due to a subtle shift of TM8 and the PC2 subdomain, the diameter of CH1 is slightly compromised, but more strikingly, the c-loop and in particular the Leu674 side chain appear to block the tunnel (Supplementary Fig 1d). Cells harbouring Phe563Ala are growth-compromised in presence of LMW/LPSA drugs, fusidic acid, and novobiocin (Supplementary Data 1b; Supplementary Fig 7b). Ala-substitution of juxtaposed Leu674 with Phe563Ala causes a fully inactive phenotype in the presence of LMW/LPSA drugs and DDM, but supports wildtype susceptibility against doxorubicin, erythromycin, and rhodamine 6G (Supplementary Data 1b; Supplementary Fig 7b).

Residue Phe563, located at the entrance of CH1, together with residues (Leu393, Ile466, Tyr467, Leu564, Ile671, and Leu674) located further along this channel, are important for drug transport (Supplementary Fig 1a, d, e; Supplementary Data 1a, b; Supplementary Fig 7a, b). Single-site substitution variant Phe563Ala specifically affects sensitivity towards LMW/LPSA drugs, fusidic acid, and novobiocin, however, partial activity was still observed. The more subtle sensitivity phenotype for this substitution variant towards fusidic acid, DDM and novobiocin might be explained that these drugs might use more than one substrate pathway in parallel (Supplementary Fig 1e; Supplementary Data 1; Supplementary Fig 7a-c). Compared to the Phe563Ala variant, the Phe563Leu variant restores the resistance of *E. coli* cells against fusidic acid and novobiocin, but not against LMW/LPSA drugs (Supplementary Data 1b; Supplementary Fig 7b), whereas substitution of Phe563 to bulky residues (e. g. tyrosine or tryptophan) fully restores the resistance phenotype against LMW/LPSA drugs. In combination with the juxtaposed substitution variants Leu564Ala or Leu674Ala high susceptibility against all drugs is observed, in comparison to the susceptibilities

conferred by single substitution variants (Supplementary Data 1b; Supplementary Fig 7b). This emphasizes the role of Phe563, Leu564, and Leu674 for drug transport via CH1. A double substitution variant Phe563Ala_Leu564Ala was shown to be completely devoid of phenicol, β-lactams and linezolid transport activity, whereas the transport of the other substrates, including the polyaromatic cations, was drastically reduced. This was indicative for a general transport or protein stability effect. This variant was tested upon its stability by purification and subjection to a thermal stability assay[10] and appeared thermally stable. It is further stabilized in presence of the high affinity inhibitor MBX3132[11] indicating the binding of the inhibitor, and correct folding of the protein (Supplementary Fig 6). Based on AcrB wildtype structures, it has been speculated that Phe563 at the entrance of CH1 might act as a gate/guardian residue[9]. This notion is supported by the Phe563Ala structure (Supplementary Fig 1d) and by the observation that other large hydrophobic residues at this position, like the Leu563, Trp563 or Tyr563 variants, are able to restore resistance in part or completely against the tested drugs in comparison to the Phe563Ala phenotype (Supplementary Data 1b; Supplementary Fig 7b).

Supplementary Note 1.5 TM7/TM8 groove as entrance for fusidic acid, β-lactams, linezolid, and novobiocin

The AcrB-Phe380Ala variant was selected for co-crystallization experiments as this CH4-variant confers greatly reduced susceptibilities towards carboxylated β-lactams[12] and reduced but substantial resistance against fusidic acid (Supplementary Data 1c; Supplementary Fig 7c). In this variant, fusidic acid was found to bind to the lower part of TM7/TM8 groove (Fig. 2d and Supplementary Fig 1f). We speculate that the binding of fusidic acid to TM7/TM8 groove is indirectly facilitated by the disrupted CH4 pathway due to the Phe380Ala substitution[12]. Hydrophobic residues in this fusidic acid binding region, including Val549, Tyr877, Leu881, Val884, Met902, Val905, and Ile935 are involved in the interaction with fusidic acid, but also Ser880, Tyr545. The main chain of Leu881 and

Met902 are interacting with fusidic acid via H-bonding (Fig. 2d). The binding of fusidic acid to TM7/TM8 groove is congruent to the binding of fusidic acid to the TM7/TM8 groove of the fully-induced T protomer (Fig. 2e, f). The Tyr545Ala or Leu888Ala variants infer increased susceptibility towards fusidic acid, as well as towards LMW/LPSA drugs and rhodamine 6G. Surprisingly, we observed in 50% of the cases when cells harbouring variants with substituted residues lining this groove, a change in susceptibility towards LMW/LPSA drugs, but not against doxorubicin, erythromycin, rhodamine 6G, and tetraphenylphosphonium, except for the Tyr467Ala (no effect on phenicol susceptibility, higher erythromycin susceptibility), Tyr545Ala (no effect on phenicol susceptibility), and Leu888Ala (higher rhodamine 6G susceptibility) variants (Supplementary Data 1a; Supplementary Fig 7a). Interestingly, alanine-substitution of Ser880 (on TM8) greatly increases the resistance of *E. coli* cells against fusidic acid but shows wildtype susceptibilities toward all other drugs. Ser880 is in hydrogen bonding distance to the carboxylic acid group of fusidic acid (Fig. 2d, e), so the loss of this hydrogen bonding partner is difficult to interpret with the increased resistance (as expression of this variant is comparable to the wildtype AcrB, (Source Data for Supplementary Data 1). Furthermore, the sensitivity of *E. coli* cells harbouring AcrB containing alanine-substitution of hydrophobic residues (Met552, Tyr877, Leu881, Val884, Leu888, Leu903, and Val905 (part of the fusidic acid binding site, Fig. 2e) against fusidic acid is not as pronounced compared to LMW/LPSA drugs (Supplementary Data 1a; Supplementary Fig 7a). Clearly, however, hydrophobic residues located at the TM7/TM8 groove are sensitive for alanine substitution and these substitution variants confer higher susceptibility against LMW/LPSA drugs, emphasizing the importance of this groove for sequestering phenicols, β-lactams, and linezolid (Fig. 1b).

Supplementary Note 1.6 CH1 and CH4 are complementary drug transport pathways

As was shown previously[12,13], β-lactams and fusidic acid are mainly transported by CH4 but single amino acid substitutions of CH4 do not lead to completely inactive AcrB variants for these substrates.

Combined substitutions of residues that are important in both CH1 and CH4 pathways (Phe563Ala or Tyr545Ala of CH1 and Asn298Ala, Val340Ala or Phe380Ala of CH4), however, reveal an inactive phenotype against β-lactams and fusidic acid (Supplementary Data 1c; Supplementary Fig 7c), indicating that fusidic acid and β-lactams are transported by both CH1 and CH4. Susceptibilities of cells harbouring these variants are not affected for doxorubicin, except for the double Asn298Ala_Phe563Ala variant, showing significant increased susceptibility against doxorubicin, erythromycin, and tetraphenylphosphonium which are presumably not transported via CH1 and CH4 (Supplementary Data 1c; Supplementary Fig 7c). This variant showed a slightly lower melting temperature compared to purified wildtype AcrB but was substantially stabilized by addition of the known inhibitor MBX3132[11] indicating correct folding and therefore the ability to bind the inhibitor (Supplementary Fig 6). We assume therefore, that this double substitution variant assumes a conformation which still allows drug/inhibitor binding but is completely defective in transport. For the other substitution variants, rhodamine 6G and tetraphenylphosphonium susceptibilities are affected for different substitution combinations, but not for any of the single substitutions of the substitution pairs. The CH1-CH4 phenotypes for β-lactams and fusidic acid might be explained by the observed connecting tunnel between CH1 and CH4 in the L2 protomer (Supplementary Fig 1e), indicating a disrupted transport pathway due to substitution of Phe563 (CH1) and Val340 or Asn298 (CH4). Similarly, Ala-substitution of Tyr545 (CH1, in hydrogen bond contact with fusidic acid (Fig. 2d), in combination with Phe380Ala (CH4) greatly affects β-lactam and fusidic acid susceptibility (Supplementary Data 1c; Supplementary Fig 7c). These observations are in line with the locations of the substitutions of residues identified by Kobayashi et al.[14] (in particular substitutions Q569R and E673G) and Tam et al.[12] (L300A and Y327A), showing distinct effects on β-lactam susceptibility.

In sum, our results indicate that phenicols, linezolid and presumably novobiocin are transported via CH1, whereas, β-lactams, DDM, and fusidic acid are transported by both CH1 and CH4 pathway[12,13] (Fig. 1b).

Supplementary Note 1.7 TM7/TM8 groove, CH1 and TM8/PC2 tunnel drug transport hypothesis

It has been shown that low molecular weight (LMW) drugs with high polar surface area (HPSA), and high planarity like tetracyclines (such as minocycline) and anthracyclines (like doxorubicin) readily bind to the DBP[3,6] (Fig. 1b, c). In contrast, bulky high molecular weight (HMW) drugs with HPSA, like ansamycins (e. g. rifampicin[5], rifabutin, and 3-formylrifamycin SV) and macrolides (e.g. erythromycin[15]) bind to the AP (Fig. 1b, c and Supplementary Fig 2). However, the mode of binding of other drugs like DDM, fusidic acid, and β-lactams by AcrB was still elusive. Until recently, two substrate sequestration routes starting from the AcrB TMD have been discovered through asymmetric crystal structures of AcrB in the presence of DDM and/or fusidic acid, where these molecules bind to the TM7/TM8 groove and/or TM1/TM2 groove, respectively[6,12,13]. Here, supported by crystal structures and functional studies, we show that the CH1-derived TM8/PC2 pathway is one of the initial substrate uptake and transport pathway for drugs with low molecular weight and low polar surface area (LMW/LPSA), including fusidic acid (LMW and LPSA) but also novobiocin (HMW and HPSA) and DDM (LMW and HPSA) (Fig. 1b and Fig. 3). Initially, these drugs are captured by the TM7/TM8 groove in the ligand-bound L protomer state[6] (Fig. 3, L^{LIG1}). The ligand subsequently moves into the entrance of CH1[15] (Fig. 3, $L1^{LIG}$) and finally unlocks the TM8/PC2 pathway where the ligand is bound proximal to the AP and DBP near the switch loop. In this conformation, the c-loop adjusts its conformation substantially to stabilize the ligand binding (this work, Fig. 3, $L2^{LIG}$). From DDM co-structures it appears that DDM can occupy either the AP[6] (Fig. 3, $T^{LIG\text{-}AP}$) or the DBP (Fig. 3, $T^{LIG\text{-}DBP}$)[16] in the T protomer. The L to T transition might be facilitated by the conformational state of the neighbouring protomer, most likely is the T state stabilized by a neighbouring protomer in either the T or O state[17,18]. The T state facilitates proton binding in the TMD, which triggers the conformational state to the O state, leading to the closure of the AP and DBP and opening of the exit

channel (Supplementary Fig 5) and efflux of the drug (Fig. 3, O). Since drug co-structures also revealed binding of drugs to the T protomer in the upper (Fig. 3, T^{LIG1})[6] or lower (T^{LIG2}) part of the TM7/TM8 groove, we anticipate a similar drug transport as mediated by the TM8/PC2 pathway (CH1) to the AP or DBP of the T protomer. Stabilization of the T state by drug binding facilitates proton binding and transition to the O state as described above. Since transport by CH1 (this work), CH3[19] and CH4[12] have been now initially described, we continued to study the transport of drugs via CH2 (Supplementary Fig 5a), which has been shown to accommodate high molecular weight (HMW) drugs in dependence of the conformation of the highly flexible switch loop [5,6].

Supplementary Note 2. Drug binding and transport of HMW drugs and the role of the switch loop

Supplementary Note 2.1 HMW drug binding and the role of the switch loop

The entrance of channel 2 (CH2) is located between the PC1 and PC2 subdomains (Fig. 1c and Supplementary Fig 5a) and contains a large pocket area for binding of HMW drugs[5,15] or also for drugs which tend to dimerize as has been shown for doxorubicin[6]. To accommodate these drugs, a flexible loop located between the AP and DBP of the L and T protomers (Fig. 1c) adopts its conformation in dependence of the physicochemical features of the drugs transported (e.g. ansamycins or macrolides). The switch loop is flexible because it contains 4 glycine residues (Gly614, Gly616, Gly619 and Gly621) and mutational analysis (Gly to Pro substitutions) has shown that this flexibility is important for transport[5,20]. Gly614Pro and Gly616Pro variants are in general inactive (Supplementary Data 2a, b; Supplementary Fig 7d, e), whereas Gly619Pro and Gly621Pro show either full or for some drugs up to 50% of the wildtype AcrB activity[20] (Supplementary Data 2a, b; Supplementary Fig 7d, e). The different substitutions cause structural changes of the switch loop in each of the AcrB protomers which are apparent in direct comparison with the conformation of the

switch loop conformation of wildtype AcrB[20] (Supplementary Fig 3a-c). Since the wildtype switch loop is expected to be highly flexible and can adopt many different conformations (and therefore is able to adopt many different drugs), substitution of Gly by Pro on one or more positions in the switch loop forces the loop to adopt only a fraction of the possible conformations. This leads to either inactivity (Gly614Pro, Gly616Pro) or a mixed fully active or reduced transport activity in dependence of the drug transported[20] (Supplementary Data 2a, b; Supplementary Fig 7d, e). To understand the transport mechanism of HMW drugs (i. e. ansamycins and erythromycin) and the role of the switch loop, we crystallized either AcrB-WT or AcrB switch loop variants in the presence of various ansamycins or erythromycin. The overall structures of the L, T, and O protomers of the Gly to Pro variants remain congruent to wildtype AcrB (Supplementary Table 1a), even though remarkable conformational changes of the switch loop are observed (Supplementary Fig 3a-c). For the AcrB wildtype and switch loop variants Gly619Pro and Gly619Pro_Gly621Pro), we readily obtained structures with 3-formylrifamycin SV bound to the AP of the L protomer (Fig. 4a and Supplementary Fig 2a-c). This observation is in line with previous HMW drug AcrB wildtype co-structures[5]. We assume that the orientation of the switch loop in wildtype AcrB is optimal for binding in the L protomer but preventing stable interactions with the HMW drugs in the AP of the T protomer. The structures of the Gly619Pro and Gly619Pro_Gly621Pro variants, however, showed in addition to the binding of 3-formylrifamycin SV in the AP of the L protomer, also simultaneous binding to the AP region of the T protomer in the same AcrB trimer (Supplementary Fig 2d, e). Binding of the HMW drugs to the T protomer AP in these switch loop variants appear to be possible due to the different switch loop orientations compared to the wildtype switch loop orientation (Supplementary Fig 3a-c). Of note, the switch loop variants, Gly619Pro and Gly619Pro_Gly621Pro, retain full to 66% of the wildtype activity for the co-crystallized substrate, 3-formylrifamycin SV (Supplementary Data 2a; Supplementary Fig 7d). The hypothesis we make for the *in vivo* situation is that HMW drugs bind at the AP in the L protomer and during the L to T transition cause an induced fit conformation of the switch loop similar to the variant (Gly619Pro and Gly619Pro_Gly621Pro) switch loop conformation

(Fig. 5 and Supplementary Fig 3d-f, h). Another switch loop variant, Gly616Pro, confers no resistance towards almost any drug tested[20] (Supplementary Data 2a, b; Supplementary Fig 7d, e), but exceptionally retains 37% of the AcrB wildtype activity against 3-formylrifamycin SV (Supplementary Data 2a; Supplementary Fig 7d). Co-crystallization of this variant in presence of erythromycin and 3-formylrifamycin SV resulted in a structure with erythromycin bound to the AP of the L protomer and simultaneous binding of 3-formylrifamycin SV to the AP of the T protomer in the same trimer (Supplementary Fig 2f, l, m). In contrast to the transport compromised Gly616Pro variant, the Gly621Pro variant shows wildtype activity towards all macrolide drugs (Supplementary Data 2b; Supplementary Fig 7e), and also toward rifabutin and rifamycin SV (Supplementary Data 2a; Supplementary Fig 7d). AcrB_Gly621Pro rifabutin co-crystals showed binding of rifabutin to the AP of T protomer but not to the AP of the L protomer (Fig. 4b and Supplementary Fig 2g). In fact, we identified binding of two molecules of rifabutin (RFB1 and RFB2) in this area, similar to the mode of binding for the doxorubicin dimer binding at the AP of the AcrB wildtype L protomer[6]. Interestingly, the switch loop conformation in the Gly621Pro variant is only slightly shifted towards the DBP area, facilitating the entry of RFB1 into the DBP area (Fig. 4b). RFB2 interacts via its naphthohydroquinone moiety with RFB1, so that it appears likely that RFB2 binding is only possible when RFB1 has bound. The conferred resistance of both the Gly619Pro and Gly621Pro variants toward most of the ansamycins (including the co-crystallized 3-formylrifamycin SV and rifabutin) and macrolides was supported by their co-complex formation with minocycline congruent to the binding of minocycline to the DBP of the wildtype T protomer (Supplementary Fig 2h, i).

No minocycline binding is observed for the mostly inactive AcrB-Gly616Pro and AcrB-Gly619Pro_Gly621Pro variants[20], despite identical co-crystallization conditions were applied for all Gly to Pro variants. The switch-loop of the AcrB-Gly619Pro_Gly621Pro double substitution variant is positioned proximal to the DBP in all three protomers and might restrict the expansion of the DBP volume to accommodate the drug (minocycline) or prevent drug transport from the AP to DBP in T

protomer (Supplementary Fig 3a-c; green). In contrast, the switch loop of Gly616Pro in the L and O protomers adopt a different conformation in comparison to wildtype AcrB and other AcrB variants, in which the switch loop is lifted and positioned toward the funnel domain (Supplementary Fig 3a-c; orange). Interestingly, the switch loop in the Gly616Pro T protomer adopts a more wildtype conformation (Supplementary Fig 3b; orange). From earlier results[20], it was shown that the removal of Phe615 and Phe617 recovers the activity of the Gly616Pro variant suggesting that the conformational restraints caused by Pro616 interfere with the transport of drugs towards the DBP by these side chains. Interestingly, the guanidinium moiety of Arg620 in the mostly inactive variants (AcrB-Gly616Pro and AcrB-Gly619Pro_Gly621Pro) is positioned towards the funnel domain and the AP (Supplementary Fig 3a-c; Gly616Pro, orange; Gly619Pro_Gly621Pro, green), respectively, whereas Arg620 of wildtype AcrB and the partially active variants is positioned towards DBP (Supplementary Fig 3a-c; wildtype, white; Gly619Pro, magenta; Gly621Pro, cyan). We speculate that Arg620, based on the large increase of the observed ansamycin resistance upon Ala-substitution (Supplementary Data 2a; Supplementary Fig 7d), might act as a side chain barrier between the AP and DBP. Still, we observed fusidic acid binding to the TM1/TM2 groove of the T protomer of the Gly619Pro_Gly621Pro variant indicating its ability to accommodate drugs at other entry sites (Supplementary Fig 2j, k). The detailed binding properties and the residues involved were analyzed in detail and used to conduct functional analysis by substitution of the side chains involved in ansamycin and macrolide binding.

Supplementary Note 2.2 Ansamycin binding to the AP is facilitated mainly by hydrophobic and π-stacking interactions

As described above, we assigned two molecules of rifabutin to the two unaccounted non-proteinaceous electron densities located at the AP of T protomer of AcrB-Gly621Pro (Fig. 4b and Supplementary Fig 2g). Both rifabutin molecules stack by their naphthohydroquinone moieties and

their ansa poly-hydroxylated chains are either extending towards the periplasmic side (for RFB2) or past the switch loop towards the DBP (for RFB1). Interaction of both rifabutin molecules inside the AP of the T protomer is mainly contributed by van der Waals and π-stacking interactions, mostly by phenylalanine and methionine side chains. Interestingly, the large portion of ansa poly-hydroxylated chain moiety of RFB1 slides underneath the switch loop towards the DBP and interacts with Phe136, Met573, Phe617, and Val67 (Fig. 4b). Moreover, Leu674 interacts with the methylpropyl-piperidine moiety of RFB1. In contrast to RFB1, RFB2 is interacting mainly with residues located in the AP. Leu828 and Phe664 interact with the ansa chain and the naphthohydroquinone moiety of RFB2, respectively. Also, Phe666 and Met575 interact with this naphthohydroquinone moiety of RFB2, but also with the one from RFB1. Only three residues form H-bonds to RFB1 and RFB2, namely Asn830 interacts with carboxylate ester of RFB1 ansa chain, Thr676 interacts with OH group of RFB2 ansa chain and Asn719 interacts with the imidazole moiety of naphthohydroquinone and the CO moiety of ansa chain of RFB2. As water molecules are poorly defined in the electron density at current resolution, we cannot rule out more H-bonding interactions between rifabutin and AcrB involving water molecules.

Binding of 3-formylrifamycin SV to wildtype AcrB is likewise mainly contributed by hydrophobic and π-stacking interactions (Supplementary Fig 2a). The ansa poly-hydroxylated chain of 3-formylrifamycin SV interacts with Phe664, Phe666, and Leu828, whereas the naphthofuran moiety interacts with Met573, Met575, Phe617, Leu668, and Arg815. Akin to the binding of rifabutin in the AcrB-Gly621Pro variant described above (Fig. 4b), only three residues (Asn719, Arg717, and Arg620) are involved in H-bond interaction with 3-formylrifamycin SV. N_{η}^{1} and N_{ε} of Arg717 interact with the C35-O moiety of the carboxylate ester and the C23-OH moiety located on the poly-hydroxylated chain of 3-formylrifamycin SV, respectively. N_{δ} and O_{δ} of Asn719 interact with the C1-OH moiety located on the 3-formylrifamycin SV naphthofuran moiety, or ansa amide/C21-OH moiety, respectively. Additionally, Arg620 in the AcrB-Gly619Pro and AcrB-Gly619Pro_Gly621Pro variants

forms a π-stacking interaction and a H-bond with the naphthofuran and amide moiety of 3-formylrifamycin SV, respectively (Supplementary Fig 2b, c). This interaction is not observed in wildtype AcrB, as the Arg620 side chain is oriented to the opposite direction, pointing towards DBP (therefore not indicated as interacting side chain in Supplementary Fig 2a. The AP comprises various hydrophilic and charged residues, such as Gln577, Asp681, Thr676, Ser715, Asn719, Glu826, Arg815, and Gln830. These residues are located in proximity to 3-formylrifamycin SV and rifabutin, but not in direct H-bonding contact. The resolution of the 3-formylrifamycin SV bound AcrB-WT structure allowed the identification of water molecules, which are mediating H-bonding interactions of Asp681, Glu826, Arg815, and Glu817 with the C8-OH of the naphthofuran moiety of 3-formylrifamycin SV (Supplementary Fig 2a). To evaluate the importance of the residues at and near the ansamycin binding sites, we used the structural insights to create single-alanine substitution variants and tested the activity of these variants via ansamycin susceptibility assays.

Supplementary Note 2.3 Both hydrophobic and hydrophilic side chains play a role in the ansamycin resistance phenotype

We started to investigate the role of bulky residues shown to be in contact or near the ansamycin binding sites. Alanine substitution of Phe136, Phe615 or Phe617 causes significantly increased susceptibility against ansamycins except for Phe617Ala for 3-formylrifamycin SV (Supplementary Data 2a; Supplementary Fig 7d). These three residues appear to be part of a gate from the AP to the DBP. Met575, Phe664, and Phe666 are bundled and close together at the PC1 subdomain site and single Ala-substitution caused mixed phenotypes for the ansamycins tested (Supplementary Data 2a; Supplementary Fig 7d). The largest effects were seen for rifabutin susceptibility, possibly because rifabutin needs accommodation of two molecules inside the AP (Fig. 4b). We also observed variants which cause increased resistance upon Ala-substitution. Leu668, Val672, Leu674, or Leu828 (except for wildtype activity of Leu668Ala against rifabutin) (Supplementary Data 2a; Supplementary Fig

7d). Val672Ala shows also with other, non-ansamycin drugs a better than wildtype phenotype, whereas Leu674Ala shows compromised activities against phenicols, β-lactams and novobiocin (Supplementary Fig 2b). These residues all reside in the c-loop, which is subject to larger conformational changes during conformational cycling (and shows conformational flexibility in the L state[21], Fig. 2b, c). Possibly, the removal of the larger hydrophobic side chains facilitates main chain hydrogen bonding during transport with ansamycins. For Leu828, located within a β-sheet and surrounded by hydrophilic side chains (e. g. Arg717, Asn719, and Glu826, Supplementary Data 2a; Supplementary Fig 7d), the nearby C23-OH and C21-OH moieties of 3-formylrifamycin SV, and the C15-O moiety of rifabutin might profit from the removal of the Leu side chain and allow for water molecules to extent the hydrogen bonding network. This might favourably stabilize the ansamycin binding, resulting in the increase of resistance against ansamycins. Based on the comparable effects of Ala-substitutions on resistance between the ansamycins, we speculate that binding of rifamycin SV and rifaximin is similar to the binding of 3-formylrifamycin SV and rifabutin, respectively.

Next, we investigated the role of polar and charged residues near the ansamycin binding sites (e.g. Gln577, Asp681, Thr676, Ser715, Arg717, Asn719, Glu826, Arg815, and Gln830). The results of the susceptibility test, however, show mixed and diverse phenotypes (Supplementary Data 2a; Supplementary Fig 7d). Some correlations between the results are apparent, however, as the alanine substitution of Arg717 causes increased resistance of *E. coli* harboring AcrB variant against those ansamycins which include an additional C-3/C-4 group (i.e. rifabutin and rifaximin), whereas more reduced resistance is observed against those without C3/C4 modification (3-formylrifamycin SV and rifamycin SV) (Supplementary Data 2a; Supplementary Fig 7d). This observation might be in line with the observed H-bond interaction between 3-formylrifamycin SV and Arg717 in co-structures of AcrB wildtype and variants in complex with 3-formylrifamycin SV (Supplementary Fig 2a) and the missing interaction between Arg717 with rifabutin in the Gly621Pro structure (Fig. 4b).

The Ala-substitution of the switch loop residue Arg620 results in one of the most gain of function towards ansamycins. Interestingly, this residue resides at the DBP region in the L protomer and therefore not involved in the binding of 3-formylrifamycin SV (Supplementary Fig 3a). However, its location changes in the T protomer, where it acts as a side chain barrier between the AP and DBP (Supplementary Fig 3b). Presumably it affects optimal transport of ansamycins upon T to O transition (Supplementary Fig 3c) and the removal of this large side chain might be favourable for ansamycin transport.

Increased susceptibility of *E. coli* is observed for cells harbouring the Glu826Ala variant against all tested ansamycins, whereas cells harboring the Asp681Ala variant shows susceptibility only against rifabutin and rifaximin (Supplementary Data 2a; Supplementary Fig 7d). This observed susceptibility might support the assumption that a water-mediated H-bond network between these residues and the ansamycins play a role in binding and/or transport of these drugs (Supplementary Fig 2a). In contrast to the effect of substitution of the hydrophobic residues located at the c-loop, alanine substitution of the hydrophilic residue Thr676 affected the cells to confer resistance against ansamycins except rifamycin SV (Supplementary Data 2a; Supplementary Fig 7d). The effect appears to be the strongest for 3-formylrifamycin SV and rifaximin, however, the wildtype co-structure does not indicate a direct interaction of this residue with 3-formylrifamycin SV (Supplementary Fig 2a). The co-structure of the rifabutin/Gly621Pro variant show direct single H-bond interaction of this residue with Thr676 (Fig. 4b) and substitution to Ala results in 30% loss of activity (Supplementary Data 2a; Supplementary Fig 7d). As we have observed in the L2 protomer and has been predicted by molecular dynamics studies[21], the c-loop most likely adopts intermediate states upon transition from the L to T state of the AcrB protomer, which might involve Thr676 as an interacting side chain with 3-formylrifamycin SV.

In contrast to the ansamycins, co-crystallization with macrolides was less yielding. We solved the structure of erythromycin bound to the AP of the L protomer of the Gly616Pro variant. Based on our insights with HMW ansamycins described above and the structural information of erythromycin-bound AcrB from this work and others[5,15], we conducted functional analysis of single-Ala substituted variants in susceptibility against 7 different macrolides.

Supplementary Note 2.4 Structural and functional analysis of macrolide binding at the AP

Congruent to erythromycin binding in the wildtype AcrB structure[5], this compound interacts in the (mostly) inactive AcrB-Gly616Pro variant by van der Waals interaction (Supplementary Fig 2l, m). It has been previously shown[20] that the Gly616Pro variant is inactive against drugs from different classes, most likely due to the to the restrained conformational flexibility inferred by the introduced proline side chain (Supplementary Fig 3 a-c, orange). However, concomitant removal of the adjacent Phe615 and Phe617 by substitution to Ala, restored the activity up to wildtype levels for most drugs, including erythromycin[20]. The main differences in the binding of erythromycin between the wildtype[5] and Gly616Pro structure (Supplementary Fig 2l, m) are contributed by side chain interactions of residues residing at the switch loop and c-loop (Fig. 1c). Val672 (c-loop), Glu673 (c-loop) and Met862 of the AcrB-Gly616Pro variant interact with the cladinose moiety of erythromycin. The macrocyclic lactone ring interacts with hydrophobic residues Phe136, Met573, and Phe615 (Supplementary Fig 2l). An increased susceptibility of cells harbouring AcrB Ala-substitution variants of Phe136, Met575, Phe615, Phe666, Glu673, Asp681, and Glu826 was observed for almost all macrolides tested (Supplementary Data 2b; Supplementary Fig 7e), indicating these residues are important in binding/transport of macrolides. As we have seen for ansamycins, we also found substitutions which resulted in variants which conferred higher resistance toward the macrolides tested. In particular, alanine substitution of Lys292, Val672, and Met862 conferred increasing resistance against almost all tested macrolides except erythromycin (Supplementary Data 2b; Supplementary Fig 7e). The

increased resistance of Lys292Ala variant might be explained by the steric hindrance caused in the wildtype AcrB due to this lysyl side chain with the 9N,11O-oxazine ring of dirithromycin, or the 8-epoxy moiety of oleandomycin, or the 9N-oxime moiety of roxithromycin. The increased resistance against tylosin and spiramycin is more difficult to interpret as these are 16-membered macrolides and the binding of these molecules might differ from the 14-membered macrolides (e.g. erythromycin) (Supplementary Data 2b; Supplementary Fig 7e).

On basis of the structural and functional insights of both ansamyin and macrolide binding and transport we suggest a possible different mode of transport for these larger (HMW) drugs, compared to the LMW drugs, which are anticipated to bind to the DBP in the T protomer.

Supplementary Note 2.5. HMW drug transport hypothesis

We propose that the transport cycle of HMW drugs is different from the low molecular weight (LMW) drugs that undergo a process of initial binding to AP and/or DBP (transport by either CH1 or CH2), or planar aromatic cationic drugs (rhodamine 6G and tetraphenylphosphonium), which prefer CH3 (Fig. 1b). The HMW drugs are recruited via CH2 (Supplementary Fig 5a) by the AP in the L protomer and subsequently, upon the transition from L to T, drugs remain bound to either the AP or the interface between the AP and DBP, below the accommodating switch loop (Fig. 5). Here, we propose two variants of HMW drug transport. First, rifampicin-type drugs (e.g. 3-formylrifamycin SV and rifampicin) are initially recruited and bound to AP in $L^{3\text{-}FOR}$ state, facilitating a conformational change of the switch loop toward the DBP (Fig. 5 and Supplementary Fig 3d, e). Upon transition of L-to-T state, the rifampicin-type drug remains at the AP in T^{LIG1} state via an induced fit mechanism causing the switch loop to remain proximal to the DBP (Fig. 5 and Supplementary Fig 3f, h). In a second possible mode of transport, macrolide/rifabutin-type drugs (e.g. erythromycin and rifabutin) are recruited and bound below switch loop in L^{ERY} state with partial penetration of drug toward the

DBP. These drugs remain at the AP in T^{LIG2} state upon transition of L to T, facilitating movement of the switch loop toward the DBP (Fig. 5 and Supplementary Fig 3g). Subsequently, a second molecule of macrolide/rifabutin-type binds to the AP in T^{LIG3} state, facilitating an induced fit PC1 subdomain movement (Fig. 5). As erythromycin has been shown to bind to the switch loop proximal region of the DBP in the *Neisseria gonorrhoeae* MtrD co-structure[22], it appears also possible that the next step would be the accommodation of one of the macrolide drugs past the switch loop in the T protomer. In the process of T to O transition, in both types of transport modes the drugs are transported directly to the funnel domain and AcrA/TolC, via TMD H^+-coupling, which induces closure of the PC1 and PC2 subdomains[7] bypassing the further DBP region, involving a peristaltic pump-like mechanism[4] (Supplementary Fig 5b).

Supplementary Note 3. Allostery induction of drug binding

Initial inspection of the initial refinement of the wildtype AcrB structure against the data from crystals grown in the presence of the antibiotic cocktail (erythromycin, linezolid, oxacillin, and fusidic acid) revealed a large displacement of TM11 and TM12 in the T protomer (Fig. 6a). This tilting creates a large cavity, open from the periplasm, and reaches into the TMD (Supplementary Fig 4b), exposing the previously assigned proton relay residues[3,4,23,24]. The C- and N-termini of TM11 and TM12 are poorly resolved, hinting at a flexible hairpin in this region, and residues 991-1003 could not be assigned. After iterative model building and multiple refinement cycles, an unaccounted non-proteinaceous electron density near the tilted TM11/TM12 was observed in the T protomer. erythromycin was ruled out from the model building as this molecule did not fit the electron density due to its bulkier macrolide ring and the two sugars side chains (Supplementary Fig 4a; Supplementary Table 1b). The electron density of the ligand is more consistent with a planar four ring structure; therefore, oxacillin and linezolid were also considered less likely binding candidates. The polder map analysis (using phenix.polder[25]) was most revealing on the best fit of the unaccounted

density (Supplementary Table 1b). The highest correlation coefficients (CC and CC_{peak}) were obtained with fusidic acid present, and the correlation coefficients were compared with those of the already confirmed (by anomalous signal) binding of minocycline[3] (PDB: 4DX5[6]) and fusidic acid[13] (PDB: 5JMN). The correlation (m1-m3) of the polder map (m3) with the synthetic map assuming fusidic acid has bound (computed using ($F_{obs} = |F_{model}|$), m1), is the highest for fusidic acid, compared to the other candidates, erythromycin, linezolid or oxacillin (Supplementary Table 1b). After several rounds of model building and refinement cycles, fusidic acid was indeed found to fit the best into the respective electron density (Fig. 6b and Supplementary Table 1b). We designated this binding site as "deep transmembrane domain binding pocket" (TMD-BP). The observed TMD-BP appears to be similar to the site where anti-tuberculosis drugs bind to the TMD of MmpL3 from *M. tuberculosis*, near to the residues involved in proton coupling[26]. Fusidic acid interacts with Leu350, Phe396, Val399, Leu400, Leu937, Phe982, and Val986 via van der Waals interactions, whereas Glu346, Thr934, and main chain of Gly930 are involve in H-bonding with fusidic acid (Fig. 6b). We probed the TMD-BP using MTS-rhodamine (MTS-R), a thiol-reactive rhodamine crosslinker[13] with the active Ala981Cys variant (Supplementary Data 2c; Supplementary Fig 7f). We observed a concentration-dependent reduction of MTS-R labelling of C981 in presence of fusidic acid, oxacillin, or novobiocin (K_i^{app} = 38, 446, or 95 µM, respectively), but linezolid and chloramphenicol could not protect against MTS-R labelling (Fig. 6c and Supplementary Fig 4f). Fusidic acid apparent K_i and its presence in the TMD-BP appears therefore to correlate. In the presence of erythromycin, a concentration-dependent increased labeling of Cys981 was observed, hinting at an erythromycin-specific TMD alteration of AcrB and thereby facilitation of MTS-R binding. Possibly, the presence of erythromycin in the crystallization mixture (containing erythromycin, linezolid, oxacillin, and fusidic acid, each at 1 mM concentration) facilitated the binding of fusidic acid, as in the presence of fusidic acid alone (at 4 to 5 mM concentration), no binding of fusidic acid at the TMD-BP can be observed.

To evaluate the functional importance of the residues lining at the TMD-BP, drug susceptibility assays were conducted with cells harbouring one of the 16 tested single alanine substitution AcrB variants against 12 different drugs, including phenicols, β-lactams, linezolid, fusidic acid, novobiocin, erythromycin, and polyaromatic cations (Supplementary Data 2c; Supplementary Fig 7f). Cells harbouring the Glu346Ala, Leu400Ala, Thr934Ala, or Leu937Ala variants showed the strongest susceptibility toward fusidic acid. Quite unexpectedly, these variants also showed increased susceptibility towards erythromycin (for all the variants tested). This result might implicate binding of erythromycin to the TMD-DP site, which we consider less likely, or an allosteric effect on the activity upon binding of erythromycin to the AP in the periplasmic porter domain. These four variants did not only confer increased susceptibility effects against fusidic acid and erythromycin, but also against all phenicols (chloramphenicol, florfenicol, thiamphenicol), β-lactams (oxacillin, dicloxacillin), linezolid and novobiocin. A general transport effect can however be excluded since doxorubicin, rhodamine 6G and tetraphenylphosphonium susceptibilities were unaffected. The only strong susceptibility against one of the polyaromatic cations was seen for the Leu400Ala variant in the presence of tetraphenylphosphonium. The Leu400Ala substitution caused complete inactivity against β-lactams and linezolid, showed residual activity against phenicols, and conferred however, still 47-66% of the wildtype resistance against fusidic acid, novobiocin and erythromycin. Glu346 and Leu350 are located on TM2 and are divergently oriented to the bound fusidic acid at the presumed allosteric binding site (Fig. 6b), so that a direct effect on drug binding to the TMD-BP does not appear likely. These residues might therefore rather be involved in the binding of the drugs to TM1/TM2 groove as a part of the CH4 transport pathway[12], rather than directly involved in the TMD-BP substrate binding. For fusidic acid and presumably also for the phenicols and β-lactams, the effects upon these Glu346Ala and Leu350Ala substitutions are suggested to be direct TM2 binding effects, as the susceptibility increase is most severe for these drugs (Supplementary Data 2c; Supplementary Fig 7f). All other substitutions are located near the TMD-BP and also close to the proton relay residues, Asp407, Asp408 and Lys940. It has been previously shown that Ala-substitution of any of the proton

relay residues results in a complete defect in transport for all drugs[27], hinting at a general H^+-coupling defect (most likely preventing the T to O transition, Supplementary Fig 5b), affecting transport of all drugs. Since the substitutions at the TMD-BP showed 100% activity toward doxorubicin, rhodamine 6G, and tetraphenylphosphonium in almost all cases, and a differentiated profile in dependence of the drug tested, we assume that these effects either interfere with direct drug binding, or cause allosteric alterations affecting binding of these drugs to their respective binding pockets at the TM1/TM2 groove, TM7/TM8 groove, the periplasmic AP or DBP pockets, or at intermediate transport sites such as the one seen for DDM in the L2 protomer.

A most interesting observation is the moderate titling of TM11/TM12 observed in the 3-formylrifamycin SV bound (to the AP) T protomer of the Gly619Pro variant (Fig. 6a). This tilting might have several effects, as e.g. the facilitation of a water network towards the titratable residues Asp407 and Asp408 and consequently facilitating the protonation of these residues resulting in the T to O transition[7]. The moderate tilting also emphasizes the previously postulated S1' site[12] near the adjacent TM2 helix (Fig. 7), where MD simulations, docking experiments, and mutagenesis experiments indicated a preference for β-lactams and fusidic acid binding. Here, we speculate that the S1' site might act as sensor site, where phenicols, β-lactams, and/or fusidic acid, when reaching a certain concentration inside the membrane, might bind to the S1' binding site in T^{LIG-TM} state (Fig. 7). This initial binding at the S1' site leads to the partially induced (PI) T state (Fig. 7, T^{PI} state), will allow subsequent recruitment of fusidic acid to the $T^{PI-LIG1}$ state, facilitating the enlargement of the TMD-BP to allow fusidic acid to slide into the TMD-BP in $T^{FI-LIG1}$ (FI for fully induced state, Fig. 7). As the fusidic acid TMD bound structure was obtained with a mixture of drugs at a rather moderate concentration (1 mM, whereas with 5 mM fusidic acid alone, no tilting can be observed[13]), it appears that fusidic acid binding to TMD-BP allosterically affects further binding of drugs. We speculate that fusidic acid bound TMD-BP induces the conformational change in the TM1/TM2 and TM7/TM8 grooves resulting in the increased binding affinity for fusidic acid in these binding sites (Fig. 1c and

2d, e, f and Supplementary Fig 2j, k). We assume that the observed allosteric binding of fusidic acid to the TMD-BP induces the slight shift of fusidic acid towards the membrane-periplasm interface at the TM1/TM2 groove (Supplementary Fig 2j), to be primed for further transport via CH4 (Fig. 7). Likewise, the binding of fusidic acid to the TM7/TM8 groove appears deeper into the groove in the fully-induced T state compared to the Phe380Ala variant fusidic acid binding (Fig. 2f). Protonation of Asp407 and Asp408 (TM4) is suggested to be highly facilitated when all the binding pockets in the TMD are occupied in $T^{FI\text{-}LIG2}$ state (Fig. 7). A previous study[7] indicated that subtle conformation changes of the TMD lead to the formation of two water channels starting from the periplasm to the protonation sites (Asp407 and Asp408), via either Glu346 (TM2) or Asp924 (TM10). We hypothesize that the proton wire is mainly located proximal to the TM2, as the TM11/TM12 groove and the TMD-BP is comprised of TM2, TM4, and TM10-TM12 (Fig. 7, $T^{FI\text{-}LIG2}$ state). In $T^{FI\text{-}LIG2\text{-}P}$ state (P for protonated, Fig. 7), the protonation of Asp407 and Asp408 facilitates the extrusion of both fusidic acid to CH1 and CH4 from their respective TM7/TM8 and TM1/TM2 groove prime sites (Fig. 1c). When the concentration of fusidic acid in the inner membrane during time are lowered as a result of AcrB pumping action, the fusidic acid at the TMD-BP is either released from the TMD-BP to the membrane or periplasm or alternatively pumped out via CH1 in $T^{FI\text{-}DR}$ state (DR for drug release, Fig. 7).

Of note, not only the TMD of the T protomer showed large conformational changes (Fig. 6a), also the periplasmic porter domain revealed conformational differences in the PC1 and PC2 subdomains in this fully induced T protomer (Supplementary Fig 4d). In particular, we observed an enlargement of the DBP volume due to a shift of serine-rich PN1-PN2 loop (residues 131-137, Fig. 1c and Supplementary Fig 4d) and the switch loop. This observation is in support to the hypothesis that fusidic acid binding to the TMD-BP could be of allosteric nature, by facilitating H^+ transfer and/or enlarging the DBP in the T protomer.

Our structural and functional results suggest that AcrB is a highly allosteric machinery. It also appears that the drug binding sites discovered thus far might be allosterically interdependent. This allostery might also affect the H^+ coupling, such to fully energize the system when drug concentrations in the inner membrane are high. Not only AcrB itself, but also the entire AcrAB-TolC system might be allosterically interdependent, as the periplasmic adaptor (AcrA) and outer membrane channel (TolC) might be tightly coupled[18,28] in the entire functional rotation cycle suggested for this kind of drug efflux machineries.

Supplementary References

1 Tseng, T. T. *et al.* The RND permease superfamily: an ancient, ubiquitous and diverse family that includes human disease and development proteins. *J Mol Microbiol Biotechnol* **1**, 107-125 (1999).

2 Elbourne, L. D., Tetu, S. G., Hassan, K. A. & Paulsen, I. T. TransportDB 2.0: a database for exploring membrane transporters in sequenced genomes from all domains of life. *Nucleic Acids Res* **45**, D320-D324, doi:10.1093/nar/gkw1068 (2017).

3 Murakami, S., Nakashima, R., Yamashita, E., Matsumoto, T. & Yamaguchi, A. Crystal structures of a multidrug transporter reveal a functionally rotating mechanism. *Nature* **443**, 173-179, doi:10.1038/nature05076 (2006).

4 Seeger, M. A. *et al.* Structural asymmetry of AcrB trimer suggests a peristaltic pump mechanism. *Science* **313**, 1295-1298, doi:10.1126/science.1131542 (2006).

5 Nakashima, R., Sakurai, K., Yamasaki, S., Nishino, K. & Yamaguchi, A. Structures of the multidrug exporter AcrB reveal a proximal multisite drug-binding pocket. *Nature* **480**, 565-569, doi:10.1038/nature10641 (2011).

6 Eicher, T. *et al.* Transport of drugs by the multidrug transporter AcrB involves an access and a deep binding pocket that are separated by a switch-loop. *Proc Natl Acad Sci U S A* **109**, 5687-5692, doi:10.1073/pnas.1114944109 (2012).

7 Eicher, T. *et al.* Coupling of remote alternating-access transport mechanisms for protons and substrates in the multidrug efflux pump AcrB. *Elife* **3**, doi:10.7554/eLife.03145 (2014).

8 Schuster, S., Vavra, M. & Kern, W. V. Evidence of a Substrate-Discriminating Entrance Channel in the Lower Porter Domain of the Multidrug Resistance Efflux Pump AcrB. *Antimicrob Agents Chemother* **60**, 4315-4323, doi:10.1128/AAC.00314-16 (2016).

9 Seeger, M. A. *et al.* The AcrB efflux pump: conformational cycling and peristalsis lead to multidrug resistance. *Curr Drug Targets* **9**, 729-749, doi:10.2174/138945008785747789 (2008).

10 Alexandrov, A. I., Mileni, M., Chien, E. Y., Hanson, M. A. & Stevens, R. C. Microscale fluorescent thermal stability assay for membrane proteins. *Structure* **16**, 351-359, doi:10.1016/j.str.2008.02.004 (2008).

11 Sjuts, H. *et al.* Molecular basis for inhibition of AcrB multidrug efflux pump by novel and powerful pyranopyridine derivatives. *Proc Natl Acad Sci U S A* **113**, 3509-3514, doi:10.1073/pnas.1602472113 (2016).

12 Tam, H. K. *et al.* Binding and Transport of Carboxylated Drugs by the Multidrug Transporter AcrB. *J Mol Biol* **432**, 861-877, doi:10.1016/j.jmb.2019.12.025 (2020).

13 Oswald, C., Tam, H. K. & Pos, K. M. Transport of lipophilic carboxylates is mediated by transmembrane helix 2 in multidrug transporter AcrB. *Nat Commun* **7**, 13819, doi:10.1038/ncomms13819 (2016).

14 Kobayashi, N., Tamura, N., van Veen, H. W., Yamaguchi, A. & Murakami, S. beta-Lactam selectivity of multidrug transporters AcrB and AcrD resides in the proximal binding pocket. *J Biol Chem* **289**, 10680-10690, doi:10.1074/jbc.M114.547794 (2014).

15 Ababou, A. & Koronakis, V. Structures of Gate Loop Variants of the AcrB Drug Efflux Pump Bound by Erythromycin Substrate. *PLoS One* **11**, e0159154, doi:10.1371/journal.pone.0159154 (2016).

16 Sennhauser, G., Bukowska, M. A., Briand, C. & Grutter, M. G. Crystal structure of the multidrug exporter MexB from Pseudomonas aeruginosa. *J Mol Biol* **389**, 134-145, doi:10.1016/j.jmb.2009.04.001 (2009).

17 Muller, R. T. & Pos, K. M. The assembly and disassembly of the AcrAB-TolC three-component multidrug efflux pump. *Biol Chem* **396**, 1083-1089, doi:10.1515/hsz-2015-0150 (2015).

18 Glavier, M. *et al.* Antibiotic export by MexB multidrug efflux transporter is allosterically controlled by a MexA-OprM chaperone-like complex. *Nat Commun* **11**, 4948, doi:10.1038/s41467-020-18770-5 (2020).

19 Zwama, M. *et al.* Multiple entry pathways within the efflux transporter AcrB contribute to multidrug recognition. *Nat Commun* **9**, 124, doi:10.1038/s41467-017-02493-1 (2018).

20 Muller, R. T. *et al.* Switch Loop Flexibility Affects Substrate Transport of the AcrB Efflux Pump. *J Mol Biol* **429**, 3863-3874, doi:10.1016/j.jmb.2017.09.018 (2017).

21 Ramaswamy, V. K., Vargiu, A. V., Malloci, G., Dreier, J. & Ruggerone, P. Molecular Rationale behind the Differential Substrate Specificity of Bacterial RND Multi-Drug Transporters. *Sci Rep* **7**, 8075, doi:10.1038/s41598-017-08747-8 (2017).

22 Lyu, M. *et al.* Cryo-EM Structures of a Gonococcal Multidrug Efflux Pump Illuminate a Mechanism of Drug Recognition and Resistance. *mBio* **11**, doi:10.1128/mBio.00996-20 (2020).

23 Murakami, S., Nakashima, R., Yamashita, E. & Yamaguchi, A. Crystal structure of bacterial multidrug efflux transporter AcrB. *Nature* **419**, 587-593, doi:10.1038/nature01050 (2002).

24 Sennhauser, G., Amstutz, P., Briand, C., Storchenegger, O. & Grutter, M. G. Drug export pathway of multidrug exporter AcrB revealed by DARPin inhibitors. *PLoS Biol* **5**, e7, doi:10.1371/journal.pbio.0050007 (2007).

25 Liebschner, D. *et al.* Polder maps: improving OMIT maps by excluding bulk solvent. *Acta Crystallogr D Struct Biol* **73**, 148-157, doi:10.1107/S2059798316018210 (2017).

26 Zhang, B. *et al.* Crystal Structures of Membrane Transporter MmpL3, an Anti-TB Drug Target. *Cell* **176**, 636-648 e613, doi:10.1016/j.cell.2019.01.003 (2019).

27 Takatsuka, Y. & Nikaido, H. Threonine-978 in the transmembrane segment of the multidrug efflux pump AcrB of Escherichia coli is crucial for drug transport as a probable component of the proton relay network. *J Bacteriol* **188**, 7284-7289, doi:10.1128/JB.00683-06 (2006).

28 Tsutsumi, K. *et al.* Structures of the wild-type MexAB-OprM tripartite pump reveal its complex formation and drug efflux mechanism. *Nat Commun* **10**, 1520, doi:10.1038/s41467-019-09463-9 (2019).

29 Wang, Z. *et al*. An allosteric transport mechanism for the AcrAB-TolC multidrug efflux pump. *Elife* **6**, doi:10.7554/eLife.24905 (2017).

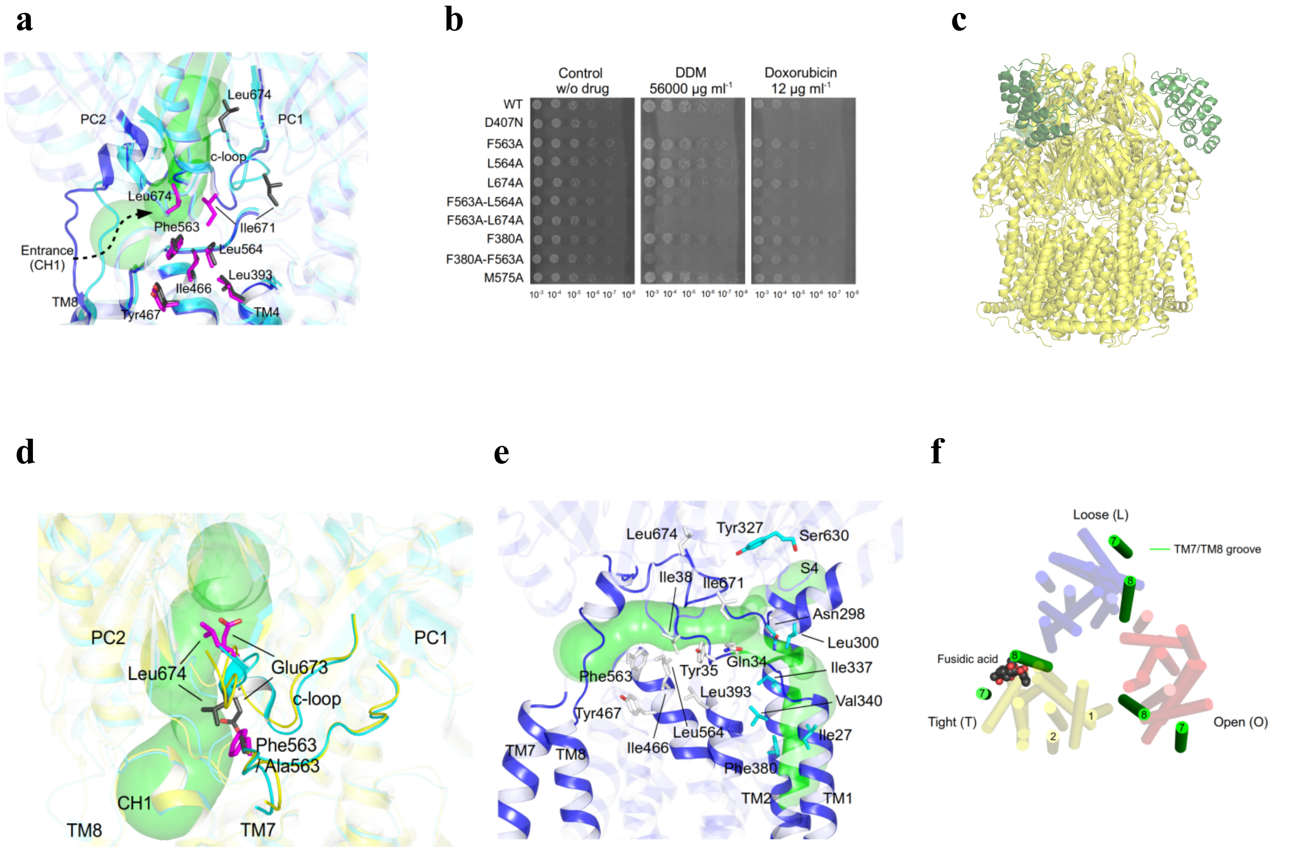

Supplementary Figure 1. Visualization of the AcrB Channel CH1-CH4. a, TM8/PC2 pathway (green) and connecting (c)-loop orientation (blue/magenta sticks, L protomer; cyan/black sticks, L2 structure). The large distance differences of residue Leu674 on the c-loop exemplifies the large conformational change. **b,** DDM and doxorubicin susceptibility profiles of AcrB variants located at the CH1 and/or CH4 pathway. $N = 4$ biologically independent replicates. **c,** Side view of the TTT Phe563Ala variant (yellow) in complex with DARPins. **d,** CH1 tunnel (green) in T protomer (PDB: 2J8S[24]) (yellow cartoon with magenta sticks) superimposed on the T protomer of Phe563Ala (cyan cartoon with black sticks). **e,** Postulated connecting tunnel (green) between CH1 (TM7/TM8, starting at Phe563) and CH4 (TM1/TM2, proceeding between Leu300 and Ile337) in the L2 protomer. Residues specifically affecting AcrB activity upon substitution are shown in white (in CH1) or cyan (in CH4) sticks. The indicated S4 binding site is part of the CH4 transport pathway[12]. **f,** Binding of fusidic acid (FUA) to the TM7/TM8 groove of AcrB-Phe380Ala T protomer.

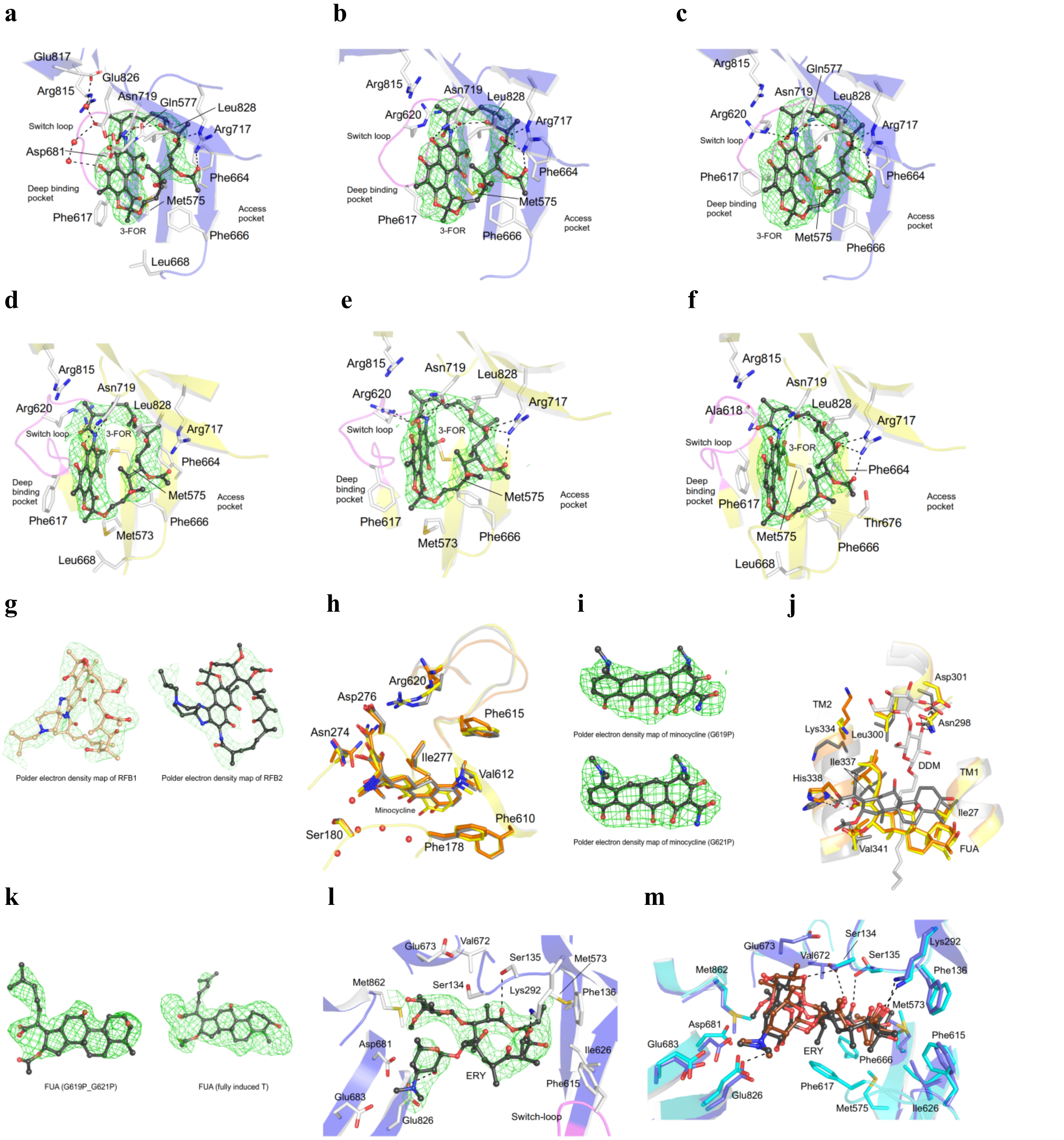

Supplementary Figure 2. Substrates binding to AcrB. Binding of 3-formylrifamycin SV (3-FOR) to the access pocket is shown for **a,** the L protomer of AcrB-WT. **b,** the L protomer of the Gly619Pro. **c,** the L protomer of the Gly619Pro_Gly621Pro. **d,** the T protomer of the Gly619Pro. **e,** the T protomer of Gly619Pro_Gly621Pro. **f,** The T protomer of the Gly616Pro. Polder electron density maps (green mesh) for 3-FOR are contoured at 4.0 *σ*. **g,** Polder electron density maps for rifabutin (RFB1 and RFB2) are contoured at 4.0 *σ*. **h,** Superimposition of wildtype AcrB (yellow), Gly619Pro (orange), and Gly621Pro (grey) with minocycline and water molecules (red spheres) bound to the deep binding pocket of the T protomer. **i,** Polder electron density maps of MIN bound to the Gly619Pro (3.8 σ, upper) or Gly621Pro (4.5 σ, lower). **j,** Superimposition of fusidic acid (FUA) bound to the T protomer TM1/TM2 groove (left) in AcrB wildtype (yellow), fully-induced T protomer (grey) and in the Gly619Pro_Gly621Pro variant. **k,** Polder electron density maps of FUA in the Gly619Pro_Gly621Pro (4.2 σ) and fully induced AcrB T protomer (4.5 σ). **l,** Binding of erythromycin (ERY) to the access pocket of Gly616Pro. The polder electron density map of ERY is contoured at 3.8 *σ*. **m,** Superimposition of ERY (carbon, black (Gly616Pro) or brown (wildtype); oxygen, red; nitrogen, blue) bound to L protomer of the Gly616Pro (blue) and AcrB wildtype (cyan), respectively.

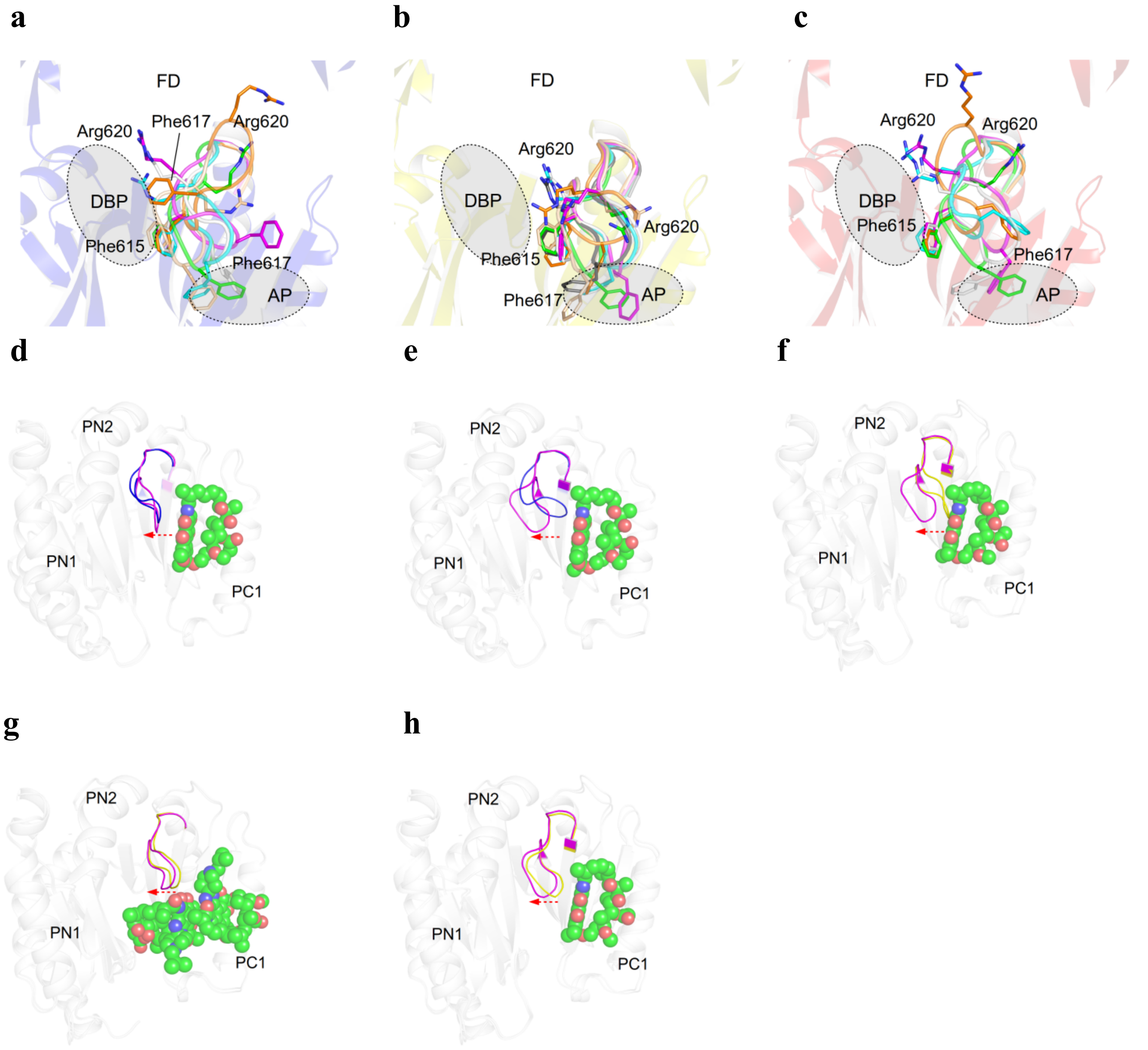

Supplementary Figure 3. Conformational states of the switch loop and induced-fit mechanism of ansamycin binding. a-c, Conformation of the switch loop in the **a,** L, **b,** T, and **c,** O protomer of apo and substrate-bound AcrB wildtype or variants. The location of the access pocket (AP) and deep binding pocket (DBP) is indicated with dashed delineated ovals. The DBP is closed in the L and O conformation and the AP is closed at the O conformation. The direction of drug transport from the AP or DBP toward the funnel domain (FD) is at the top. Color scheme: White: wildtype AcrB in complex with minocycline (MIN), orange: apo-AcrB-Gly616Pro, beige: AcrB-Gly619Pro in complex with 3-formylrifamycin SV (3-FOR), magenta: AcrB-Gly619Pro in complex with MIN, cyan: AcrB-Gly621Pro in complex with MIN, green: AcrB-Gly619Pro_Gly621Pro in complex with fusidic acid (FUA), grey: AcrB-Gly621Pro in complex with rifabutin (RFB). **d-h**, Superimposition of ansamycin-bound (switch loop in magenta) and apo (switch loop in the L protomer blue, in T yellow) AcrB variants. **d,** L protomer of 3-FOR-bound (sphere) in wildtype AcrB. **e,** L protomer of 3-FOR-bound Gly619Pro variant. **f,** T protomer of 3-FOR-bound Gly619Pro variant. **g,** T protomer of RFB-bound Gly621Pro variant. **h,** T protomer of 3-FOR-bound Gly619Pro_Gly621Pro variant. The movement of switch loop towards the DBP is depicted as red arrow. For orientation, the PN1, PN2, and PC1 subdomains (transparent grey cartoon) are indicated.

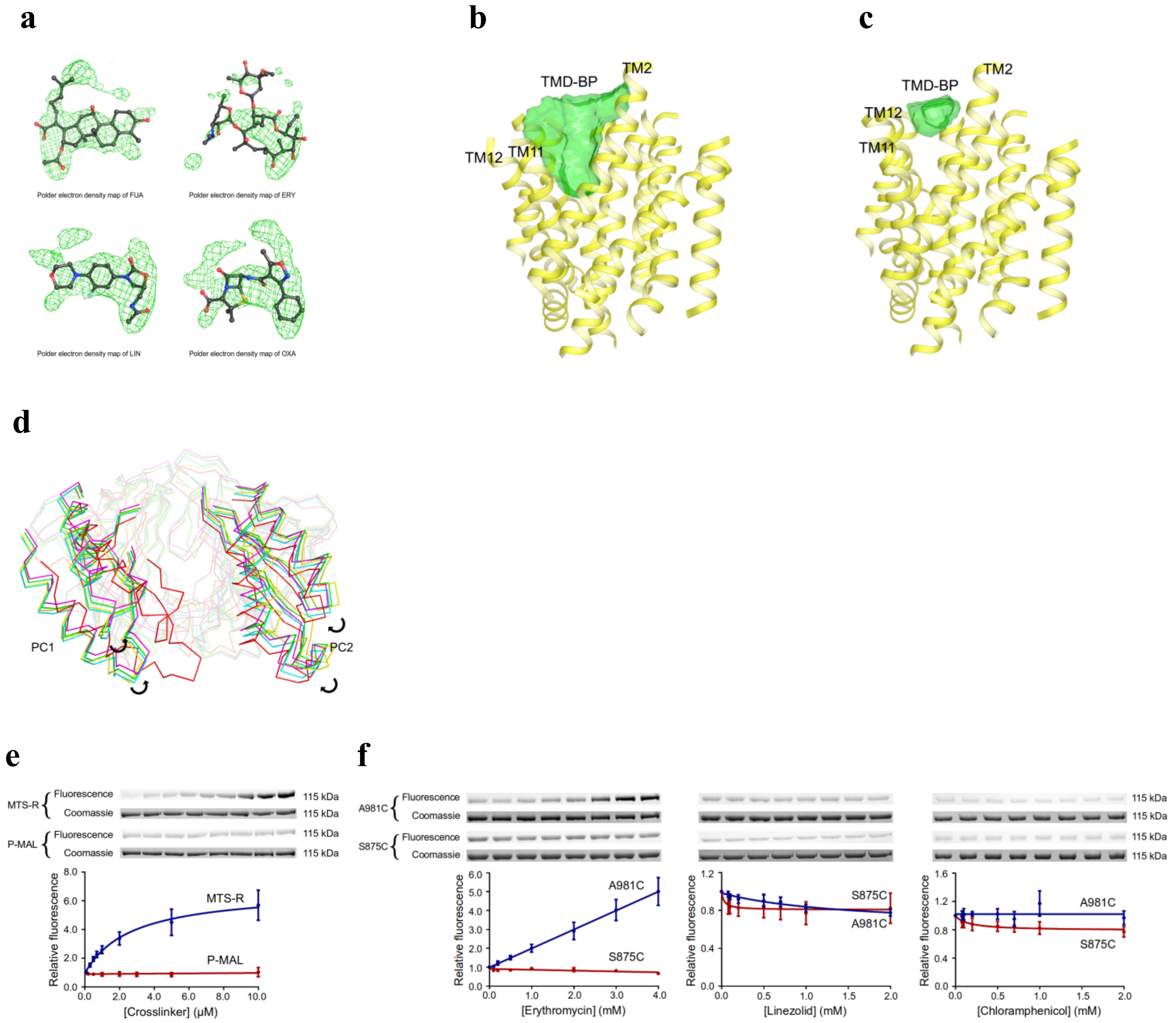

Supplementary Figure 4. Deep transmembrane domain binding pocket (TMD-BP) and TM7/TM8 groove of the fully induced T protomer. a, Polder maps (green mesh) of erythromycin (ERY), fusidic acid (FUA), linezolid (LIN), and oxacillin (OXA) as modelled in TM11/TM12 groove are contoured at 5.0 σ except for FUA (4.5 σ) (carbon, black; oxygen, red; nitrogen, blue; sulfur, yellow; fluorine, light blue). For further discussion, refer to Supplementary Note 3. **b-c,** Cavities of the TMD-BP in **b,** major cavity (1700 $Å^3$) of the fully induced T protomer. **c,** minor cavity (304 $Å^3$) of the partially induced T protomer in L2-T-O structure. **d,** Conformational changes of the T protomer porter domain of AcrB (yellow), partially induced AcrB (cyan), partially induced G619P variant (green), AcrB $T^{FI\text{-}LIG2}$ (fully induced, magenta) and of the AcrB O protomer (red). **e,** Determination of the MTS-rhodamine (MTS-R) labelling efficiency of AcrB_Cys981 (apparent K_d = 2.68 µM). No covalent labelling for *N*-(1-pyrenyl) maleimide (P-MAL). Data are presented as mean ± s.e.m. of N = 3-4 independent experiments (Source Data Supplementary Fig 4e). **f,** Substrate protection experiment of AcrB-cl_Cys981 and AcrB-cl_Cys875 by in-gel fluorescence of MTS-rhodamine modified proteins in dependence of the drug concentration (from left to right increasing concentration of drugs). Data are presented as mean ± s.e.m. of N = 4 independent experiments (Source Data Supplementary Fig 4f).

a

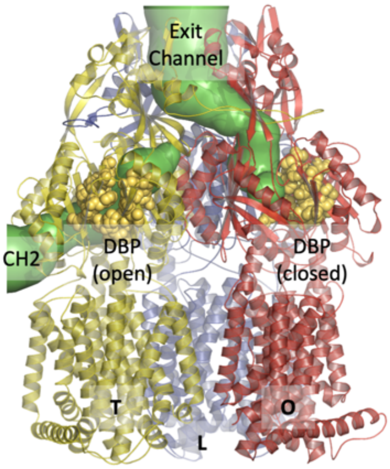

b

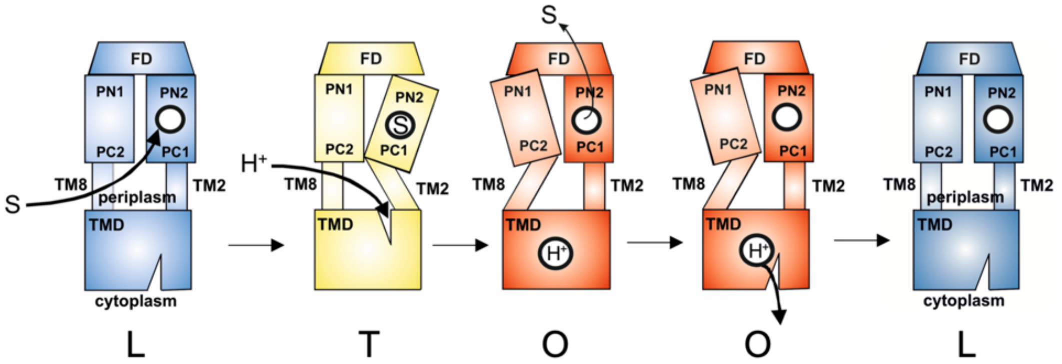

Supplementary Figure 5. Functional rotation cycle of AcrB H^+/drug antiport. a, Side view of the AcrB trimer (cartoon) with the three protomers L, T, and O in blue, yellow, and red, respectively. In the periplasmic part of AcrB (comprising the subdomains PN1, PN2, PC1, PC2 and the funnel domain (FD)), the deep binding pocket (DBP, yellow sphere) is open in the T and closed in the O conformation. Channel 2 (CH2, green surface) is present in the T protomer (yellow) with the entrance from the periplasm, is one of the four channels leading to the open deep binding pocket (CH1, CH2 and CH3 are displayed in Fig. 1b). In the O protomer (red), all four channels and the DBP are collapsed and not present, whereas an exit channel (green surface) is available for drugs to be transported towards the AcrA/TolC tunnel (Fig. 1a) from the closed DBP. **b,** Functional rotation cycle of the AcrB protomer. Every protomer in the AcrB trimer can adopt any of these states in a consecutive fashion. The cycle starts with substrate (S) binding to the L protomer (or state, blue) via one of the channels CH1-CH4. The T protomer (yellow) binds the drug in the DBP and triggers a signal transduced by transmembrane helix 2 (TM2) to the transmembrane domain (TMD). This opens a water-mediated proton (H^+) channel towards the proton relay residues (Asp407, Asp408) deeply inside the TMD. Protonation of these residues triggers the T to O transition, which starts with a conformational change in the TMD due to the altered electrostatics, which is transduced via TM8 to the periplasmic part and triggers the collapse of all entrance channels CH1-CH4 and the DBP. This leads to extrusion of drug from the now closed DBP via the exit channel (shown in **a**)). Release of the drug triggers the O to L transition (via the PN1/PC2 subdomains and TM8) with release of the proton (H^+) to the cytoplasm and return to the L state for the next round of drug uptake.

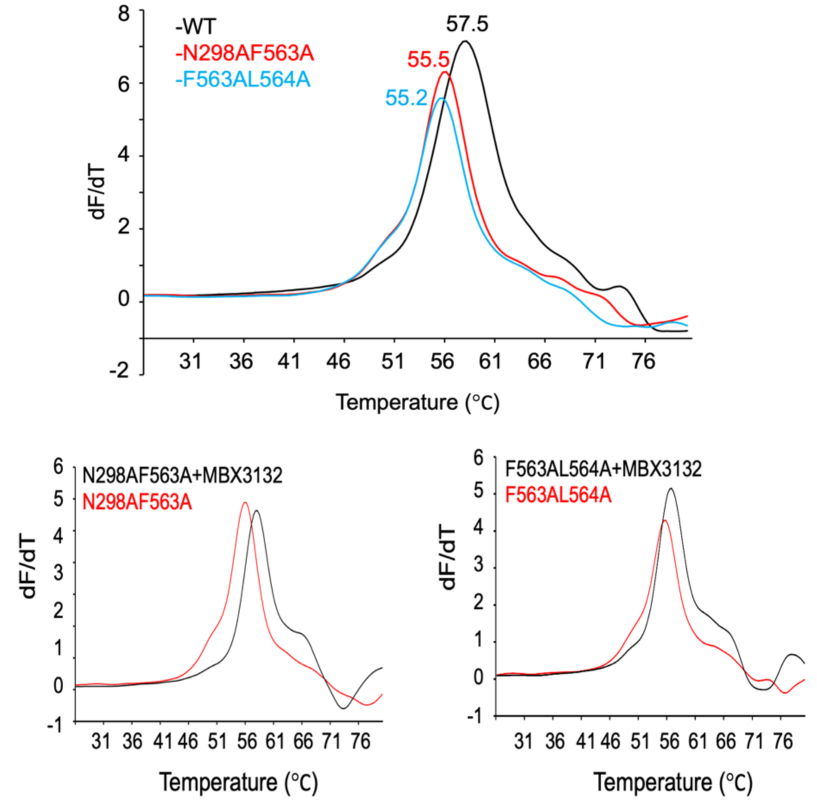

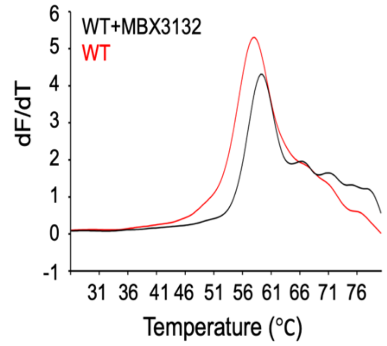

Sample	Tm
AcrB(WT)	57.5±0
AcrB(WT)+MBX	**58.6**±0.17
N298AF563A	55.4±0.17
N298AF563A+MBX	**57.3**±0.17
F563AL564A	55.2±0
F563AL564A+MBX	**56.2**±0

Supplementary Figure 6. Thermal shift assay with purified AcrB wildtype and the Asn298Ala_Phe563Ala and Phe563Ala_Leu564Ala double-substitution variants. a, Both variants have a slightly lower melting temperature compared to the AcrB wildtype (ΔTm = 2°C). However, as shown under b, both variants are thermally stabilized by the addition of the inhibitor MBX3132 (ΔTm (+/- MBX3132) = 1-2°C) indicating binding of the inhibitor and thermal stabilization of the AcrB/inhibitor complex. This indicates that the variants are structurally comparable to the wildtype and correctly folded. MBX3132 binding to the DBP of AcrB has been shown via crystal structures by Sjuts et al.[11] and via single-particle Cryo-EM by Wang et al.[29]

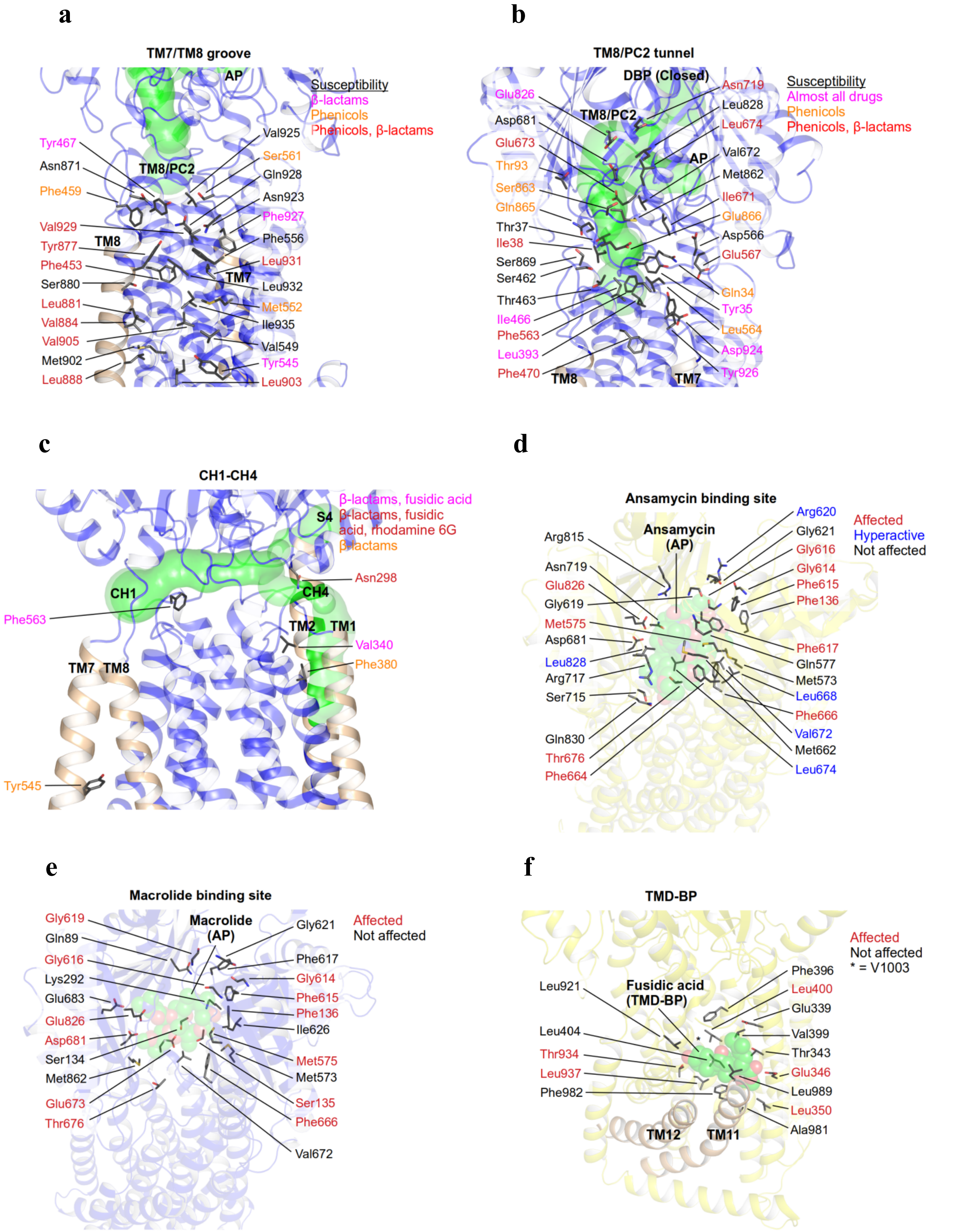

Supplementary Figure 7. Location of amino acids specifically involved in the transport pathway. a, TM7/TM8 groove, b, TM8/PC2 tunnel, c, CH1/CH4 tunnels, d, ansamycins, e, macrolides, f, TMD-BP pathway. These amino acids were substituted to alanine for the phenotypic analysis as described in Supplementary Data 1 and 2.

Supplementary Table 1. Superimposition of the AcrB structures from this study with AcrB wildtype and local correlation coefficients (CC) and CC_{peak} as calculated by phenix.polder[25]. A, superimposition of individual L, T, or O protomers from AcrB variants with minocycline bound wildtype AcrB (PDB ID: 4DX5[6]). **b,** The calculated CC and CC_{peak} by phenix.polder for fusidic acid bound to TM1/TM2 (PDB: 5JMN[13]) and minocycline bound to deep binding pocket (PDB: 4DX5[6]) are shown as reference of the substrate binding. Erythromycin, linezolid, and oxacillin were modelled to the electron density map of the TMD-BP in fully induced T protomer, and these models were calculated for the Polder map (Supplementary Fig 4a).

a

**AcrB structures**	**Minocycline bound AcrB (RMSD of Cα)**		
	**L**	**T**	**O**
Fusidic acid bound to TMD-BP of AcrB fully induced T protomer	0.41 Å	**1.62 Å**	0.59 Å
DDM bound to TM8/PC2 tunnel of AcrB L2 protomer	**1.07 Å**	**1.12 Å**	0.38 Å
3-Formylrifamycin SV bound AcrB	0.43 Å	**0.70 Å**	0.34 Å
3-Formylrifamycin SV and erythromycin bound AcrB-Gly616Pro	**0.58 Å**	0.55 Å	0.54 Å
3-Formylrifamycin SV bound AcrB-Gly619Pro	0.47 Å	**0.86 Å**	0.45 Å
Minocycline bound AcrB-Gly619Pro	0.36 Å	0.34 Å	0.33 Å
Minocycline bound AcrB-Gly621Pro	0.35 Å	0.32 Å	0.40 Å
Rifabutin bound AcrB-Gly621Pro	0.44 Å	0.55 Å	0.45 Å
Fusidic acid bound AcrB-Gly619Pro_Gly621Pro	0.36 Å	0.46 Å	0.49 Å
3-Formylrifamycin SV bound AcrB-Gly619Pro_Gly621Pro	0.39 Å	**0.64 Å**	0.52 Å
Minocycline bound AcrB-Ile38Phe_Ile671Thr	0.36 Å	0.34 Å	0.31 Å
Fusidic acid bound AcrB-Phe380Ala	0.40 Å	0.36 Å	0.27 Å
AcrB-Phe563Ala (TTT conformation)	-	**1.19 Å**	-

b

**Structures**	**m1-m2**		**m1-m3**		**m2-m3**	
	CC	CC_{peak}	CC	CC_{peak}	CC	CC_{peak}
Fusidic acid bound to AcrB (5JMN)[18]	0.23	0.24	0.75	0.72	0.38	0.40
Minocycline bound to AcrB (4DX5)[6]	0.55	0.60	0.89	0.85	0.59	0.62
Fusidic acid bound to TMD-BP	0.60	0.60	0.82	0.79	0.68	0.70
Erythromycin modelled to TMD-BP	0.51	0.57	0.64	0.69	0.85	0.84
Linezolid modelled to TMD-BP	0.51	0.57	0.70	0.73	0.78	0.74
Oxacillin modelled to TMD-BP	0.52	0.58	0.65	0.69	0.81	0.80

Supplementary Table 2. Primer sequences (PHO: phosphorylated at 5' end).

**Plasmid**	**Primer sequences**
pET24acrB$_{His}$	5'-GGATCCCATATGCCTAATTTCTTTATCGATC-3'<br>5'-AAGCTTCTCGAGATGATGATCGACAGTATGGCTG-3'
pET24acrB-Q34A$_{His}$	5'-GCATATCCTACGATTGCACCG-3'<br>5'-CGCCACCGGCAGTTTGAGG-3'
pET24acrB-Y35A$_{His}$	5'-CAAGCTCCTACGATTGCACCG-3'<br>5'-CGCCACCGGCAGTTTGAGGA-3'
pET24acrB-T37A$_{His}$	5'-GCGATTGCACCGCCGGC-3'<br>5'-AGGATATTGCGCCACCGGC-3'
pET24acrB-I38A$_{His}$	5'-GCCGCACCGCCGGCAGTAAC-3'<br>5'-CGTAGGATATTGCGCCACCG-3'
pET24acrB-I38F$_{His}$	5'-TTTGCACCGCCGGCAGTAAC-3'<br>5'-CGTAGGATATTGCGCCACCG-3'
pET24acrB-Q89A$_{His}$	5'-GTGGCGATCACCCTGACCTT-3'<br>5'-GGTACCCGTGGAGTCACTG-3'
pET24acrB-T93A$_{His}$	5'-CTGGCCTTTGAGTCTGGTACT-3'<br>5'-GGTGATCTGCACGGTACCC-3'
pET24acrB-S134A$_{His}$	5'-GCCAGCTTCCTGATGGTTGTC-3'<br>5'-GGATGATTTCTCAACGCTCAC-3'
pET24acrB-S135A$_{His}$	5'-GCCTTCCTGATGGTTGTCGG-3'<br>5'-GCTGGATGATTTCTCAACGC-3'
pET24acrB-F136A$_{His}$	5'-TCCAGCAGCGCCCTGATGGTTG-3'<br>5'-GATTTCTCAACGCTCACCCCTTGC-3'
pET24acrB-K292A$_{His}$	5'-CTGGGGATCGCACTGGCGAC-3'<br>5'-ACCGGAAGCCGGTTGGCCGTTA-3'
pET24acrB-N298A$_{His}$	5'-GGCGACCGGTGCAGCGGCGCTGGATACCG-3'<br>5'-CGGTATCCAGCGCCGCTGCACCGGTCGCC-3'
pET24acrB-E339A$_{His}$	5'-CTCTATTCACGCAGTGGTTAAAAC-3'<br>5'-ATTTTCACGAACGGCGTGGTGTC-3'
pET24acrB-V340A$_{His}$	5'-CTATTCACGAAGCGGTTAAAACGC-3'<br>5'-AGATTTTCACGAACGGCGTGGTG-3'
pET24acrB-T343A$_{His}$	5'-CGCTGGTCGAAGCGATCAT-3'<br>5'-CTTTAACCACTTCGTGAATAGAGAT-3'
pET24acrB-E346A$_{His}$	5'-GCAGCGATCATCCTCGTGTTC-3'<br>5'-GACCAGCGTTTTAACCACTTC-3'
pET24acrB-L350A$_{His}$	5'-GCCGTGTTCCTGGTTATGTATCTGT-3'<br>5'-GATGATCGCTTCGACCAGC-3'
pET24acrB-F380A$_{His}$	5'-CGGGACCGCAGCCGTCC-3'<br>5'-AGCAATACCACCGGTACGGC-3'
pET24acrB-L393A$_{His}$	5'-GCCACAATGTTCGGGATGGT-3'<br>5'-LCGTGTTTATCGAGAAGCCAAAG-3'
pET24acrB-F396A$_{His}$	5'-GCTAACAATGGCCGGGATGGTGC-3'<br>5'-GTGTTTATCGAGAAGCCAAAGGC-3'
pET24acrB-L400A$_{His}$	5'-GTTGCAGCCATCGGCCTGTT-3'<br>5'-CATCCCGAACATTGTTAGCG-3'
pET24acrB-L404A$_{His}$	5'-GCATTGGTGGATGACGCCA-3'<br>5'-GCCGATGGCGAGCACCAT-3'
pET24acrB-L449A$_{His}$	5'-GCGTCGGCGGTATTCGTAC-3'<br>5'-TACCATCGCGATACCGACCA-3'
pET24acrB-F453A$_{His}$	5'-GCCGTACCGATGGCCTTCT-3'<br>5'-TACCGCCGACAGTACCATC-3'
pET24acrB-F459A$_{His}$	5'-GCAGGCGGTTCTACTGGTGC-3'

	5'-GAAGGCCATCGGTACGAATAC-3'
pET24acrB-S462A$_{His}$	5'-GCAACTGGTGCTATCTATCGTC-3'<br>5'-ACCGCCAAAGAAGGCCATC-3'
pET24acrB-T463A$_{His}$	5'-GCTGGTGCTATCTATCGTCAG-3'<br>5'-AGAACCGCCAAAGAAGGCC-3'
pET24acrB-I466A$_{His}$	5'-CTGCCTATCGTCAGTTCTCTA-3'<br>5'-CACCAGTAGAACCGCCAAAG-3'
pET24acrB-Y467A$_{His}$	5′-GGCGCCATCGCTCGTCAGTTCTCTATTACCATT-3′<br>5′-AGTAGAACCGCCAAAGAAGGC-3′
pET24acrB-F470A$_{His}$	5'-GCCTCTATTACCATTGTTTCAGC-3'<br>5'-CTGACGATAGATAGCACCAGT-3'
pET24acrB-Y545A$_{His}$	5'-GCACTGATCATCGTGGTCGGC-3'<br>5'-CAGCACCAGGTAACGCCCC-3'
pET24acrB-V549A$_{His}$	5'-GCGGTCGGCATGGCCTATC-3'<br>5'-GATGATCAGATACAGCACCAGG-3'
pET24acrB-M552A$_{His}$	5'-GCGGCCTATCTGTTCGTGCGTC-3'<br>5'-GCCGACCACGATGATCAGATAC-3'
pET24acrB-F556A$_{His}$	5'-GCCGTGCGTCTGCCAAGCTCC-3'<br>5'-CAGATAGGCCATGCCGACC-3'
pET24acrB-S561A$_{His}$	5'-CCTCCTTCTTGCCAGATGAGG-3'<br>5'-CTGGCAGACGCACGAACAGA-3'
pET24acrB-F563A$_{His}$	5'-GCATTGCCAGATGAGGACC-3'<br>5'-GGAGCTTGGCAGACGCAC-3'
pET24acrB-F563L$_{His}$	5'-TTGTTGCCAGATGAGGACC-3'<br>5'-GGAGCTTGGCAGACGCAC-3'
pET24acrB-F563W$_{His}$	5'-TGGTTGCCAGATGAGGACC-3'<br>5'-GGAGCTTGGCAGACGCAC-3'
pET24acrB-F563Y$_{His}$	5'-TACTTGCCAGATGAGGACC-3'<br>5'-GGAGCTTGGCAGACGCAC-3'
pET24acrB-L564A$_{His}$	5'-CTTCGCGCCAGATGAGGACCAG-3'<br>5'-GAGCTTGGCAGACGCACGA-3'
pET24acrB-D566A$_{His}$	CAGAGGACCAGGGCGTGTT-3'<br>CTGGCAAGAAGGAGCTTGGC-3'
pET24acrB-E567A$_{His}$	5'-CAGATGCGGACCAGGGCGTG-3'<br>5'-GCAAGAAGGAGCTTGGCAGAC-3'
pET24acrB-M573A$_{His}$	5'-GCGTGTTTGCAACCATGGTTC-3'<br>5'-CCTGGTCCTCATCTGGCAAG-3'
pET24acrB-M575A$_{His}$	5'-GTTTATGACCGCGGTTCAGCTGC-3'<br>5'-ACGCCCTGGTCCTCATCTGGC-3'
pET24acrB-Q577A$_{His}$	5'-TGCGCTGCCAGCAGGTGCAA-3'<br>5'-ACCATGGTCATAAACACGCCC-3'
pET24acrB-G614P$_{His}$	5'-GTTCGCCGTTAACCCGTTCGGCTTTGC-3'<br>5'-ACCGACTCAACGTTGTTCTTTTCTTTG-3'
pET24acrB-F615A$_{His}$	5′-AACGGCGCCGGCTTTGCGGGTCGTGGTCAGAATACCGGTATTGCGTTCGTA-3′<br>5′-AGCTTACGAACGCAATACCGGTATTCTGACCACGACCCGCAAAGCCGGCGCCGTT-3′<br>Müller et al., 2017[20]
pET24acrB-G616P$_{His}$	5'-GTTAACGGCTTCCCGTTTGCGGGACG-3'<br>5'-GGCGAACACCGACTCAACGTTG-3'
pET24acrB-F617A$_{His}$	5′-AACGGCTTCGGCGCTGCGGGTCGTGGTCAGAATACCGGTATTGCGTTCGTA-3′<br>5′-AGCTTACGAACGCAATACCGGTATTCTGACCACGACCCGCAGCGCCGAAGCCGTT -3′<br>Müller et al., 2017[20]
pET24acrB-G619P$_{His}$	5'-CGGCTTTGCGCCACGTGGTCAGAATAC-3'<br>5'-AAGCCGTTAACGGCGAACACCGAC-3'
pET24acrB-R620A$_{His}$	5'-CTTTGCGGGAGCAGGTCAGAATAC-3'<br>5'-CCGAAGCCGTTAACGGCGAACAC-3'

pET24acrB-G621P$_{His}$	5’-CTTTGCGGGACGTCCTCAGAATACCGG-3’<br>5’-CCGAAGCCGTTAACGGCGAACAC-3’
pET24acrB-I626P$_{His}$	5’-CAGCGTTCGTTTCCTTGAAGG-3’<br>5’-CACCGGTATTCTGACCACGTC-3’
pET24acrB-M662A$_{His}$	5’-GCAGTTTTCGCCTTTAACCTG-3’<br>5’-CGCATCTTTGATTTGCGAGAAAG-3’
pET24acrB-F664A$_{His}$	5’-GATGGTTGCCGCCTTTAAC-3’<br>5’-GCATCTTTGATTTGCGAGAAAG-3’
pET24acrB-F666A$_{His}$	5’-GGTTTTCGCCGCAAACCTGCCCGC-3’<br>5’-ATCGCATCTTTGATTTGCGAGAAAG-3’
pET24acrB-L668A$_{His}$	5’-GCGCCCGCAATCGTGGAACTG-3’<br>5’-GTTAAAGGCGAAAACCATCGC-3’
pET24acrB-I671A$_{His}$	5’-GCCGTGGAACTGGGTACTG-3’<br>5’-TGCGGGCAGGTTAAAGGC-3’
pET24acrB-I671T$_{His}$	5’-ACCGTGGAACTGGGTACTG-3’<br>5’-TGCGGGCAGGTTAAAGGC-3’
pET24acrB-V672A$_{His}$	5’-GCGGAACTGGGTACTGCAAC-3’<br>5’-GATTGCGGGCAGGTTAAAG-3’
pET24acrB-E673A$_{His}$	5’-GCACTGGGTACTGCAACC-3’<br>5’-CACGATTGCGGGCAGGTTAA-3’
pET24acrB-L674A$_{His}$	5’-GCGGGTACTGCAACCGGC-3’<br>5’-TTCCACGATTGCGGGCAGG-3’
pET24acrB-T676A$_{His}$	5’-GCAGCAACCGGCTTTGACTTTG-3’<br>5’-ACCCAGTTCCACGATTGCGG-3’
pET24acrB-D681A$_{His}$	5’-GCCTTTGAGCTGATTGACCAG-3’<br>5’-AAAGCCGGTTGCAGTACCCA-3’
pET24acrB-E683A$_{His}$	5’-CGCTGATTGACCAGGCTGGC-3’<br>5’-CAAAGTCAAAGCCGGTTGCAG-3’
pET24acrB-S715A$_{His}$	5’-GCAGTACGTCCAAACGGTCT-3’<br>5’-GGTCAACATATCAGGGTGCTTC-3’
pET24acrB-R717A$_{His}$	5′-CCCTGATATGTTGACCAGCGTGGCGCCAAACGGTCTGGAAGATACCCC-3′<br>5′-GGGGTATCTTCCAGACCGTTTGGCGCCACGCTGGTCAACATATCAGGG-3′
pET24acrB-N719A$_{His}$	5’-GTACGTCCAGCCGGTCTGGAAG-3’<br>5’-GCTGGTCAACATATCAGGGTGCTTC-3’
pET24acrB-R815A$_{His}$	5’-GCACTGGAACGTTACAACGG-3’<br>5’-CGGCGAACCGTACTCCCAAC-3’
pET24acrB-E826A$_{His}$	5’-CCATCCATGGCAATCTTAGGCC-3’<br>5’-CAGGCCGTTGTAACGTTCCAGAC-3’
pET24acrB-L828A$_{His}$	5’-GCAGGCCAGGCGGCACC-3’<br>5’-GATTTCCATGGATGGCAGGCC-3’
pET24acrB-Q830A$_{His}$	5’-GCGGCGGCACCGGGTAAAAG-3’<br>5’-GCCTAAGATTTCCATGGATGGC-3’
pET24acrB-M862A$_{His}$	5’-CTGGACGGGGGCATCCTATCAGG-3’<br>5’-TCATAGCCAACACCGGTAGGCAGTT-3’
pET24acrB-S863A$_{His}$	5′-GGACGGGGATGGCCTATCAGGAGCGCTTATCCGGCAACC-3′
pET24acrB-Q865A$_{His}$	5′-GGACGGGGATGTCCTATGCGGAGCGCTTATCCGGCAACC-3′
pET24acrB-E866A$_{His}$	5′-GGATGTCCTATGCTGAGCGCTTATCCGGCAACC-3′
pET24acrB-S869A$_{His}$	5′-TGTCCTATCAGGAACGCTTAAGCGCCAACCAGGCACCTTCAC-3′
pET24acrB-N871A$_{His}$	5′-TGTCCTATCAGGAACGCTTAAGCGGCAACGCGGCACCTTCAC-3′
pET24acrB-Y877A$_{His}$	5′-GGCACCTTCACTGGCCGCGATAAGCTTGATTGTCG-3′
pET24acrB-S880A$_{His}$	5′-GTACGCGATTGCGTTGATTGTCGTGTTCCTGTGCTTAGCGGCGCTGT-3′
pET24acrB-L881A$_{His}$	5′-GTACGCGATTTCGGCGATTGTCGTGTTCCTGTGCTTAGCGGCGCTGT-3′
pET24acrB-V884A$_{His}$	5′-GTACGCGATTTCGTTGATTGTCGCGTTCCTGTGCTTAGCGGCGCTGT-3′

pET24acrB-L888A$_{His}$	5’-CGGCGGCGCTGTACGAGAGC-3’<br>5’-CACACAGGAACACGACAATCAAC-3’
pET24acrB-M902A$_{His}$	5’-CGCTGGTCGTTCCGCTGGG-3’<br>5’-CAACGGAGAACGGAATCGACC-3’
pET24acrB-L903A$_{His}$	5’-GCGGTCGTTCCGCTGGGGGTT-3’<br>5’-CATAACGGAGAACGGAATCGAC-3’
pET24acrB-V905A$_{His}$	5’-GCACCGCTGGGGGTTATCGGTG-3’<br>5’-GACCAGCATAACGGAGAACG-3’
pET24acrB-L921A$_{His}$	5’-GCGACCAATGACGTTTACTTC-3’<br>5’-GCCGCGGAAGGTGGCAGC-3’
pET24acrB-N923A$_{His}$	5’-GCAGACGTTTACTTCCAGGTAGGC-3’<br>5’-GGTCAGGCCACGGAAGGTG-3’
pET24acrB-D924A$_{His}$	5’-CCGTTTACTTCCAGGTAGGCC-3’<br>5’-CATTGGTCAGGCCACGGAAG-3’
pET24acrB-V925A$_{His}$	5’-ACGCATACTTCCAGGTAGGC-3’<br>5’-CATTGGTCAGGCCACGGAA-3’
pET24acrB-Y926A$_{His}$	5’-TTCCAGGTAGGCCTGCTCA-3’<br>5’-GGCAACGTCATTGGTCAGGC-3’
pET24acrB-F927A$_{His}$	5’-GCCCAGGTAGGCCTGCTCA-3’<br>5’-GTAAACGTCATTGGTCAGGC-3’
pET24acrB-Q928A$_{His}$	5′-GCGGTAGGCCTGCTCACAACCA-3′<br>5′-GAAGTAGACGTCATTGGTCAGGCCACG-3′
pET24acrB-V929A$_{His}$	5’-GCAGGCCTGCTCACAACCA-3’<br>5’-CTGGAAGTAAACGTCATTGGTC-3’
pET24acrB-L931A$_{His}$	5’-GCGCTCACAACCATTGGGTTGTC-3’<br>5’-GCCTACCTGGAAGTAAACGTC-3’
pET24acrB-L932A$_{His}$	5’-GTAGGCCTGGCCACAACCATTG-3’<br>5’-CTGGAAGTAAACGTCATTGGTC-3’
pET24acrB-T934A$_{His}$	5’-CCATTGGGTTGTCGGCGAAG-3’<br>5’-CTGTGAGCAGGCCTACCTGG-3’
pET24acrB-I935A$_{His}$	5’-GCAGGGTTGTCGGCGAAGAACG-3’<br>5’-GGTTGTGAGCAGGCCTACC-3’
pET24acrB-L937A$_{His}$	5’-CAACCATTGGGGCGTCGGCGAAGAAC-3’<br>5’-TGAGCAGGCCTACCTGGAAGTAAAC-3’
pET24acrB-cl_A981C$_{His}$	5’-TGCTTTATCCTCGGCGTTATGCC-3’<br>5’-CAGCGAGGTCATCAGGATC-3’
pET24acrB-F982A$_{His}$	5’-GCAATCCTCGGCGTTATGCCGC-3’<br>5’-CGCCAGCGAGGTCATCAGG-3’
pET24acrB-L989A$_{His}$	5’-GGCGGTTATCAGTACTGGTGC-3’<br>5’-GGCATAACGCCGAGGATAAA-3’
pET24acrB-V1003A$_{His}$	5’-GCAGGCACCGGTGTAATGG-3’<br>5’-TGCGTTCTGCGCGCCGGA-3’
