# Supplementary material for: Allosteric drug transport mechanism of multidrug transporter AcrB
Source: Nat Commun. 2021 Jun 29;12:3889. doi: 10.1038/s41467-021-24151-3 (PMC8242077; doi:10.1038/s41467-021-24151-3)
Supplement: Supplementary file 3 — Description of Additional Supplementary Files [file 41467_2021_24151_MOESM3_ESM.pdf]

Description of Additional Supplementary Files

File Name: Supplementary Data 1
Description: **Drug susceptibility profiles of AcrB substitution variants. a,** TM7/TM8 groove, **b,** TM8/PC2 tunnel, **c,** CH1-CH4 tunnels. Data are mean s.e.m. of N = ≥3-4 biologically independent cells. * represents p value = 0.005-0.05 (Students t-test, two-sided); ** represents p value < 0.005 (Students t-test, two-sided). Growth of the cells: red, ≤0.6 (severely affected); light red, 0.6-0.8 (affected if statistically significant, marked with * or **); lightest red, 0.8-0.9 (not affected); blue, >1.3; light blue, 1.2-1.3; lightest blue, 1.1-1.2 (refer to Supplementary Figure 7a-c and Source Data Supplementary Data 1).

File Name: Supplementary Data 2
Description: **Drug susceptibility profiles of AcrB substitution variants. a,** ansamycins, **b,** macrolides, **c,** TMD-BP pathway. Data are mean s.e.m. of N = ≥3-4 biologically independent cells. * represents p value = 0.005-0.05 (Students t-test, two-sided); ** represents p value < 0.005 (Students t-test, two-sided). Growth of the cells: red, ≤0.6 (severely affected); light red, 0.6-0.8 (affected if statistically significant, marked with * or **); lightest red, 0.8-0.9 (not affected even though the growth is lesser than AcrB wildtype); blue, >1.3; light blue, 1.2-1.3; lightest blue, 1.1-1.2 (refer to Supplementary Figure 7d-f and Source Data Supplementary Data 2).

File Name: Supplementary Data 3
Description: **Data collection and refinement statistics**
