# Supplementary material for: Allosteric drug transport mechanism of multidrug transporter AcrB
Source: Nat Commun. 2021 Jun 29;12:3889. doi: 10.1038/s41467-021-24151-3 (PMC8242077; doi:10.1038/s41467-021-24151-3)
Supplement: Supplementary file 7 — Reporting Summary [file 41467_2021_24151_MOESM7_ESM.pdf]

nature research

Corresponding author(s): Klaas M. Pos, Heng-Keat Tam

Last updated by author(s): May 14, 2021

# Reporting Summary

Nature Research wishes to improve the reproducibility of the work that we publish. This form provides structure for consistency and transparency in reporting. For further information on Nature Research policies, see our Editorial Policies and the Editorial Policy Checklist.

## Statistics

For all statistical analyses, confirm that the following items are present in the figure legend, table legend, main text, or Methods section.

| n/a | Confirmed | |
|---|---|---|
| ☐ | ☒ | The exact sample size (*n*) for each experimental group/condition, given as a discrete number and unit of measurement |
| ☐ | ☒ | A statement on whether measurements were taken from distinct samples or whether the same sample was measured repeatedly |
| ☐ | ☒ | The statistical test(s) used AND whether they are one- or two-sided<br>*Only common tests should be described solely by name; describe more complex techniques in the Methods section.* |
| ☒ | ☐ | A description of all covariates tested |
| ☐ | ☒ | A description of any assumptions or corrections, such as tests of normality and adjustment for multiple comparisons |
| ☐ | ☒ | A full description of the statistical parameters including central tendency (e.g. means) or other basic estimates (e.g. regression coefficient) AND variation (e.g. standard deviation) or associated estimates of uncertainty (e.g. confidence intervals) |
| ☐ | ☒ | For null hypothesis testing, the test statistic (e.g. *F*, *t*, *r*) with confidence intervals, effect sizes, degrees of freedom and *P* value noted<br>*Give P values as exact values whenever suitable.* |
| ☒ | ☐ | For Bayesian analysis, information on the choice of priors and Markov chain Monte Carlo settings |
| ☒ | ☐ | For hierarchical and complex designs, identification of the appropriate level for tests and full reporting of outcomes |
| ☒ | ☐ | Estimates of effect sizes (e.g. Cohen's *d*, Pearson's *r*), indicating how they were calculated |

*Our web collection on statistics for biologists contains articles on many of the points above.*

## Software and code

Policy information about availability of computer code

| | |
|---|---|
| Data collection | ImageQuant TL 8.1 for the images on drug susceptibility and crosslinking experiments. |
| Data analysis | XDS (version November 3, 2014, version BUILT=20161205, version BUILT=20161101, version March 1, 2015; version BUILT=20180808; version BUILT=20160617), Coot 0.89, Pymol 2.4.0a0, CCP4i version 7, Phenix 1.17.1, Molprobity, BUSTER 2.10.3 (Global Phasing Ltd), STARANISO (Global Phasing Ltd), ImageJ 1.52o, Prism 7.0, phenix.polder within Phenix package 1.17.1, Refmac5 within CCP4i package version 7. |

For manuscripts utilizing custom algorithms or software that are central to the research but not yet described in published literature, software must be made available to editors and reviewers. We strongly encourage code deposition in a community repository (e.g. GitHub). See the Nature Research guidelines for submitting code & software for further information.

## Data

Policy information about availability of data

All manuscripts must include a data availability statement. This statement should provide the following information, where applicable:

- Accession codes, unique identifiers, or web links for publicly available datasets
- A list of figures that have associated raw data
- A description of any restrictions on data availability

Atomic coordinates and structure factors reported in this paper have been deposited in the Protein Data Bank under accession numbers 6ZO5 (AcrB-Gly619Pro_Gly621Pro/DARPin in the presence of fusidic acid), 6ZO6 (AcrB-Gly619Pro/DARPin in the presence of minocycline), 6ZO7 (AcrB-Gly619Pro/DARPin in the presence of 3-formylrifamycin SV), 6ZO8 (AcrB-Gly621Pro/DARPin in the presence of minocycline), 6ZO9 (AcrB-Gly621Pro/DARPin in the presence of rifabutin), 6ZOA (AcrB/DARPin L2-T-O conformation with DDM bound to TM8/PC2 tunnel), 6ZOB (AcrB/DARPin in the presence of 3-formylrifamycin SV), 6ZOC (AcrB-Gly616Pro/DARPin in the presence of erythromycin and 3-formylrifamycin SV), 6ZOD (AcrB/DARPin in the presence of fusidic acid, fully induced T state), 6ZOE (AcrB-Phe563Ala/DARPin), 6ZOF (AcrB-Phe380Ala/DARPin in the presence of fusidic acid), 6ZOG (AcrB-Ile38Phe_Ile671Thr/DARPin in the presence of minocycline) and

6ZOH (AcrB-Gly619Pro_Gly621Pro/DARPin in the presence of 3-formylrifamycin SV). Atomic coordinates that support the findings of this study are available in the Protein Data Bank under accession numbers 2J8S, 3AOC, 3W9I, 4DX5, 4ZJL, 5JMN, 6ZO5, 6ZO6, 6ZO7, 6ZO8, 6ZO9, 6ZOA, 6ZOB, 6ZOC, 6ZOD, 6ZOE, 6ZOF, 6ZOG, and 6ZOH. Source Data for Fig. 6c, Supplementary Fig 1b, 4e-f, and Supplementary Supplementary Data 1-2 are provided with this paper.

# Field-specific reporting

Please select the one below that is the best fit for your research. If you are not sure, read the appropriate sections before making your selection.

☒ Life sciences ☐ Behavioural & social sciences ☐ Ecological, evolutionary & environmental sciences

For a reference copy of the document with all sections, see nature.com/documents/nr-reporting-summary-flat.pdf

# Life sciences study design

All studies must disclose on these points even when the disclosure is negative.

| | |
|---|---|
| Sample size | In drug agar plate assay, sample size of at least N = 3-4 correspond to independent setup of biological cultures, which were defined by a setup of freshly prepared transformants, freshly picked cultures, and freshly prepared agar plate in the presence of freshly prepared antibiotics. No technical replicates from each independent setup were included in data analysis. All the data processing and data analysis were by means of independent biological setup.<br>All biochemical assays (substrate protection crosslinking experiments) were performed independently with the sample size of N = 3-4 unless noted. |
| Data exclusions | No data were excluded. |
| Replication | All the drug agar plate assay were repeated at least 3-4 times independently over a few weeks for each variants. The experiments were repeated with independent cells and freshly prepared antibiotics. No technical replicates from each cells were included for data analysis. Therefore, attempts of data replication were successful.<br>All the substrate protection crosslinking experiments were repeated at least 3-4 times independently over a few weeks with freshly purified proteins, freshly prepared antibiotics, freshly prepared SDS gels, and fresh aliquots of crosslinker, MTS-rhodamine. Therefore, attempts of data replication were successful. |
| Randomization | Randomization is not applicable.<br>In drug agar plate assay, each of the cells from independent AcrB variants are clones or transformants. In principle, colonies were picked randomly from the agar plates after transformation of plasmid into E. coli cells.<br>Substrate protection crosslinking assay is a biochemical assays of substrate binding, which requires a rationale data collection and analysis. |
| Blinding | Not applicable to all the experiments due to the need for rationale design. Negative and positive controls were included in each of the experiments. |

# Reporting for specific materials, systems and methods

We require information from authors about some types of materials, experimental systems and methods used in many studies. Here, indicate whether each material, system or method listed is relevant to your study. If you are not sure if a list item applies to your research, read the appropriate section before selecting a response.

## Materials & experimental systems

| n/a | Involved in the study | |
|---|---|---|
| ☐ | ☒ | Antibodies |
| ☒ | ☐ | Eukaryotic cell lines |
| ☒ | ☐ | Palaeontology and archaeology |
| ☒ | ☐ | Animals and other organisms |
| ☒ | ☐ | Human research participants |
| ☒ | ☐ | Clinical data |
| ☒ | ☐ | Dual use research of concern |

## Methods

| n/a | Involved in the study | |
|---|---|---|
| ☒ | ☐ | ChIP-seq |
| ☒ | ☐ | Flow cytometry |
| ☒ | ☐ | MRI-based neuroimaging |

## Antibodies

| | |
|---|---|
| Antibodies used | anti-AcrB antibody (Custom made antibody by Neosystems, France), anti-rabbit IgG alkaline phosphatase antibody (Product # A3687, Sigma-Aldrich, USA) |
| Validation | All the commercial antibodies were verified by the manufacturers according to immunoblots and/or image on their websites (Sigma-Aldrich refers for Western Blot analysis validation to Cibelli et al., 2001 (PMID: 11298794 DOI: 10.1046/j.0953-816x.2001.01510.x). Anti-AcrB has been validated in our lab with purified AcrB as sample (e.g. Seeger MA, et al. (2008) Engineered disulfide bonds support the functional rotation mechanism of multidrug efflux pump AcrB. Nat Struct Mol Biol 15:199–205). |
