# Supplementary material for: Allosteric drug transport mechanism of multidrug transporter AcrB
Source: Nat Commun. 2021 Jun 29;12:3889. doi: 10.1038/s41467-021-24151-3 (PMC8242077; doi:10.1038/s41467-021-24151-3)
Supplement: Supplementary file 8 — Source Data [file 41467_2021_24151_MOESM8_ESM.zip › Source_Data_Supplementary_Data-1_2c.zip!/Source_Data_Supplementary_Data-1_Data-2c_Chloramphenicol.pdf]

Chloramphenicol (0.75–1.0 μg/ml)

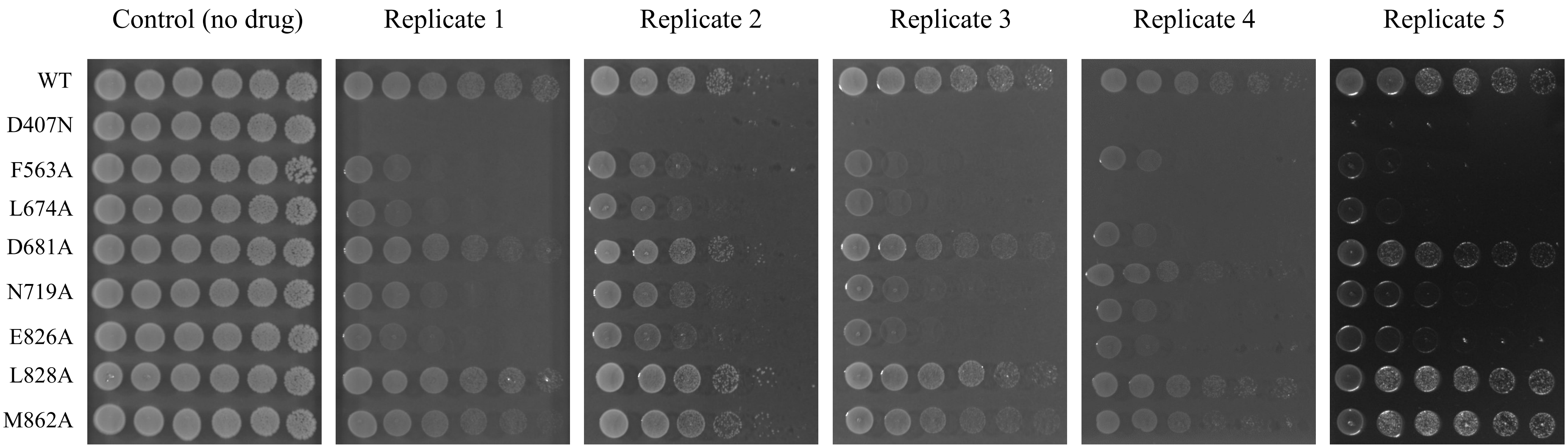


Control (no drug) – representative

Cells with $OD_{600}$ of $10^{-1}$–$10^{-6}$ were pipetted from left to right.

Chloramphenicol (1.0 µg/ml)

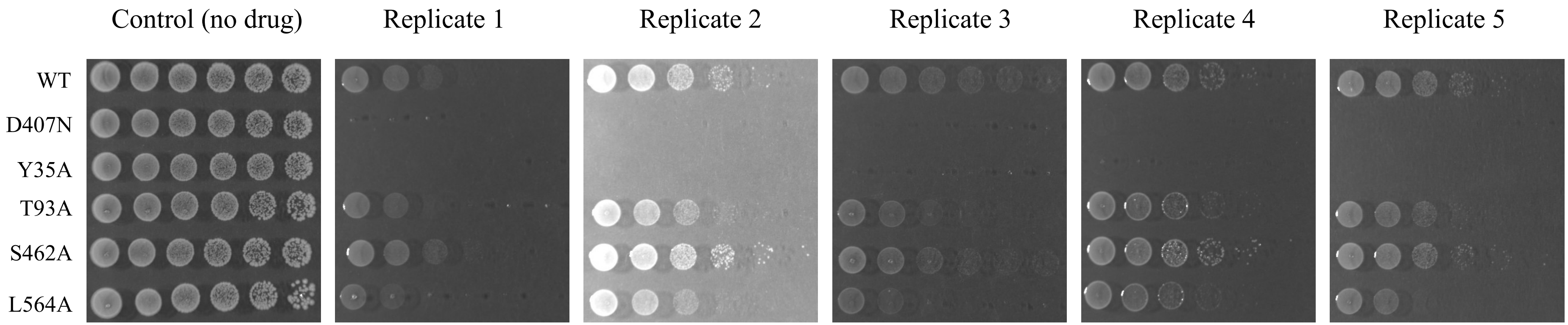


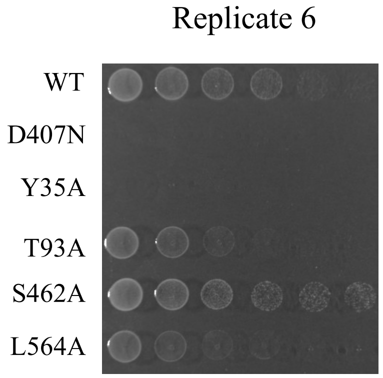


Control (no drug) – representative

Cells with $OD_{600}$ of $10^{-1}$–$10^{-6}$ were pipetted from left to right.

Chloramphenicol (0.75–1.0 μg/ml)

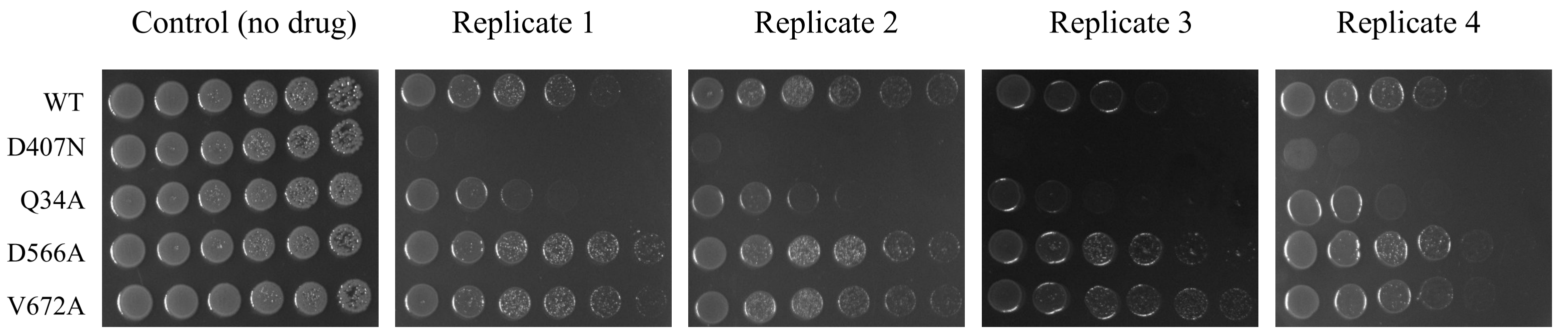


Control (no drug) – representative

Cells with $OD_{600}$ of $10^{-1}$–$10^{-6}$ were pipetted from left to right.

Chloramphenicol (1.0 µg/ml)

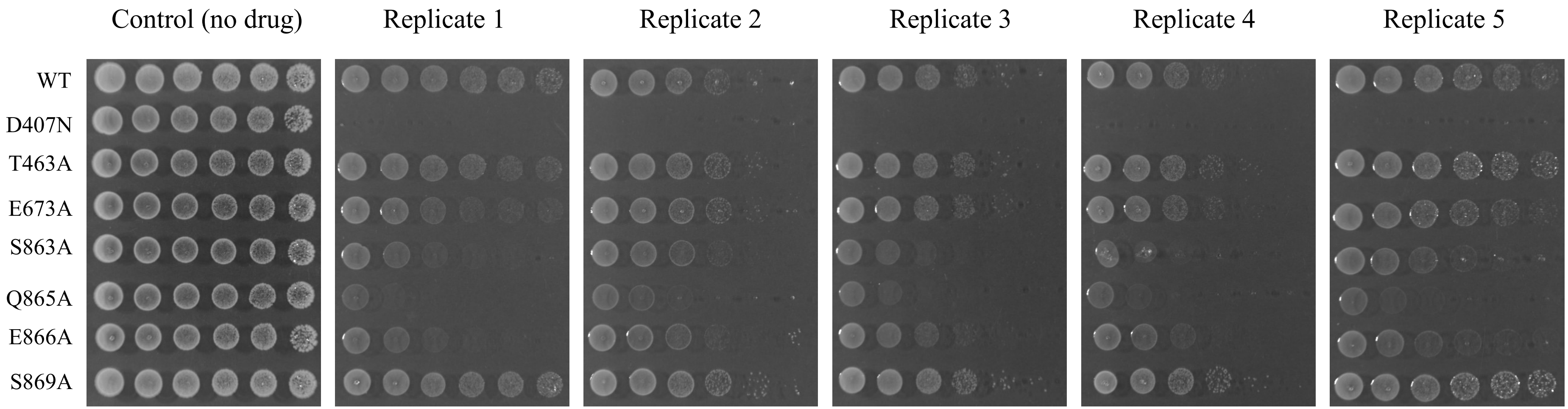


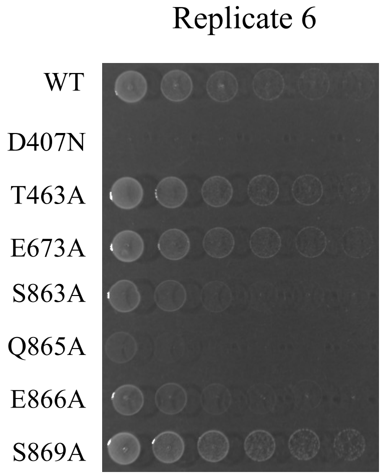


Control (no drug) – representative

Cells with $OD_{600}$ of $10^{-1}$–$10^{-6}$ were pipetted from left to right.

Chloramphenicol (1.0 μg/ml)

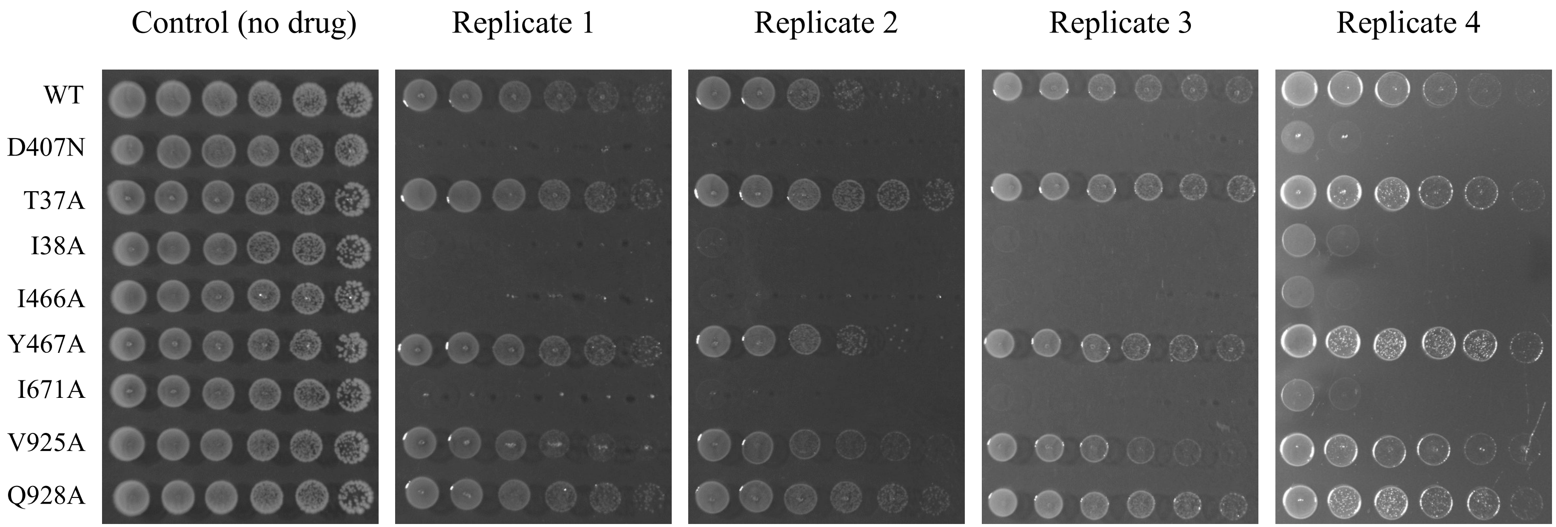


Control (no drug) – representative

Cells with $OD_{600}$ of $10^{-1}$–$10^{-6}$ were pipetted from left to right.

Chloramphenicol (1.0 μg/ml)

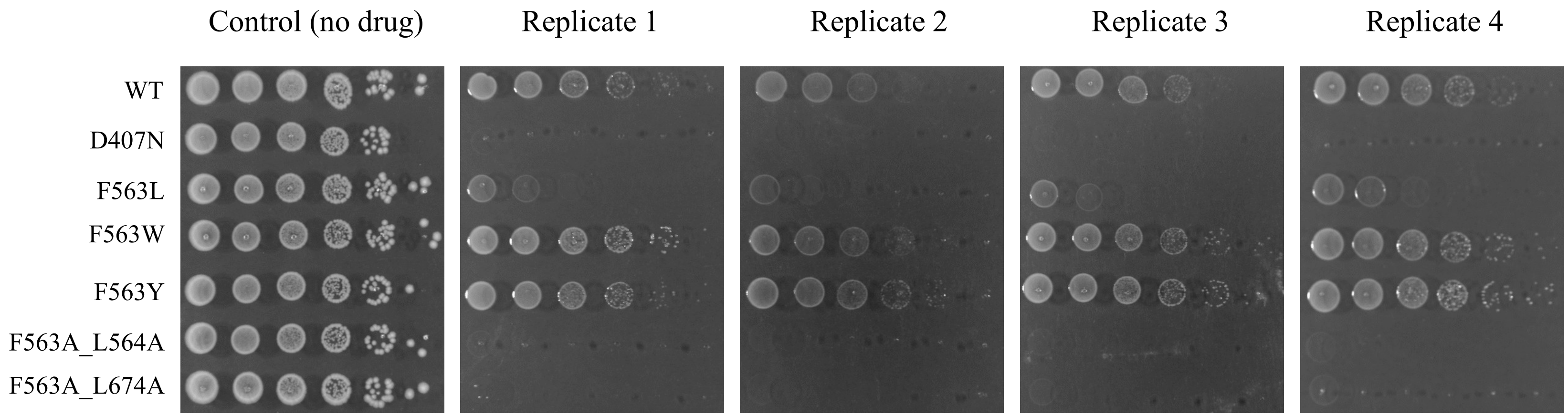


Control (no drug) – representative

Cells with $OD_{600}$ of $10^{-1}$–$10^{-6}$ were pipetted from left to right.

Chloramphenicol (0.75–1.0 μg/ml)

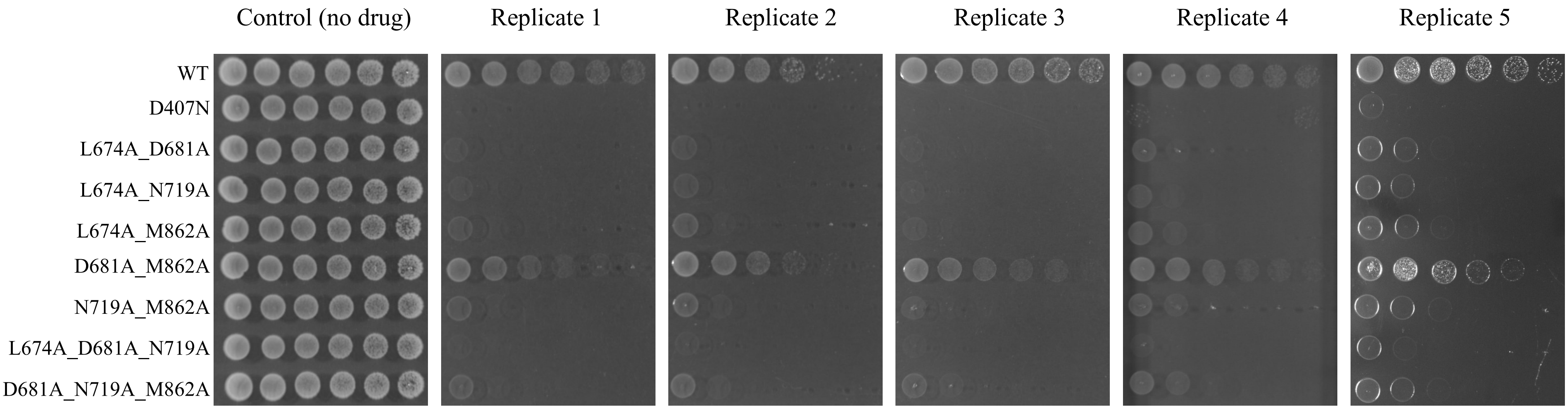


Control (no drug) – representative

Cells with $OD_{600}$ of $10^{-1}$–$10^{-6}$ were pipetted from left to right.

Chloramphenicol (0.75–1.0 μg/ml)

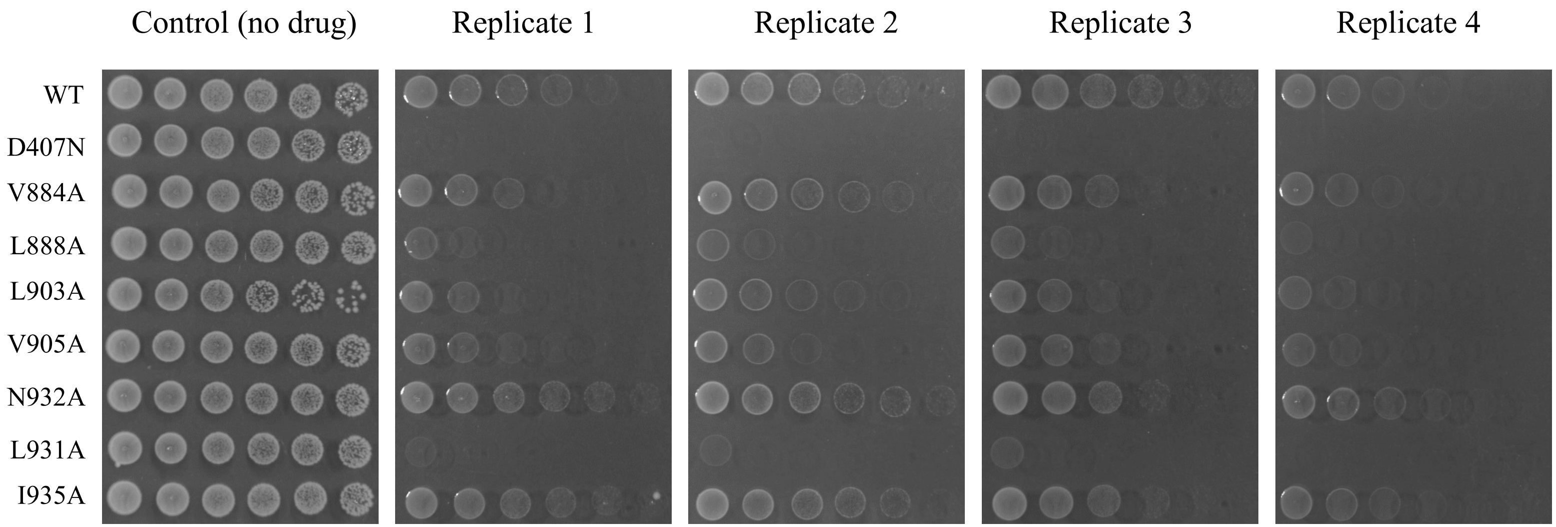


Control (no drug) – representative

Cells with $OD_{600}$ of $10^{-1}$–$10^{-6}$ were pipetted from left to right.

Chloramphenicol (1.0 μg/ml)

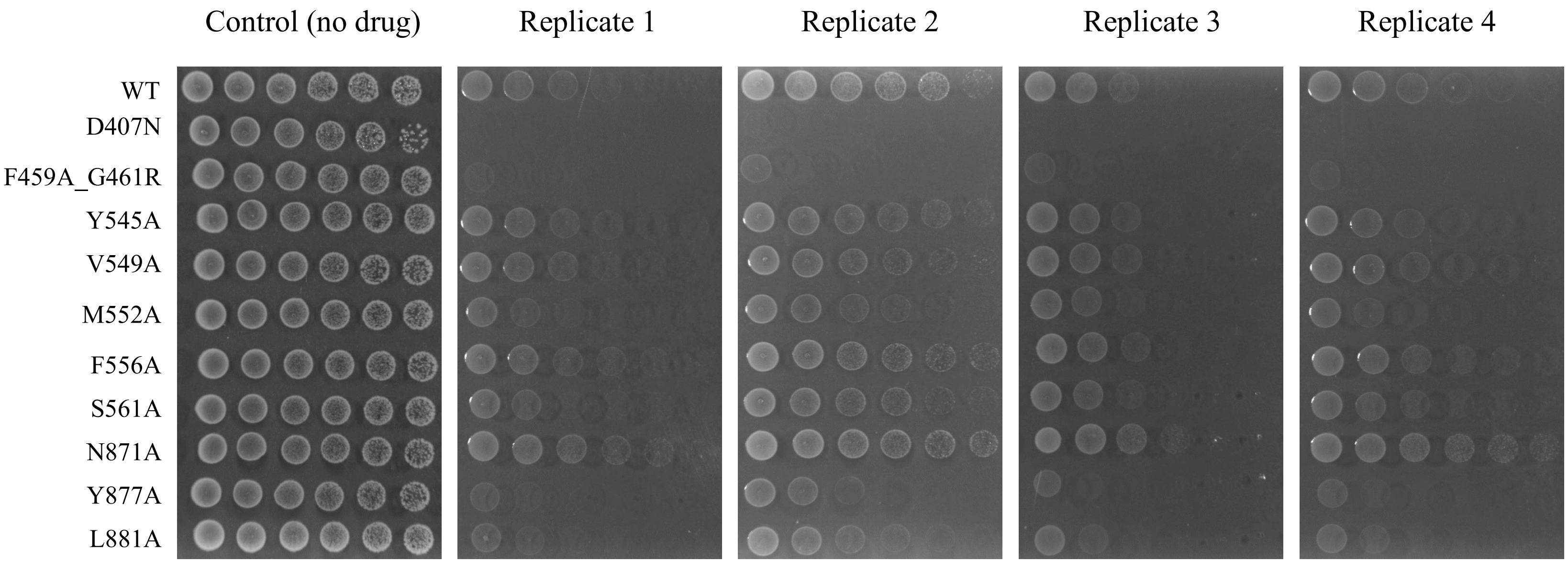


Control (no drug) – representative

Cells with $OD_{600}$ of $10^{-1}$–$10^{-6}$ were pipetted from left to right.

Chloramphenicol (1.0 μg/ml)

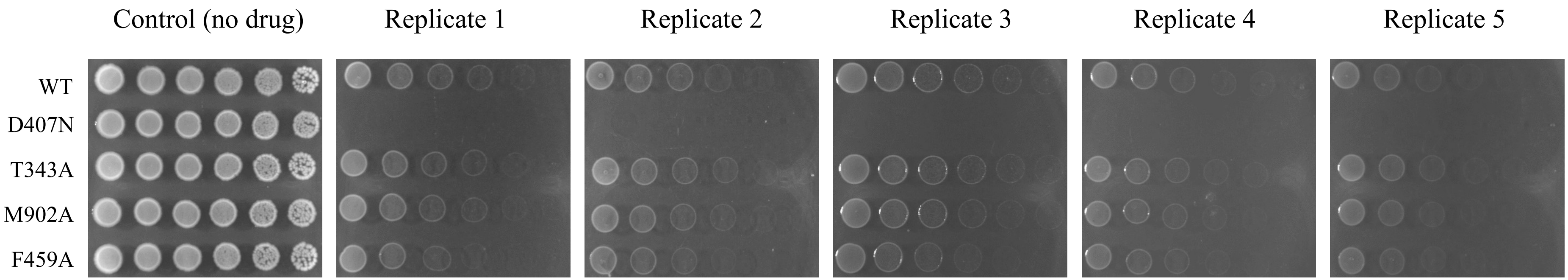


Control (no drug) – representative

Cells with $OD_{600}$ of $10^{-1}$–$10^{-6}$ were pipetted from left to right.

Chloramphenicol (1.0 μg/ml)

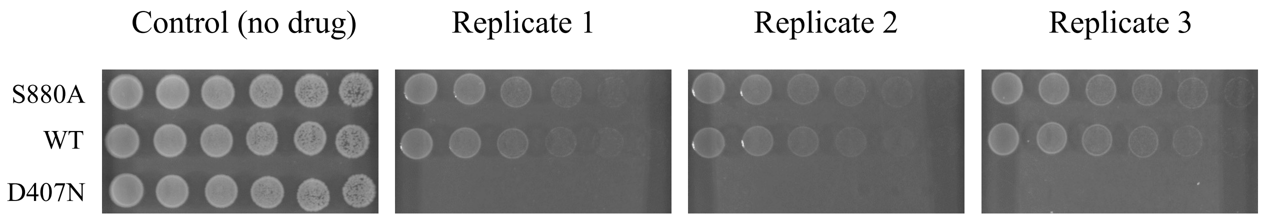


Control (no drug) – representative

Cells with $OD_{600}$ of $10^{-1}$–$10^{-6}$ were pipetted from left to right.

Chloramphenicol (0.75–1.0 µg/ml)

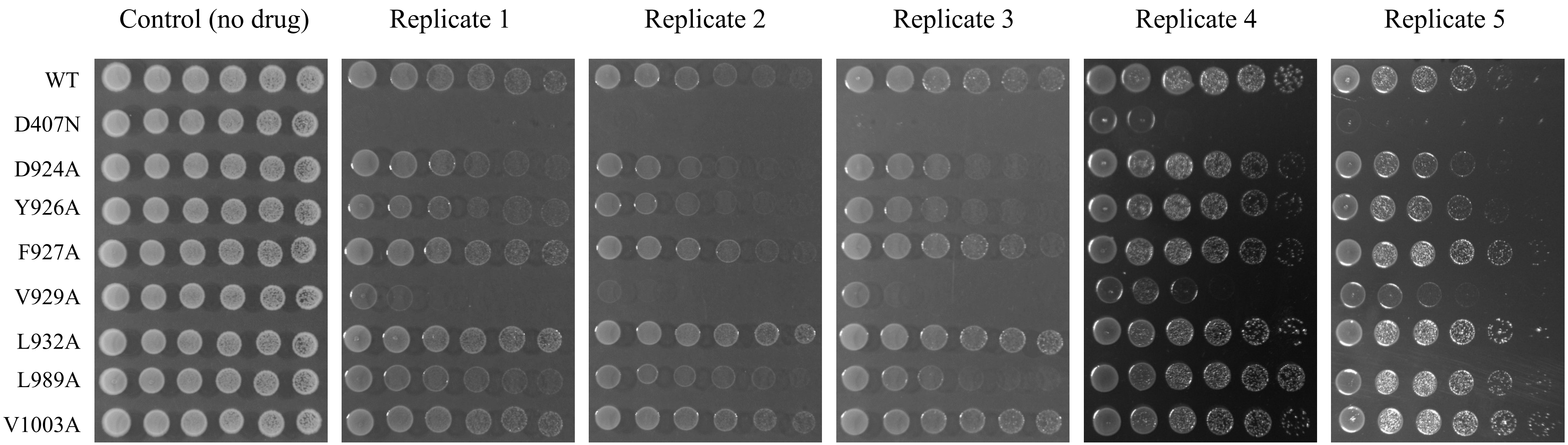


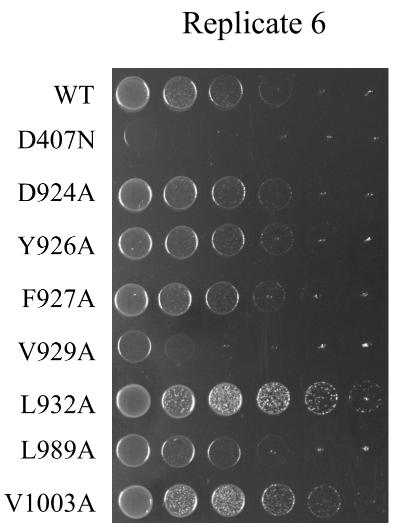


Control (no drug) – representative

Cells with $OD_{600}$ of $10^{-1}$–$10^{-6}$ were pipetted from left to right.

Chloramphenicol (0.75–1.0 μg/ml)

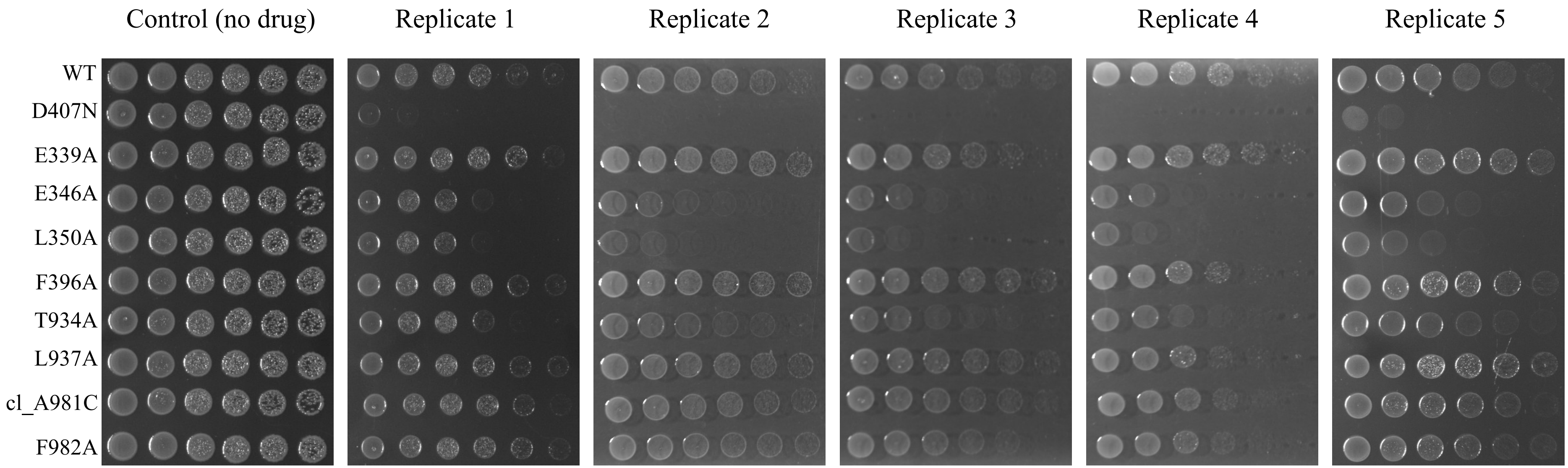


Control (no drug) – representative

Cells with $OD_{600}$ of $10^{-1}$–$10^{-6}$ were pipetted from left to right.

Chloramphenicol (1.0 µg/ml)

Control (no drug) | Replicate 2 | Replicate 3 | Replicate 4 | Replicate 5

WT
D407N
F453A
F470A
I474A
M478A
E567A
L921A

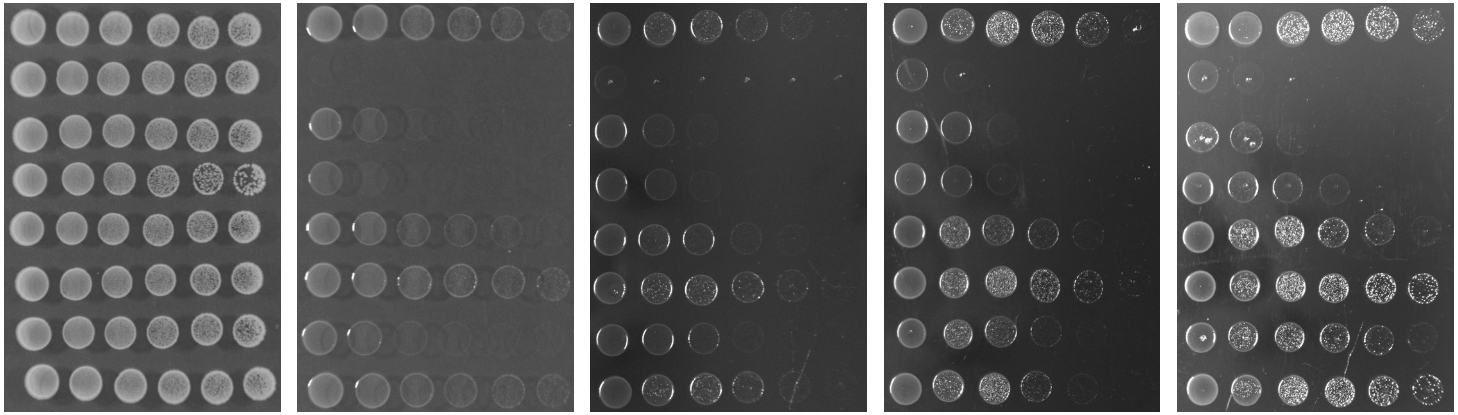

Replicate 1

WT
F453A
D407N
F470A
I474A
M478A
E567A
L921A

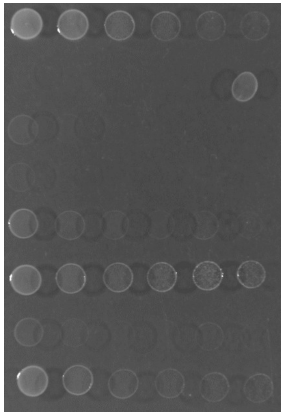

Replicate 1: only F453A was pipetted from $OD_{600}$ of $10^{-1}$ to $10^{-6}$ from right to left.

Control (no drug) – representative

Cells with $OD_{600}$ of $10^{-1}$–$10^{-6}$ were pipetted from left to right.

Chloramphenicol (1.0 μg/ml)

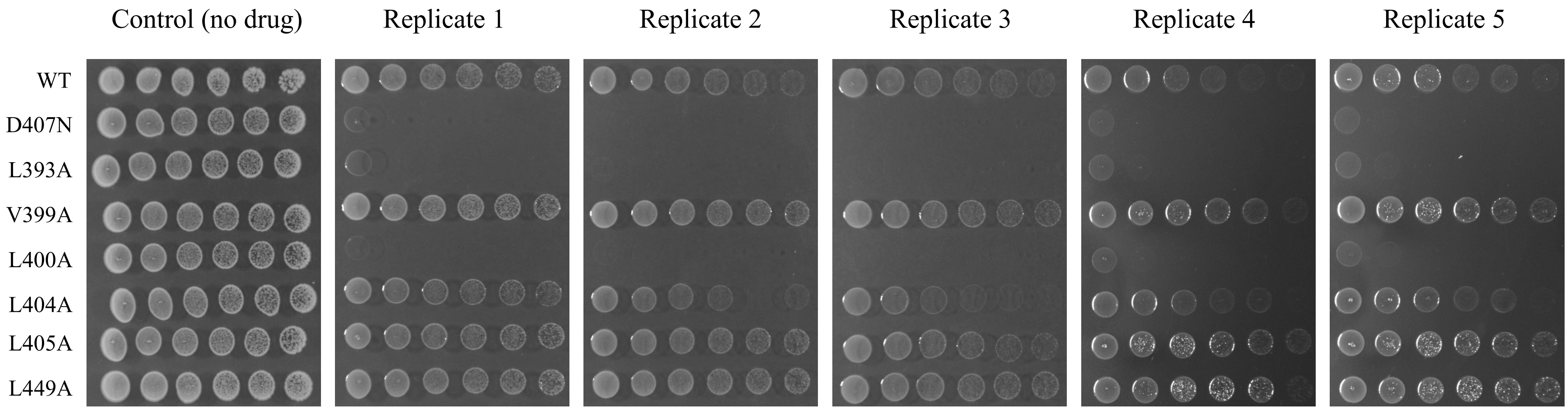


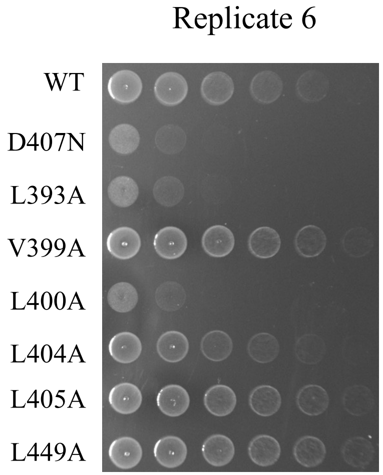


Control (no drug) – representative

Cells with $OD_{600}$ of $10^{-1}$–$10^{-6}$ were pipetted from left to right.
