# Supplementary material for: Allosteric drug transport mechanism of multidrug transporter AcrB
Source: Nat Commun. 2021 Jun 29;12:3889. doi: 10.1038/s41467-021-24151-3 (PMC8242077; doi:10.1038/s41467-021-24151-3)
Supplement: Supplementary file 8 — Source Data [file 41467_2021_24151_MOESM8_ESM.zip › Source_Data_Supplementary_Data-1_2c.zip!/Source_Data_Supplementary_Data-1_Data-2c_Cloxacillin.pdf]

Cloxacillin (40 µg/ml)

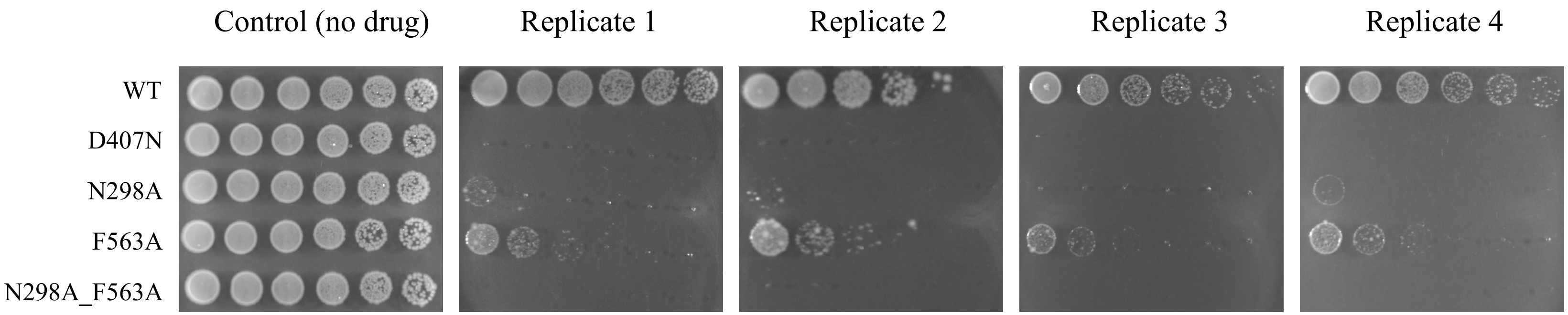

Control (no drug) – representative

Cells with OD_{600} of 10^{-1}–10^{-6} were pipetted from left to right.

Cloxacillin (40–45 μg/ml)

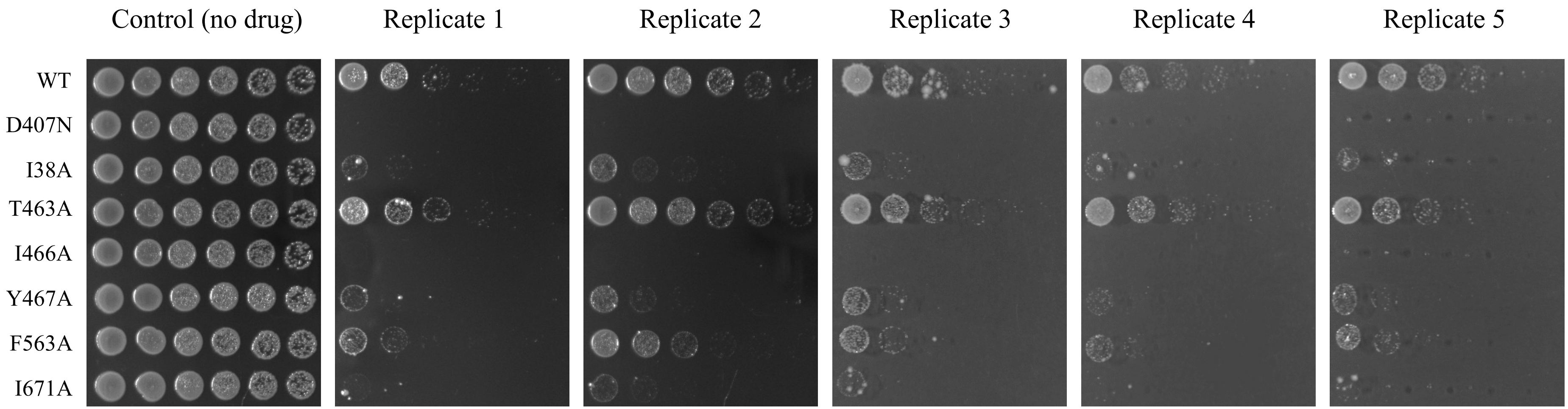

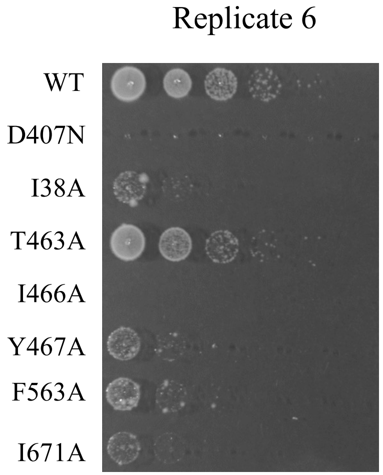

Control (no drug) – representative

Cells with OD_{600} of 10^{-1}–10^{-6} were pipetted from left to right.

Cloxacillin (45–50 µg/ml)

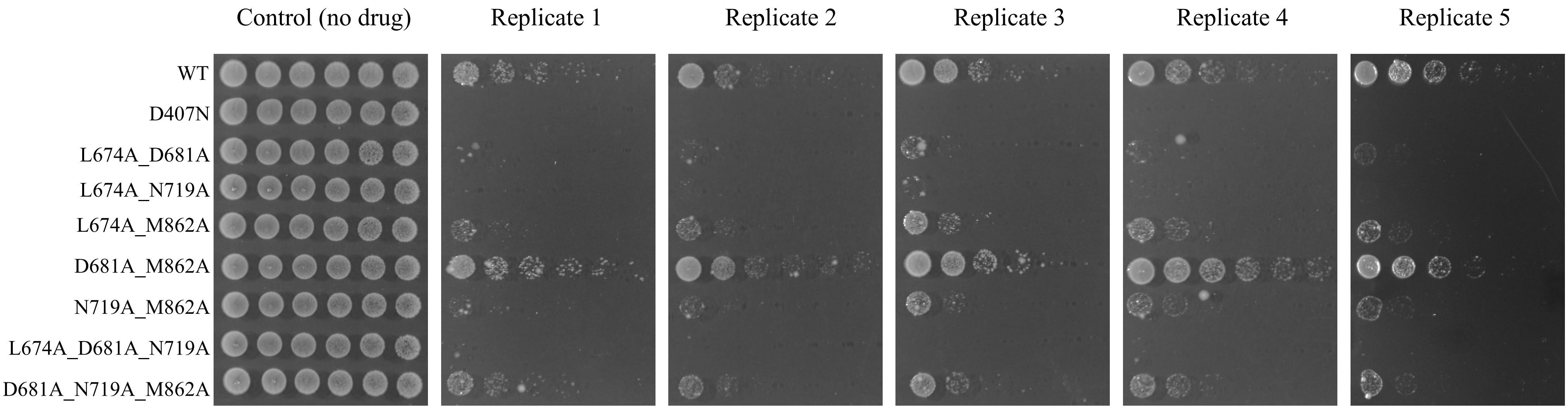

Control (no drug) – representative

Cells with OD_{600} of 10^{-1}–10^{-6} were pipetted from left to right.

Cloxacillin (35 μg/ml)

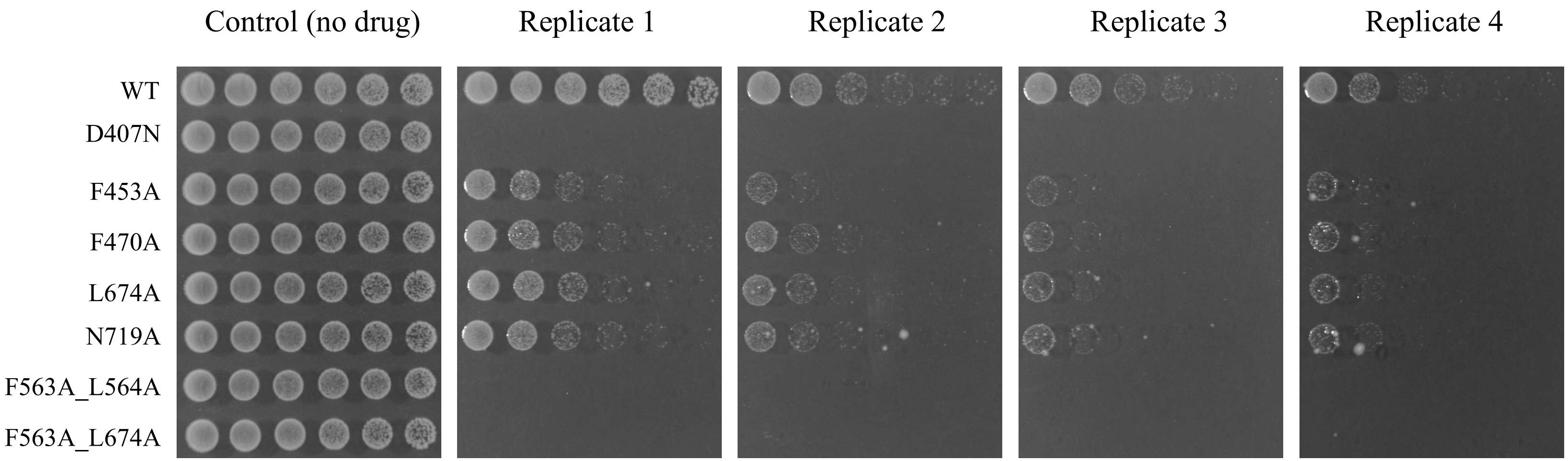

Control (no drug) – representative

Cells with OD_{600} of 10^{-1}–10^{-6} were pipetted from left to right.

Cloxacillin (35–38 μg/ml)

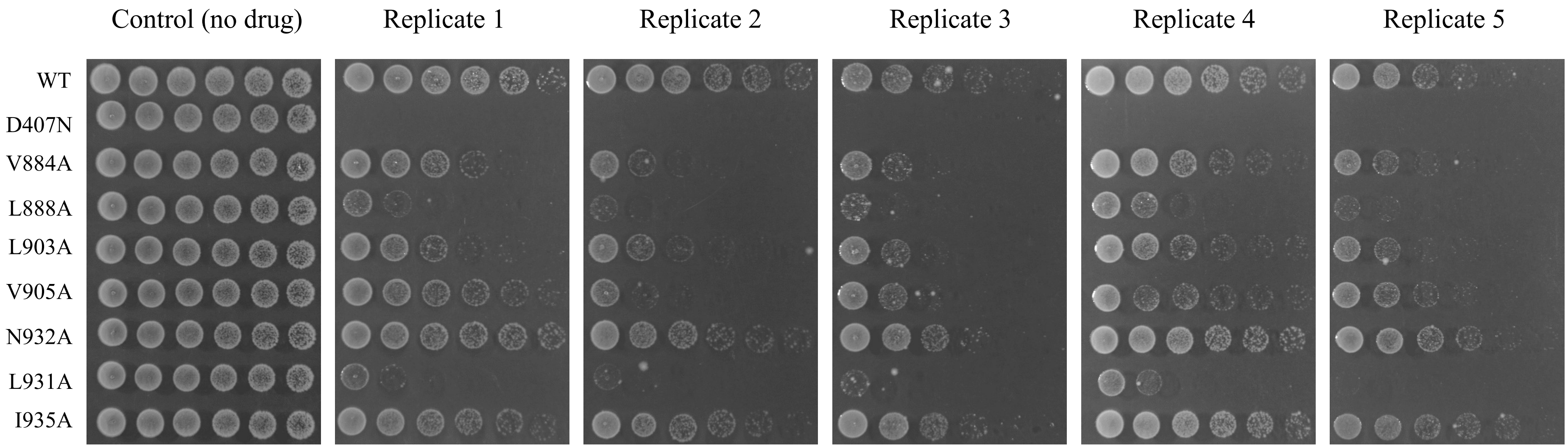

Control (no drug) – representative

Cells with OD_{600} of 10^{-1}–10^{-6} were pipetted from left to right.

Cloxacillin (35–38 μg/ml)

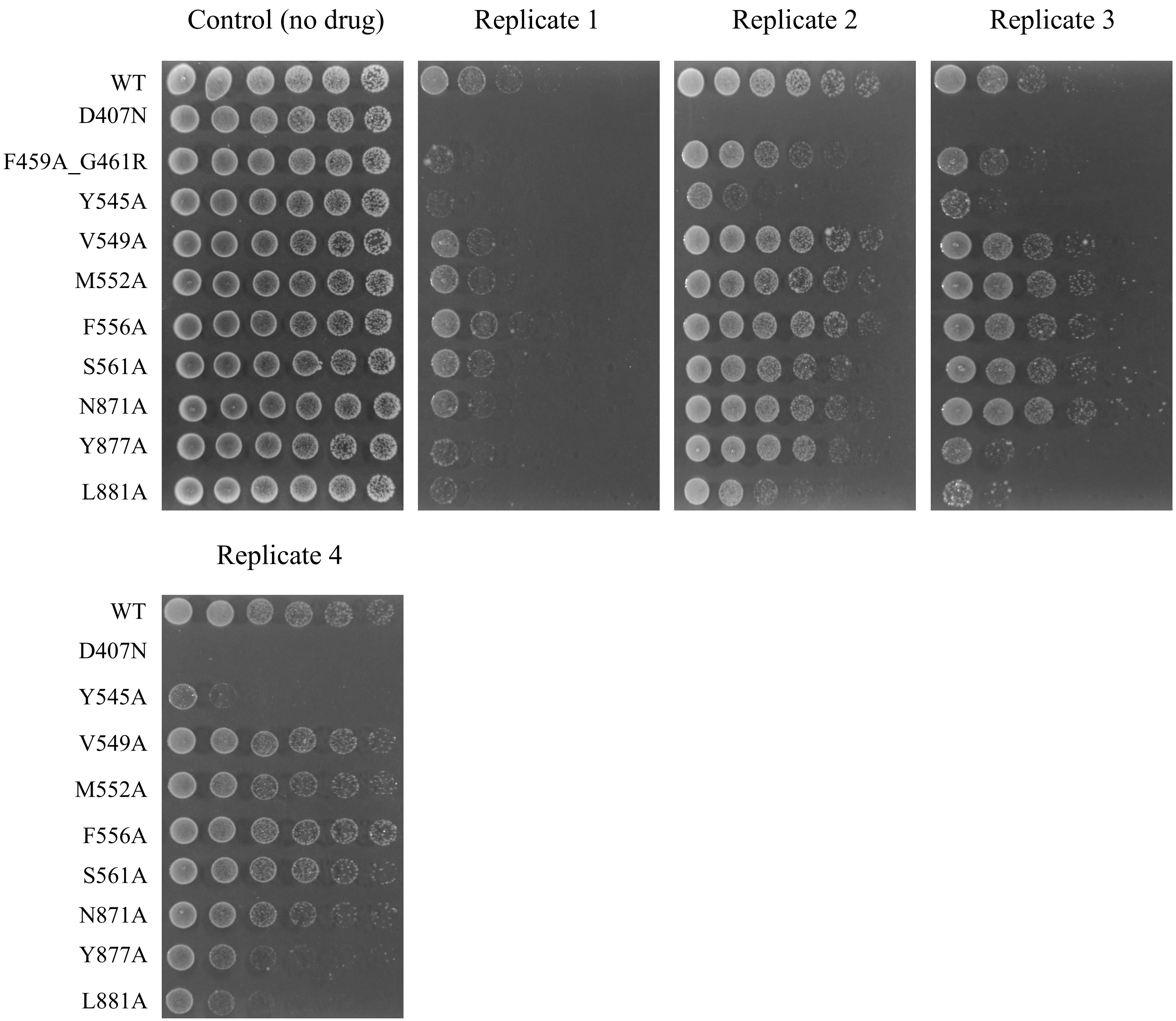

Control (no drug) – representative

Cells with OD_{600} of 10^{-1}–10^{-6} were pipetted from left to right.

Cloxacillin (35–38 μg/ml)

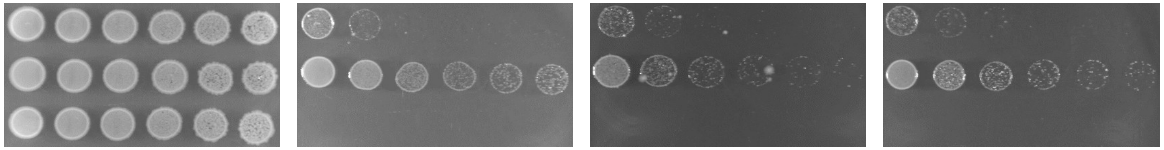

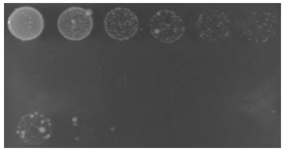

Control (no drug) – representative

Cells with OD_{600} of 10^{-1}–10^{-6} were pipetted from left to right.

Cloxacillin (28–38 μg/ml)

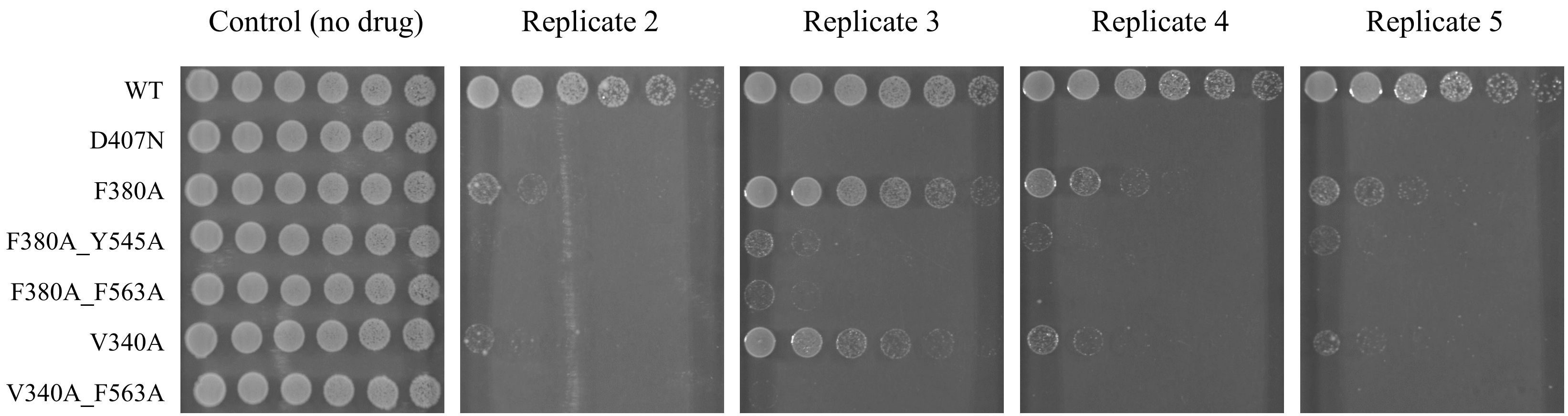

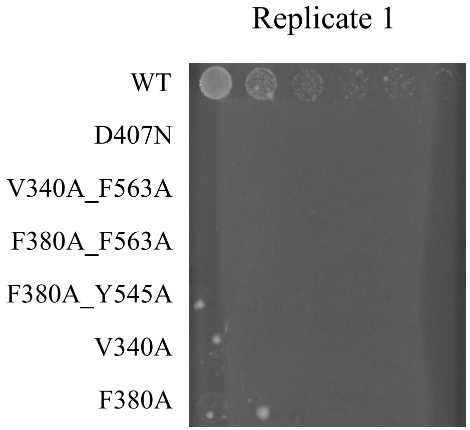

Control (no drug) – representative

Cells with OD_{600} of 10^{-1}–10^{-6} were pipetted from left to right.

Cloxacillin (35–40 μg/ml)

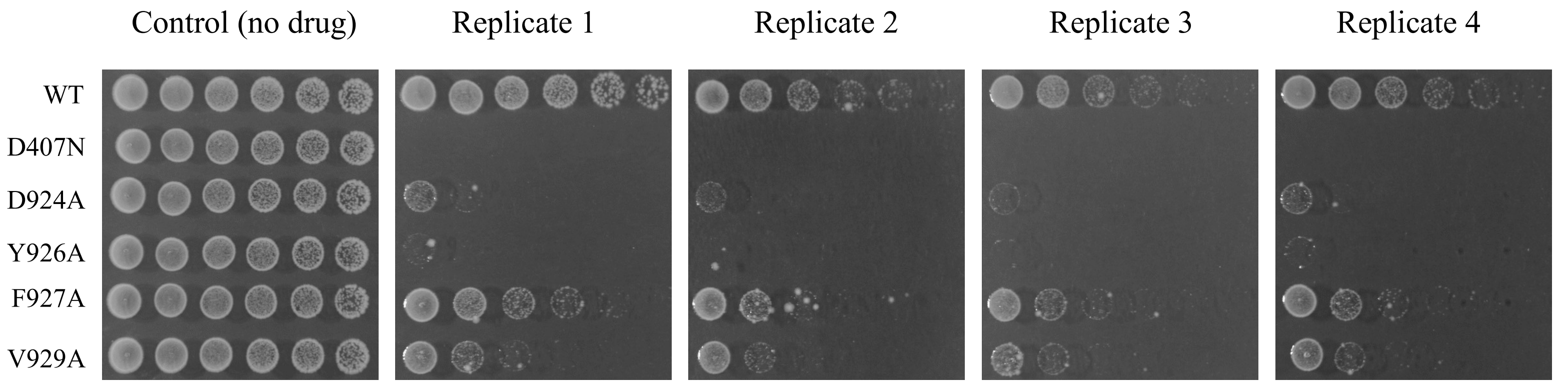

Control (no drug) – representative

Cells with OD_{600} of 10^{-1}–10^{-6} were pipetted from left to right.

Cloxacillin (30–35 μg/ml)

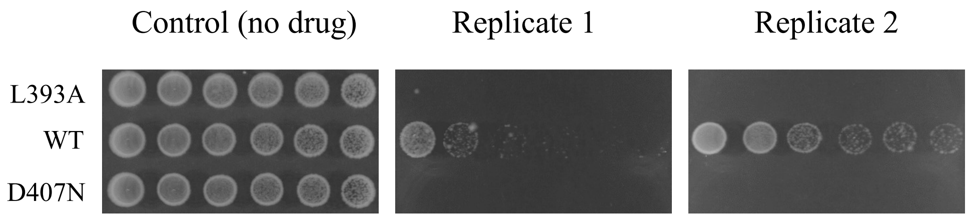

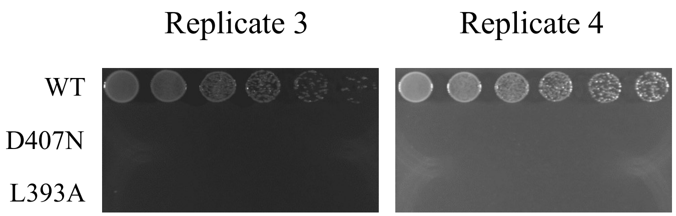

Control (no drug) – representative

Cells with OD_{600} of 10^{-1}–10^{-6} were pipetted from left to right.
