# Supplementary material for: Allosteric drug transport mechanism of multidrug transporter AcrB
Source: Nat Commun. 2021 Jun 29;12:3889. doi: 10.1038/s41467-021-24151-3 (PMC8242077; doi:10.1038/s41467-021-24151-3)
Supplement: Supplementary file 8 — Source Data [file 41467_2021_24151_MOESM8_ESM.zip › Source_Data_Supplementary_Data-1_2c.zip!/Source_Data_Supplementary_Data-1_Data-2c_Fusidicacid.pdf]

Fusidic acid (15 μg/ml)

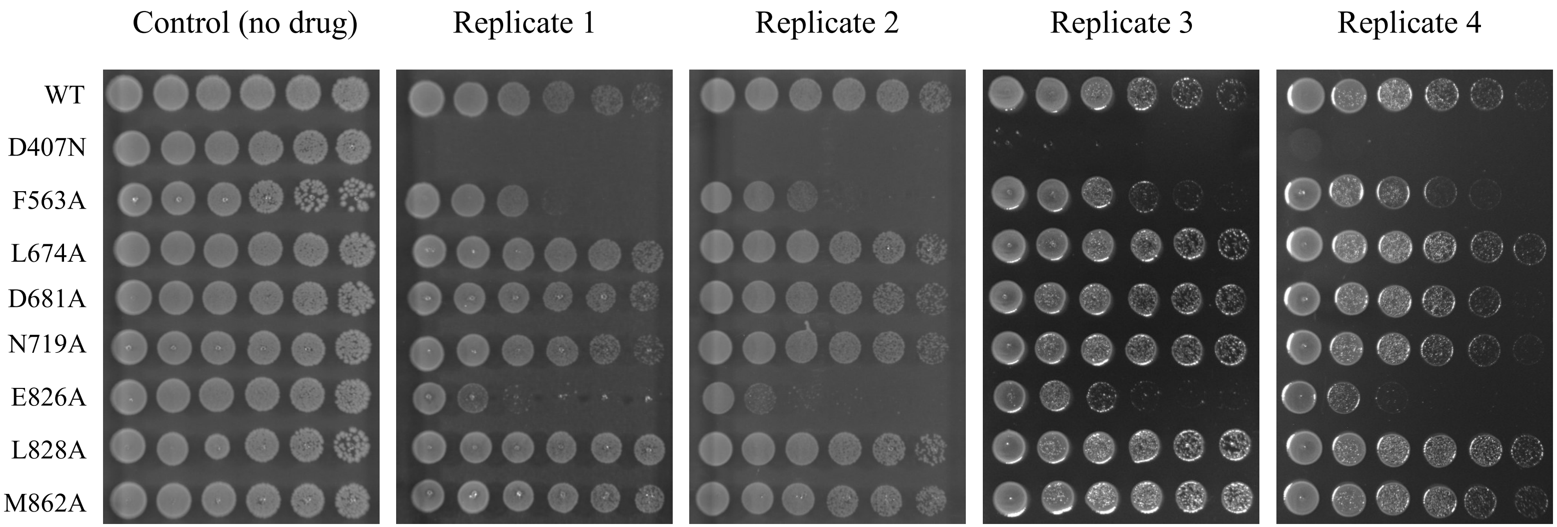


Control (no drug) – representative

Cells with $OD_{600}$ of $10^{-1}$–$10^{-6}$ were pipetted from left to right.

Fusidic acid (15 μg/ml)

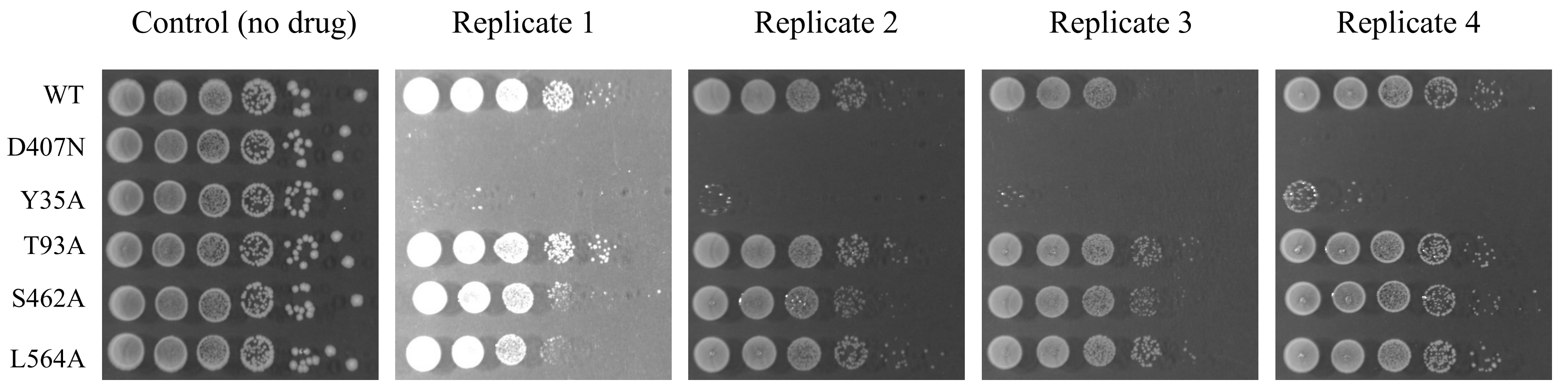


Control (no drug) – representative

Cells with $OD_{600}$ of $10^{-1}$–$10^{-6}$ were pipetted from left to right.

Fusidic acid (15–16 µg/ml)

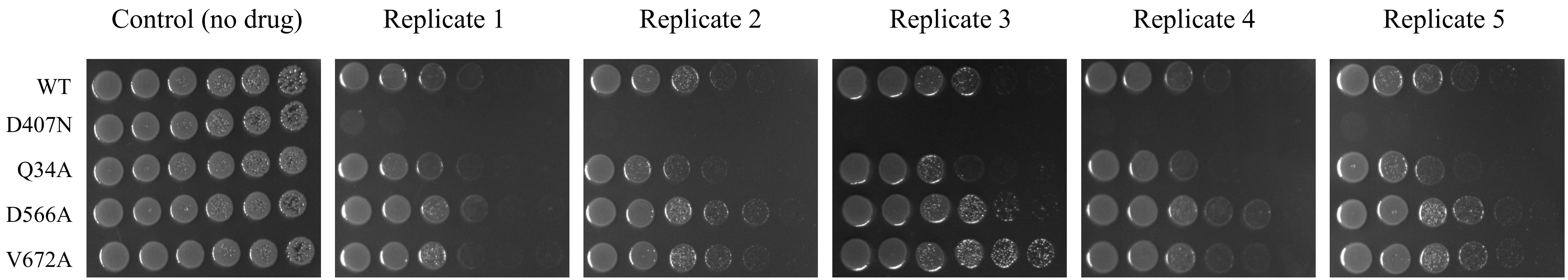


Control (no drug) – representative

Cells with $OD_{600}$ of $10^{-1}$–$10^{-6}$ were pipetted from left to right.

Fusidic acid (15 μg/ml)

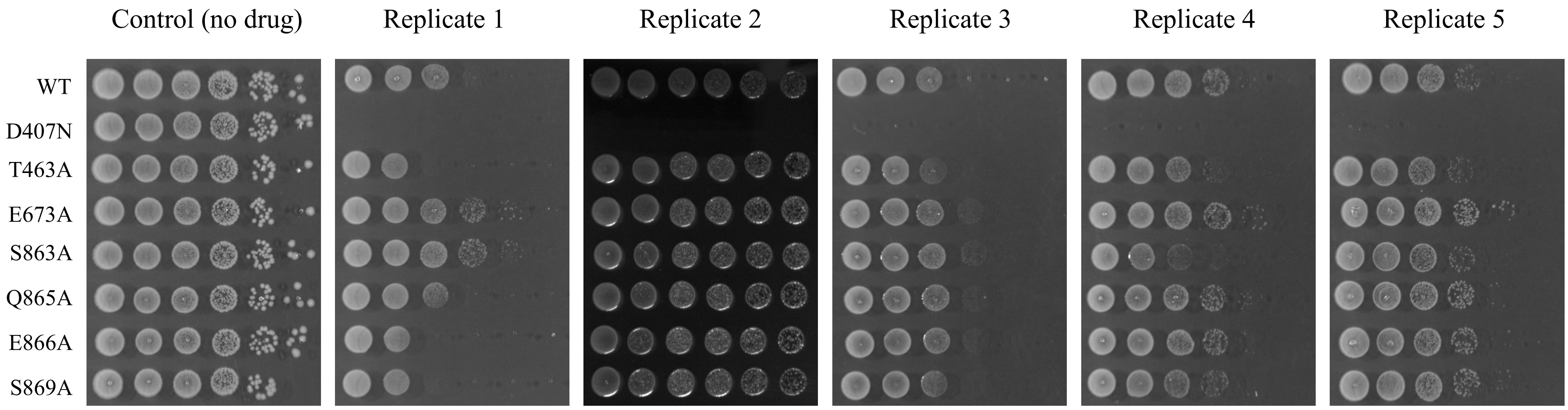


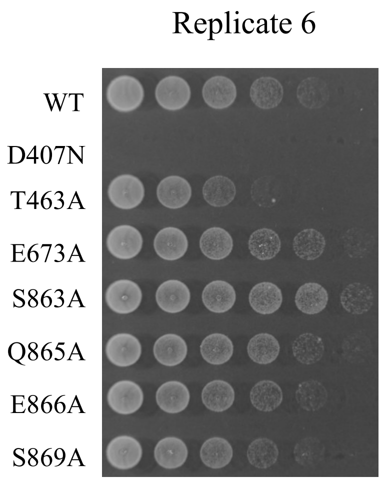


Control (no drug) – representative

Cells with $OD_{600}$ of $10^{-1}$–$10^{-6}$ were pipetted from left to right.

Fusidic acid (15 µg/ml)

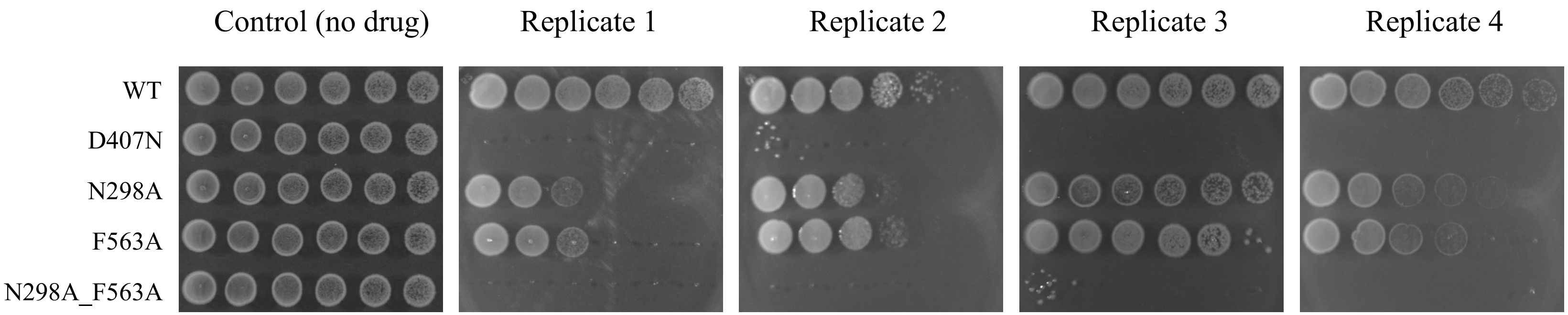


Control (no drug) – representative

Cells with $OD_{600}$ of $10^{-1}$–$10^{-6}$ were pipetted from left to right.

Fusidic acid (15 μg/ml)

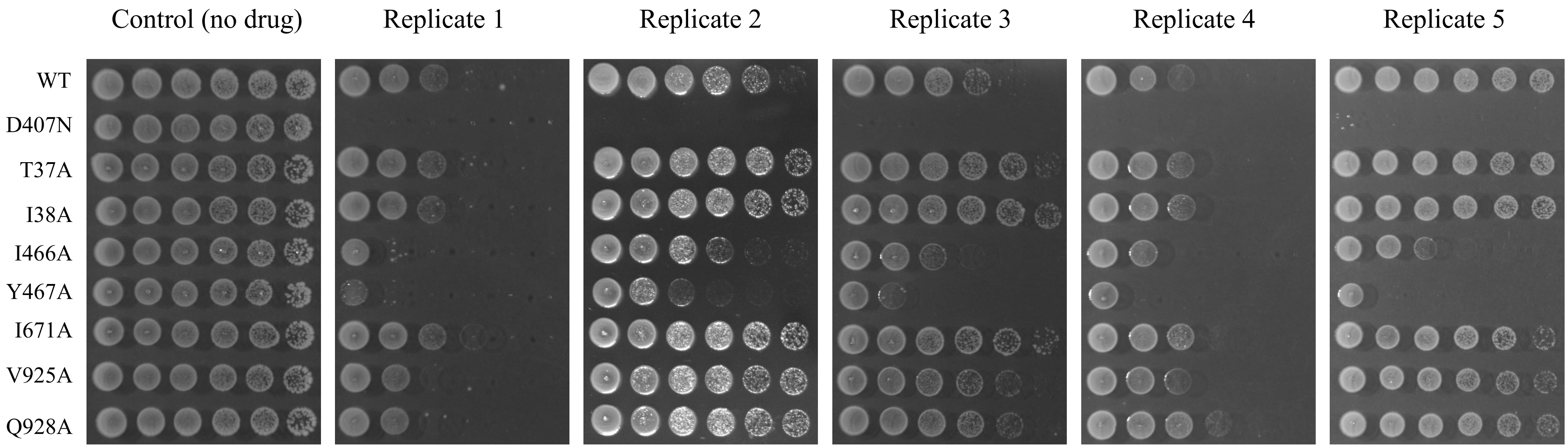


Control (no drug) – representative

Cells with $OD_{600}$ of $10^{-1}$–$10^{-6}$ were pipetted from left to right.

Fusidic acid (15 μg/ml)

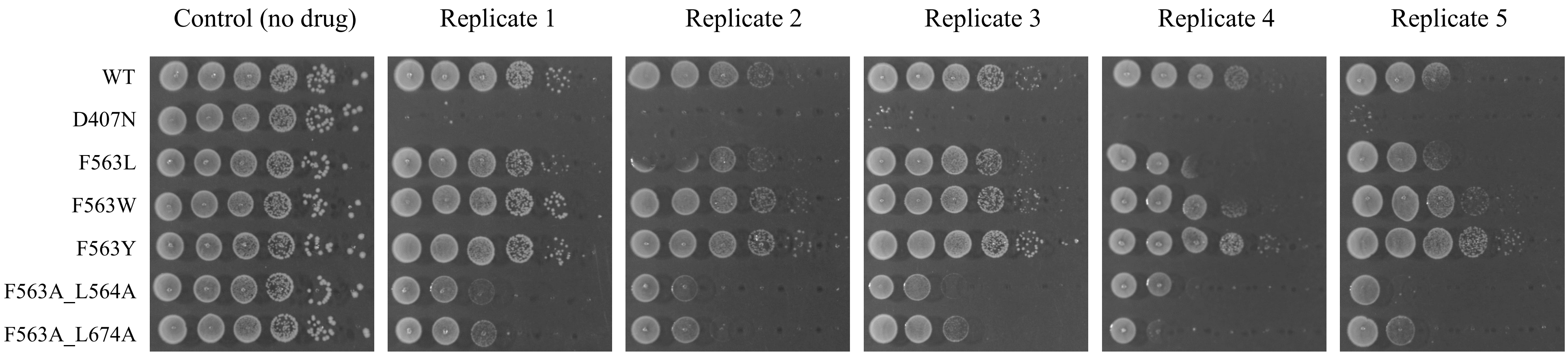


Control (no drug) – representative

Cells with $OD_{600}$ of $10^{-1}$–$10^{-6}$ were pipetted from left to right.

Fusidic acid (15 μg/ml)

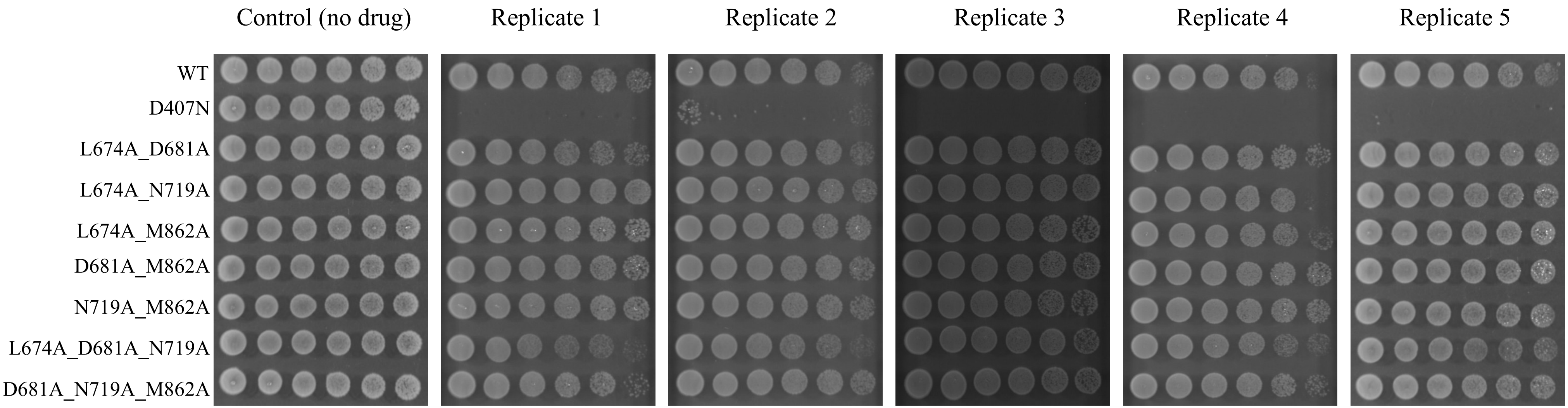


Control (no drug) – representative

Cells with $OD_{600}$ of $10^{-1}$–$10^{-6}$ were pipetted from left to right.

Fusidic acid (15 μg/ml)

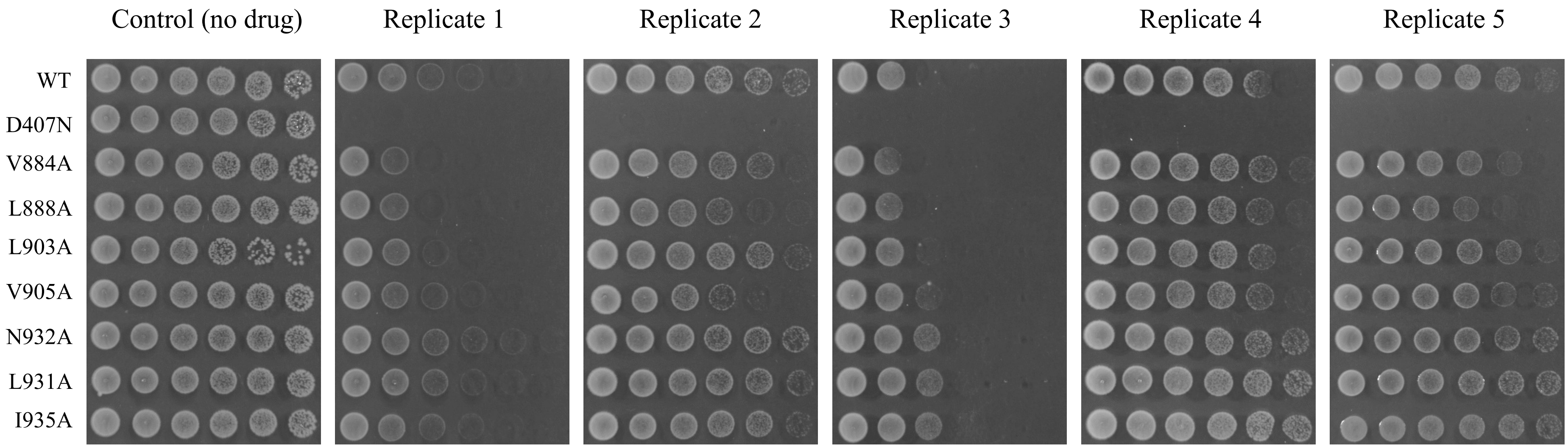


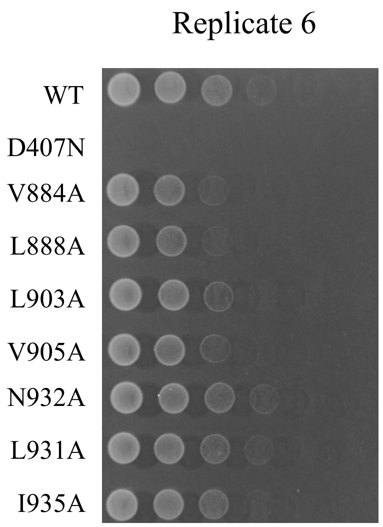


Control (no drug) – representative

Cells with $OD_{600}$ of $10^{-1}$–$10^{-6}$ were pipetted from left to right.

Fusidic acid (15 µg/ml)

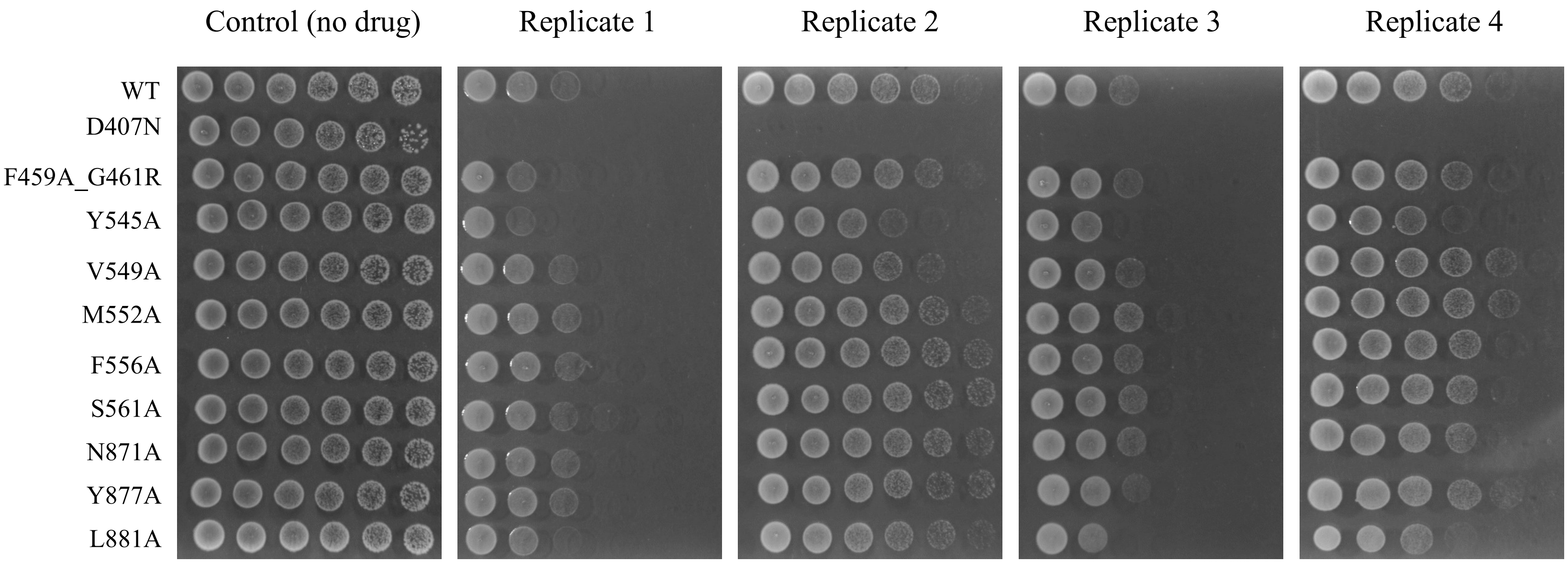


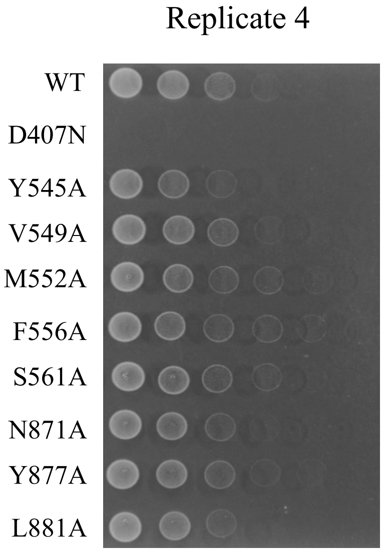


Control (no drug) – representative

Cells with $OD_{600}$ of $10^{-1}$–$10^{-6}$ were pipetted from left to right.

Fusidic acid (15 μg/ml)

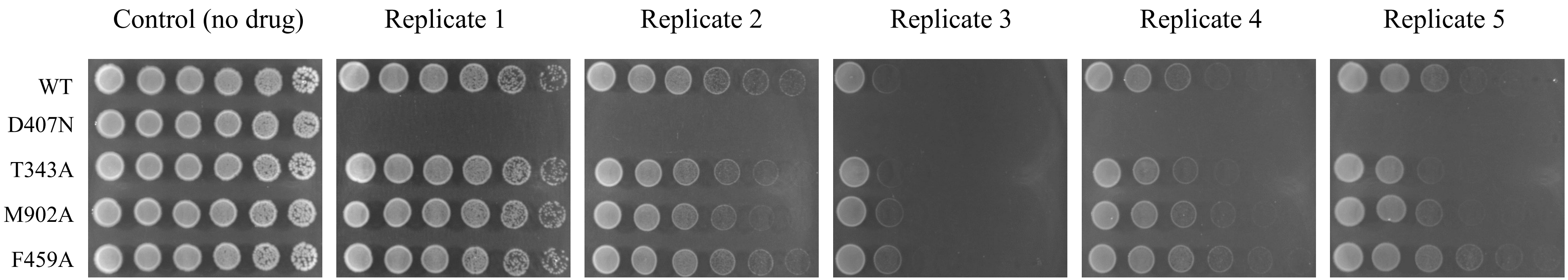


Control (no drug) – representative

Cells with $OD_{600}$ of $10^{-1}$–$10^{-6}$ were pipetted from left to right.

Fusidic acid (15 μg/ml)

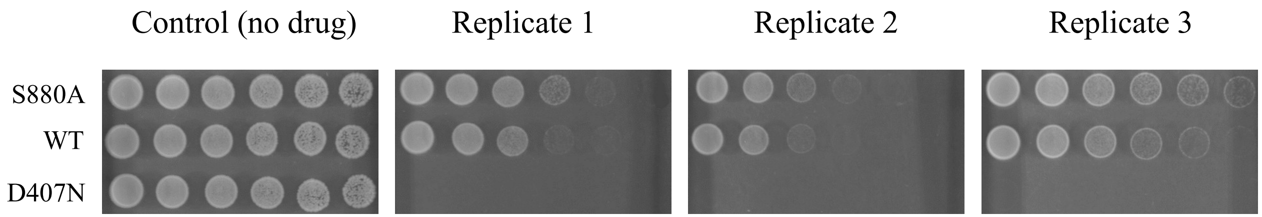


Control (no drug) – representative

Cells with $OD_{600}$ of $10^{-1}$–$10^{-6}$ were pipetted from left to right.

Fusidic acid (12–15 μg/ml)

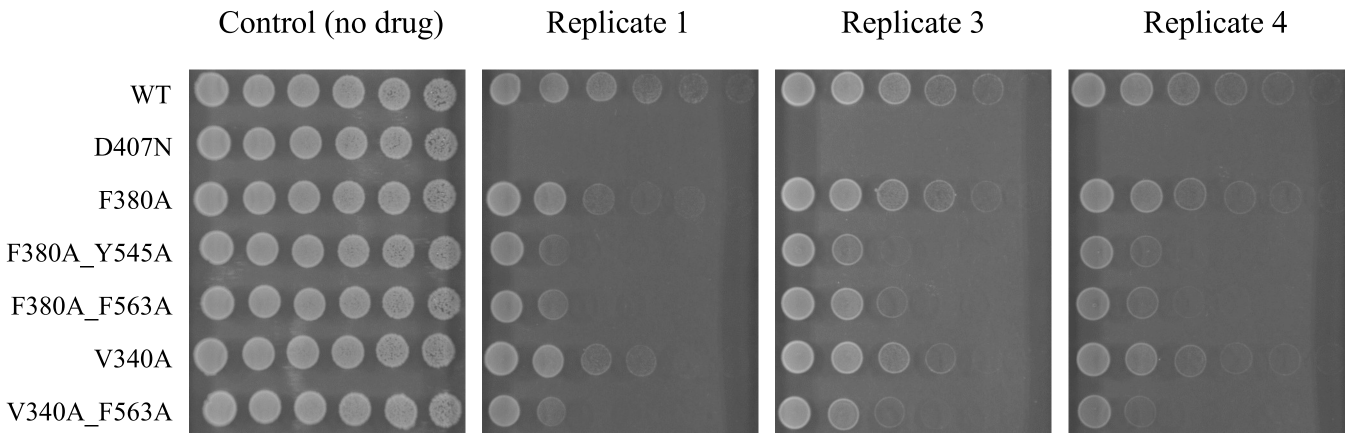


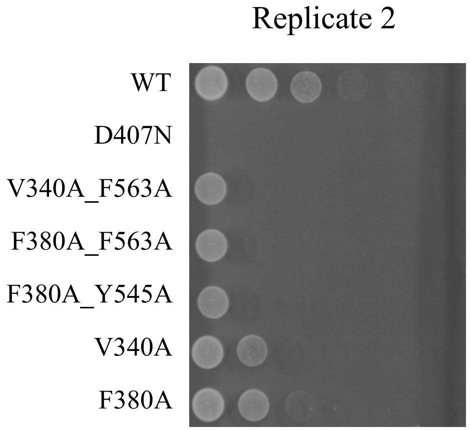


Control (no drug) – representative

Cells with $OD_{600}$ of $10^{-1}$–$10^{-6}$ were pipetted from left to right.

Fusidic acid (15 µg/ml)

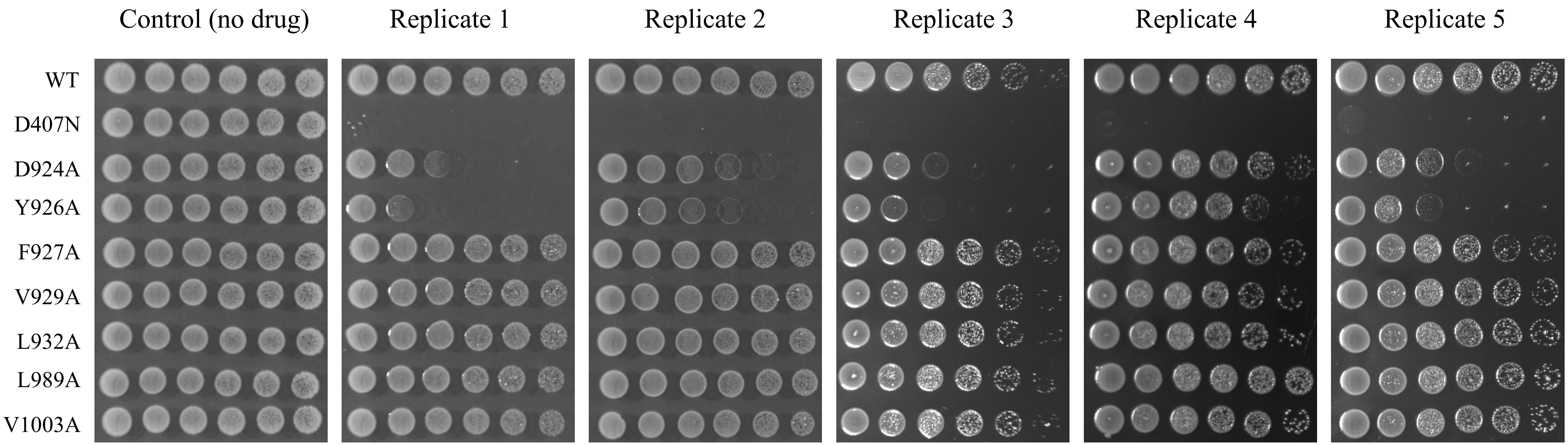


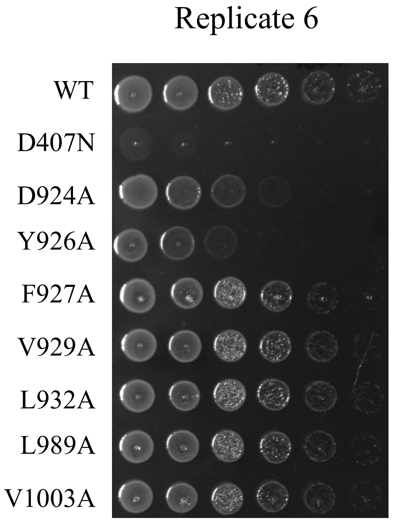


Control (no drug) – representative

Cells with $OD_{600}$ of $10^{-1}$–$10^{-6}$ were pipetted from left to right.

Fusidic acid (12.5–15 µg/ml)

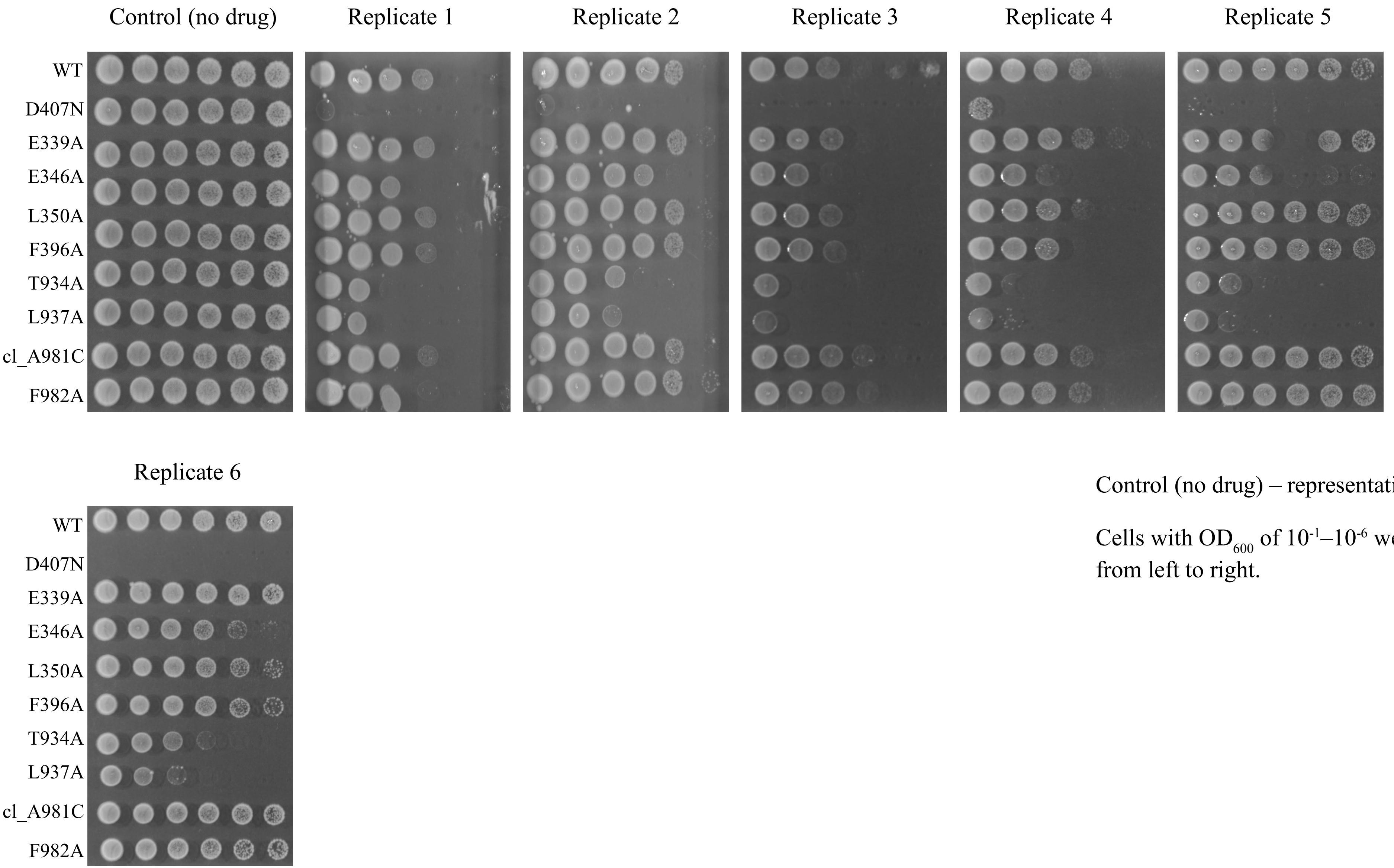


Control (no drug) – representative

Cells with $OD_{600}$ of $10^{-1}$–$10^{-6}$ were pipetted from left to right.

Fusidic acid (14–18 µg/ml)

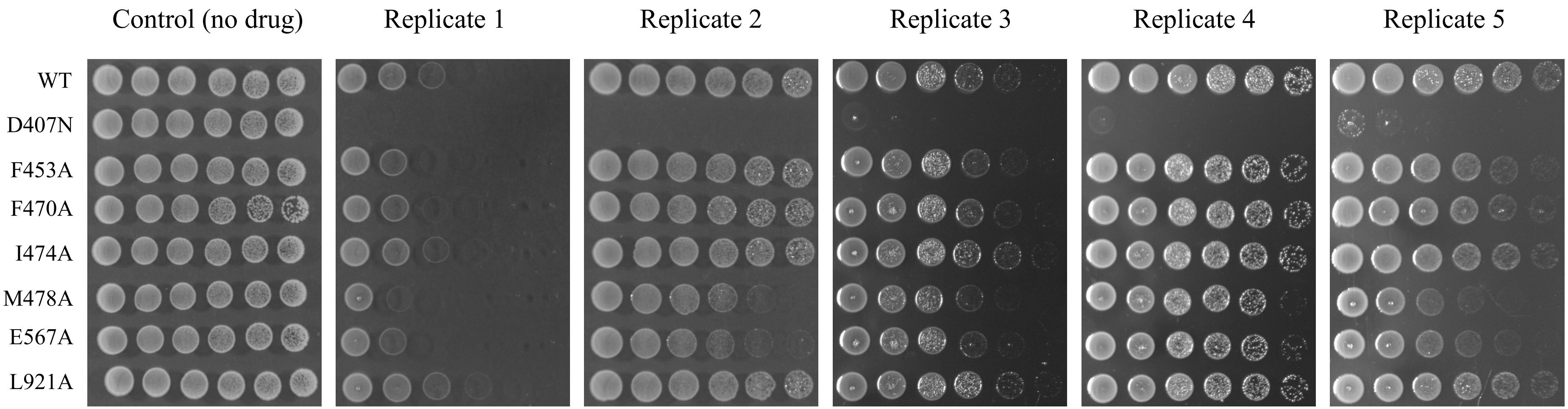


Control (no drug) – representative

Cells with $OD_{600}$ of $10^{-1}$–$10^{-6}$ were pipetted from left to right.

Fusidic acid (15–16 μg/ml)

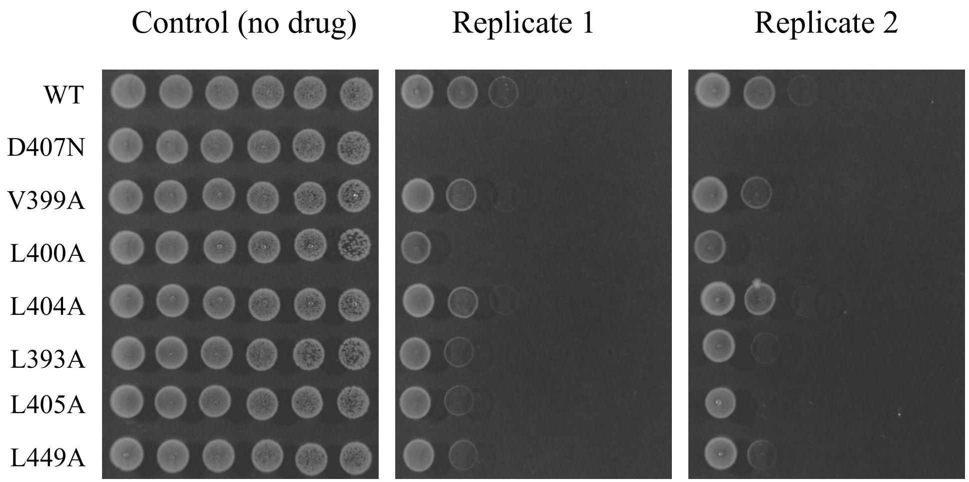


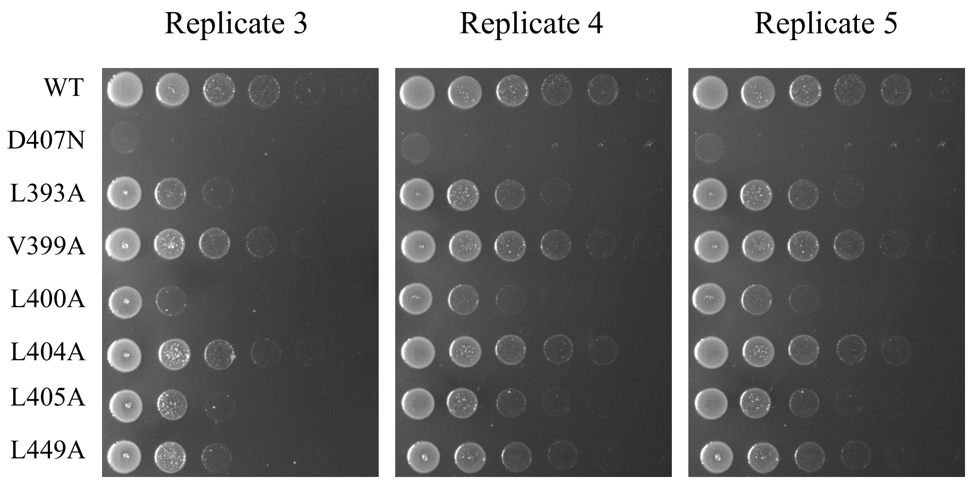


Control (no drug) – representative

Cells with $OD_{600}$ of $10^{-1}$–$10^{-6}$ were pipetted from left to right.
