# Supplementary material for: Allosteric drug transport mechanism of multidrug transporter AcrB
Source: Nat Commun. 2021 Jun 29;12:3889. doi: 10.1038/s41467-021-24151-3 (PMC8242077; doi:10.1038/s41467-021-24151-3)
Supplement: Supplementary file 8 — Source Data [file 41467_2021_24151_MOESM8_ESM.zip › Source_Data_Supplementary_Data-1_2c.zip!/Source_Data_Supplementary_Data-1_Data-2c_Linezolid.pdf]

Linezolid (15 µg/ml)

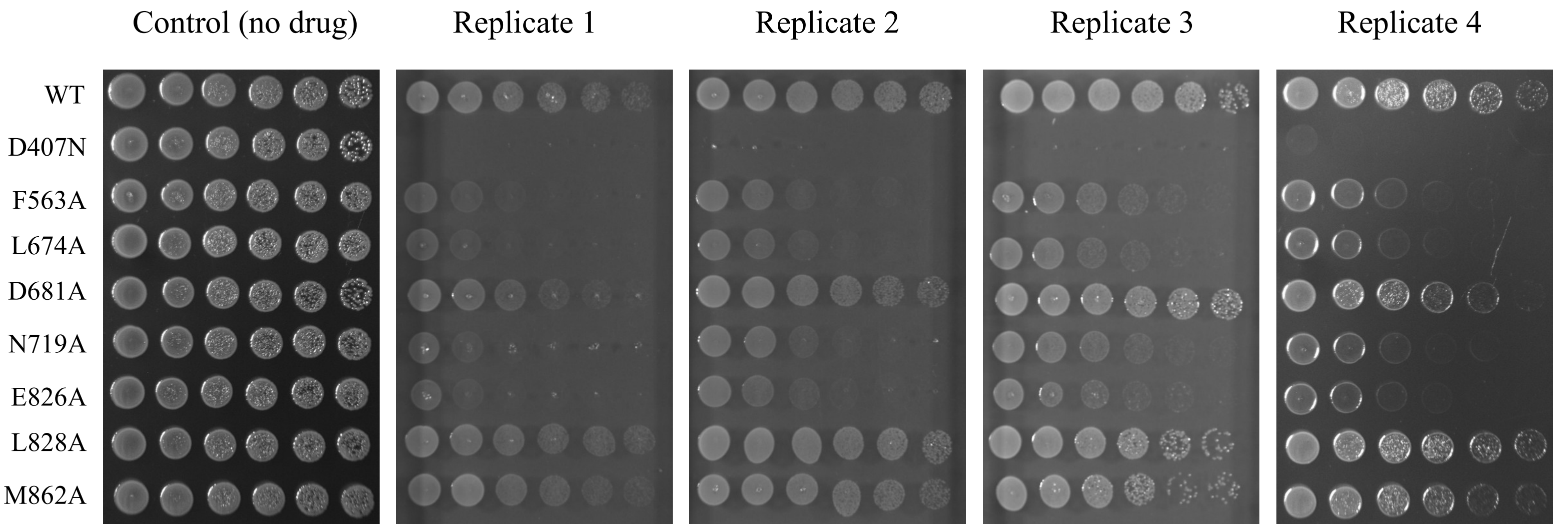

Control (no drug) – representative

Cells with OD_{600} of 10^{-1}–10^{-6} were pipetted from left to right.

Linezolid (15 μg/ml)

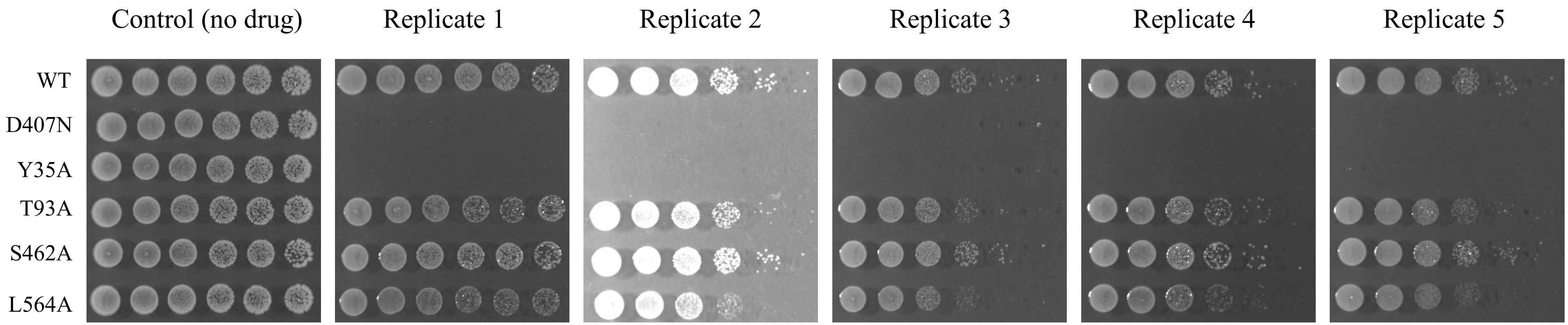

Control (no drug) – representative

Cells with OD_{600} of 10^{-1}–10^{-6} were pipetted from left to right.

Linezolid (16 µg/ml)

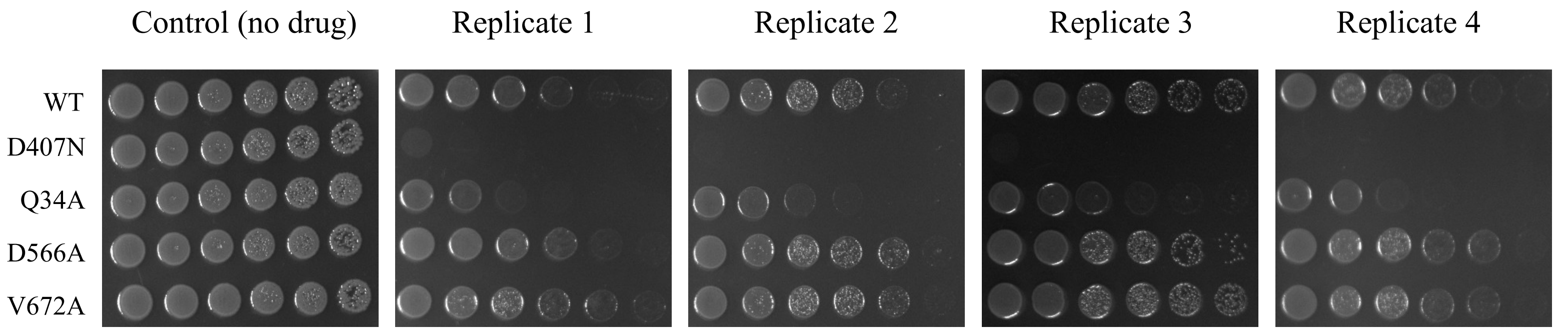

Control (no drug) – representative

Cells with OD_{600} of 10^{-1}–10^{-6} were pipetted from left to right.

Linezolid (15 µg/ml)

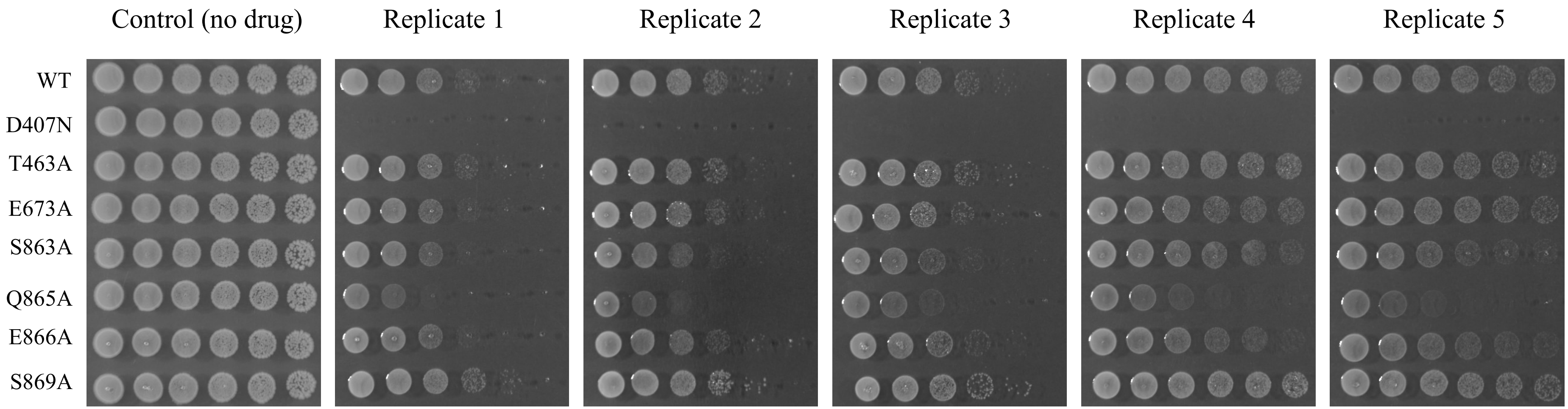

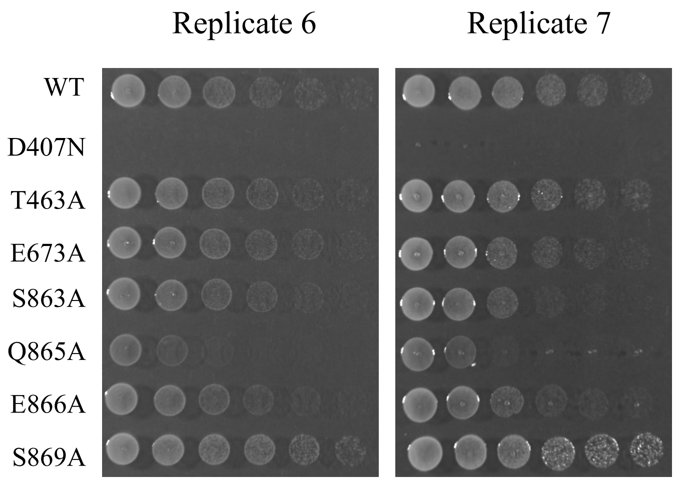

Control (no drug) – representative

Cells with OD_{600} of 10^{-1}–10^{-6} were pipetted from left to right.

Linezolid (15 μg/ml)

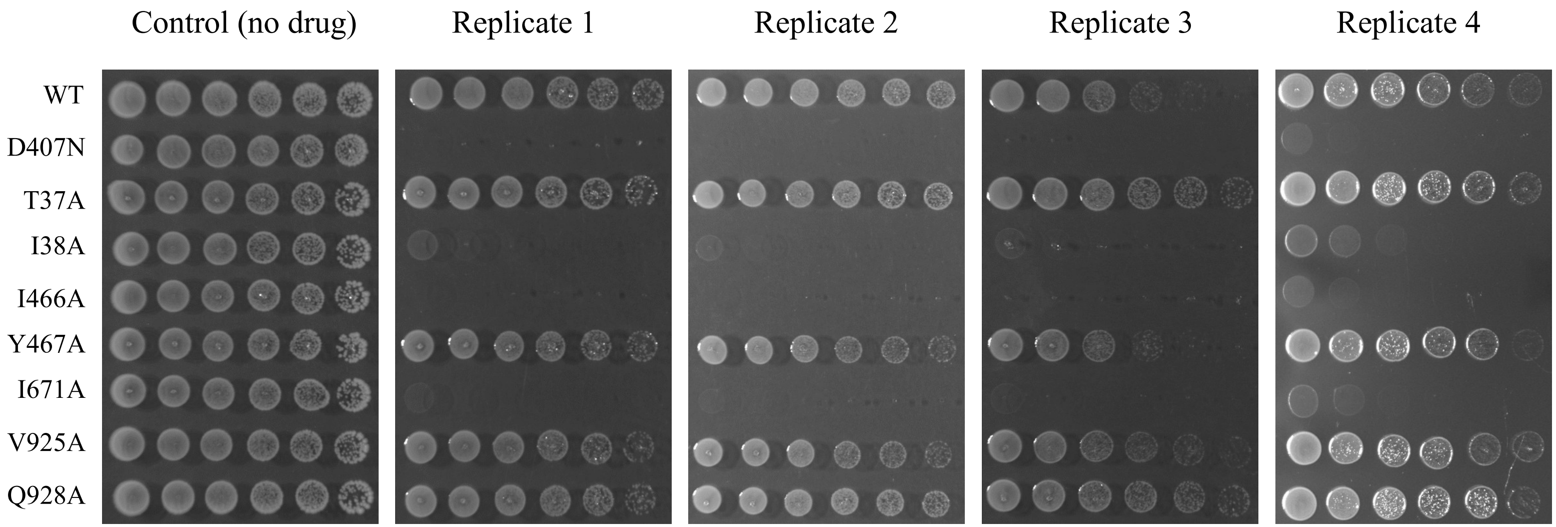

Control (no drug) – representative

Cells with OD_{600} of 10^{-1}–10^{-6} were pipetted from left to right.

Linezolid (15 µg/ml)

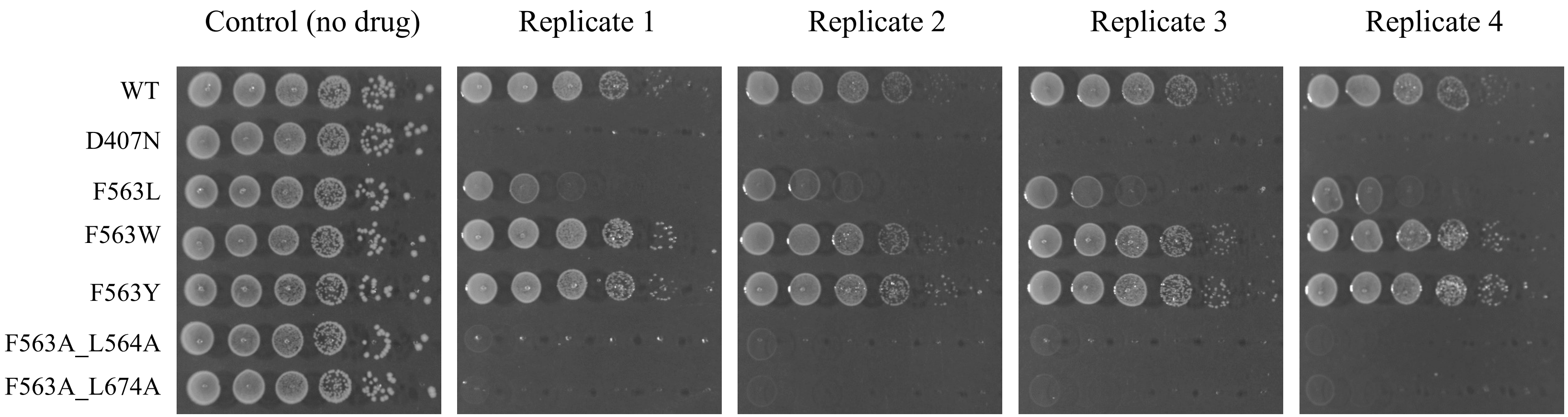

Control (no drug) – representative

Cells with OD_{600} of 10^{-1}–10^{-6} were pipetted from left to right.

Linezolid (15 μg/ml)

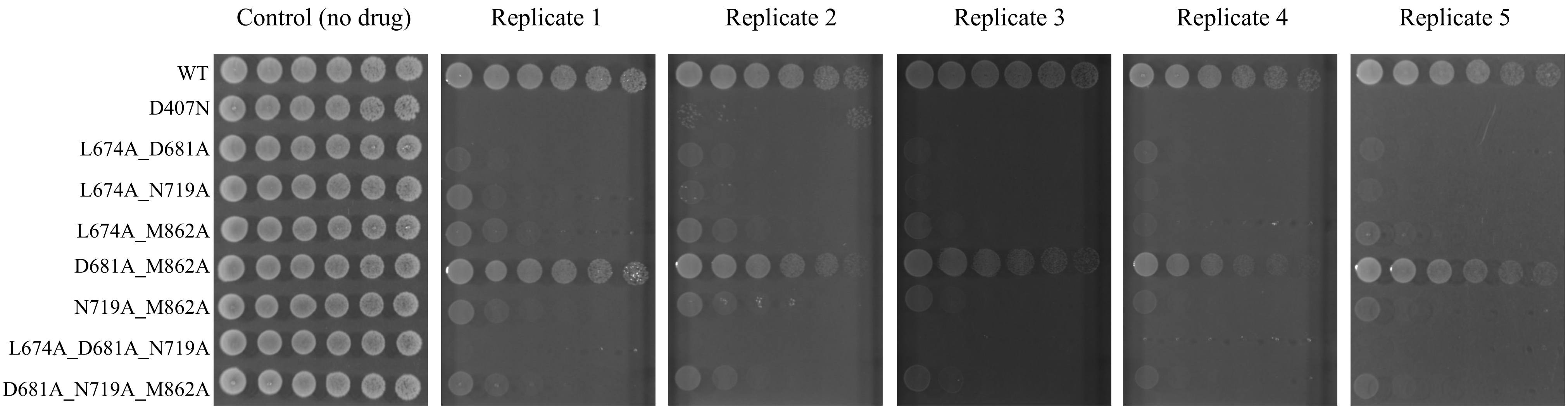

Control (no drug) – representative

Cells with OD_{600} of 10^{-1}–10^{-6} were pipetted from left to right.

Linezolid (15 μg/ml)

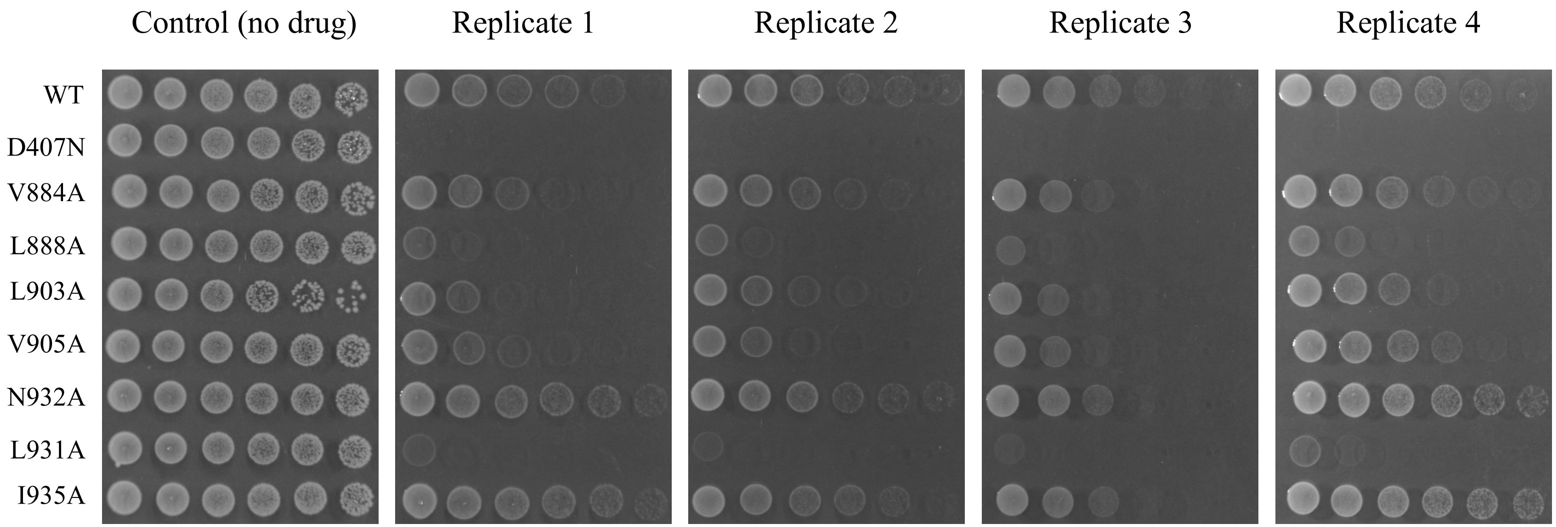

Control (no drug) – representative

Cells with OD_{600} of 10^{-1}–10^{-6} were pipetted from left to right.

Linezolid (15 µg/ml)

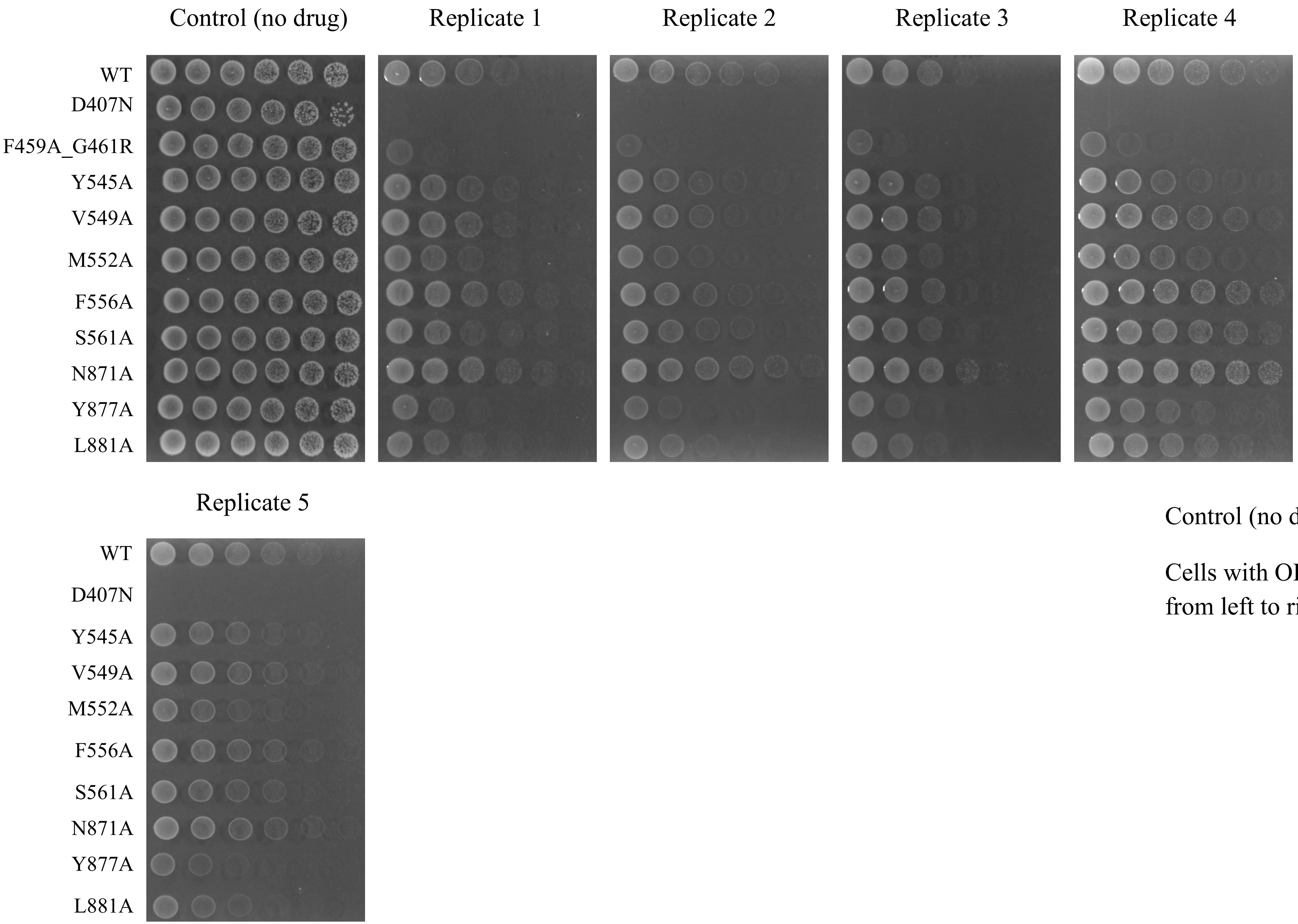

Control (no drug) – representative

Cells with OD_{600} of 10^{-1}–10^{-6} were pipetted from left to right.

Linezolid (15 μg/ml)

Control (no drug) | Replicate 1 | Replicate 2 | Replicate 3 | Replicate 4 | Replicate 5

WT
D407N
T343A
M902A
F459A

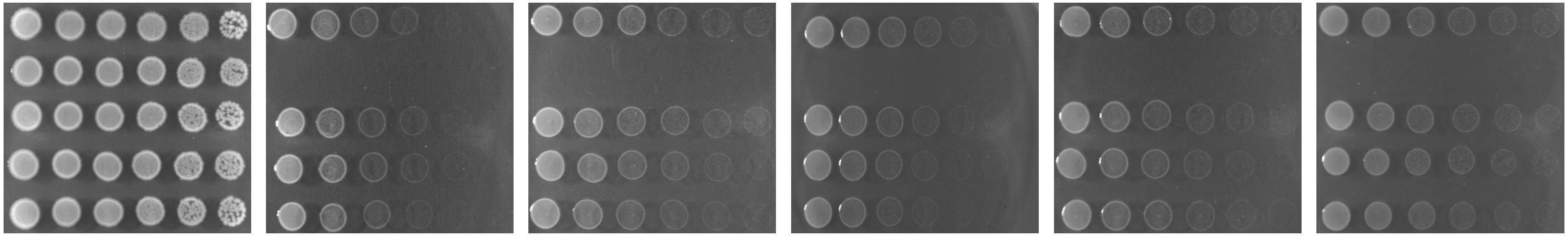

Replicate 6

WT
D407N
T343A
M902A
F459A

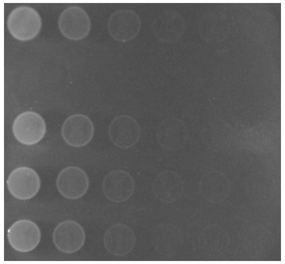

Control (no drug) – representative

Cells with OD_{600} of 10^{-1}–10^{-6} were pipetted from left to right.

Linezolid (15 µg/ml)

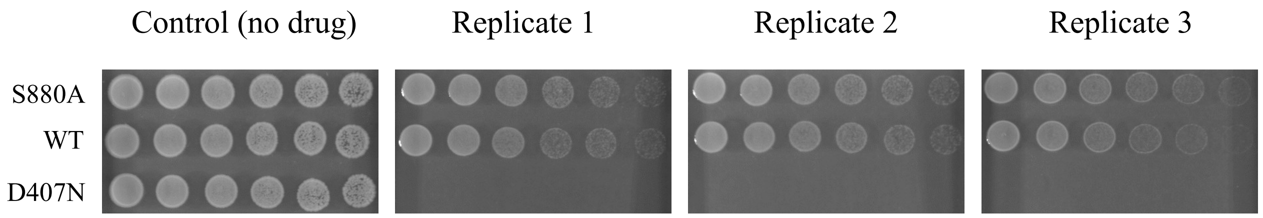

Control (no drug) – representative

Cells with OD_{600} of 10^{-1}–10^{-6} were pipetted from left to right.

Linezolid (15–16 μg/ml)

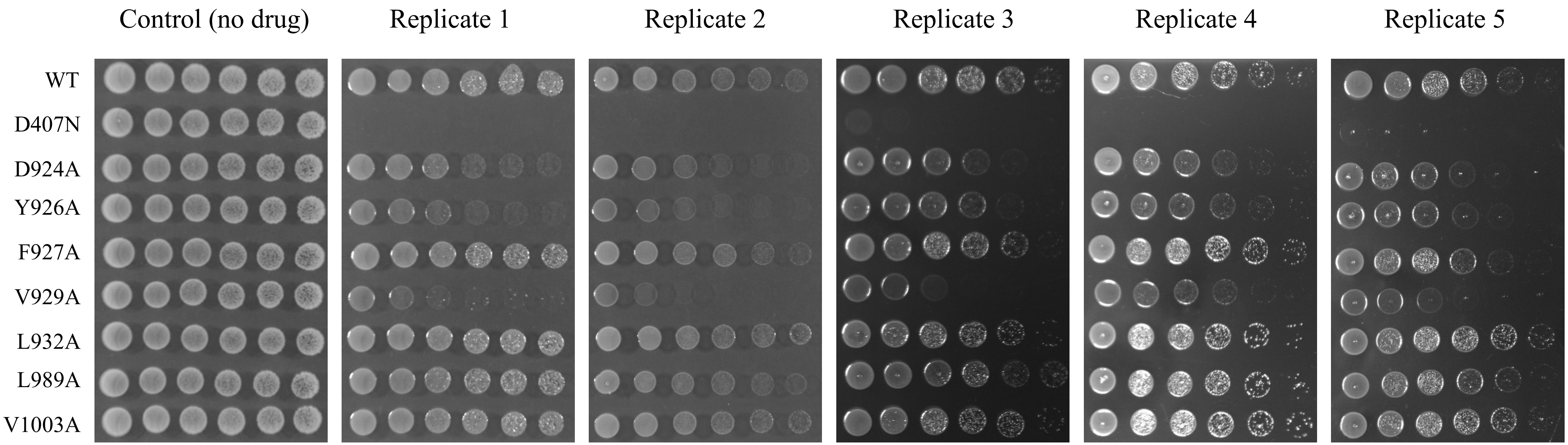

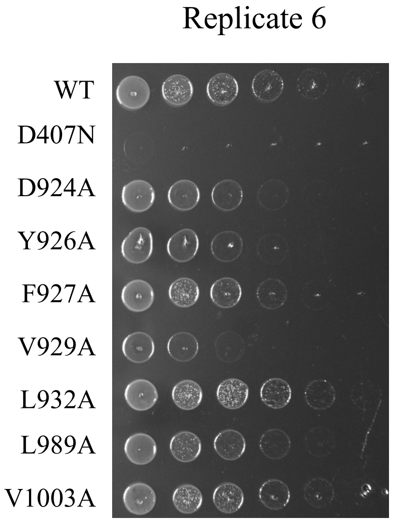

Control (no drug) – representative

Cells with OD_{600} of 10^{-1}–10^{-6} were pipetted from left to right.

Linezolid (12–16 μg/ml)

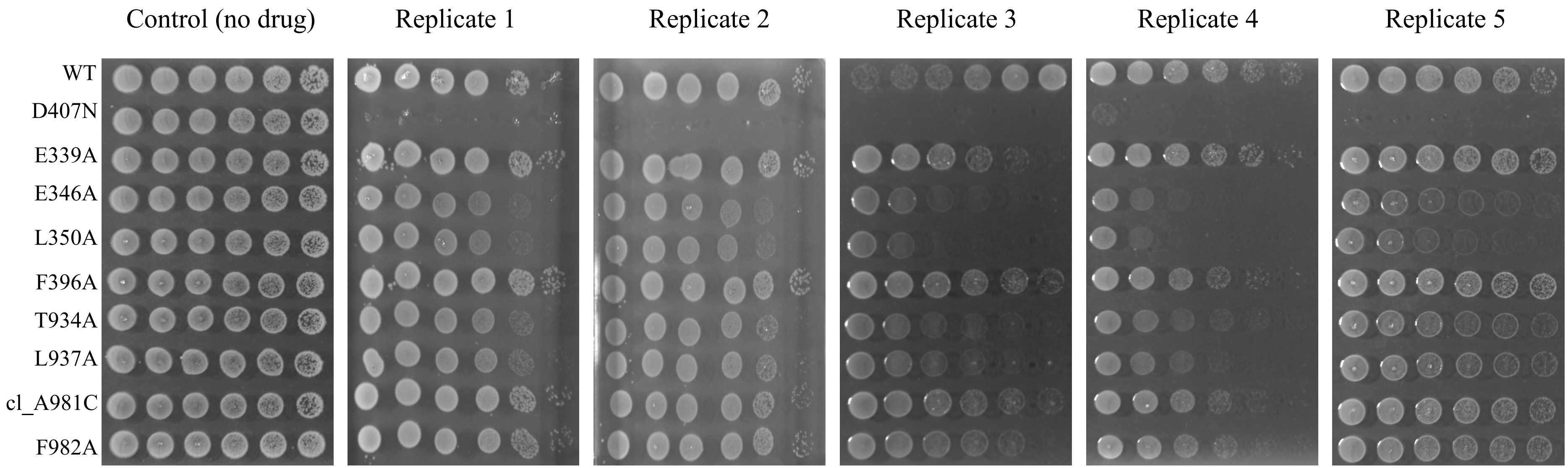

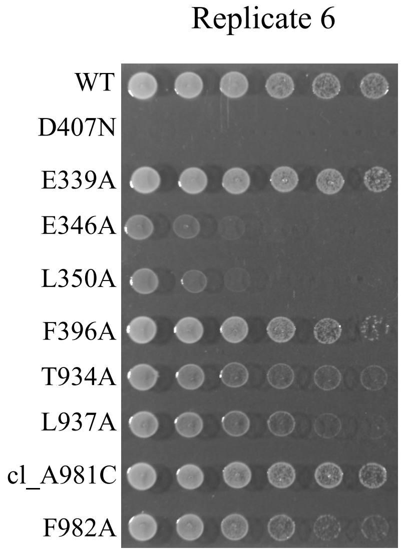

Control (no drug) – representative

Cells with OD_{600} of 10^{-1}–10^{-6} were pipetted from left to right.

Replicate 3: only WT where cells with OD_{600} of 10^{-1}–10^{-6} were pipetted from right to left.

Linezolid (16 µg/ml)

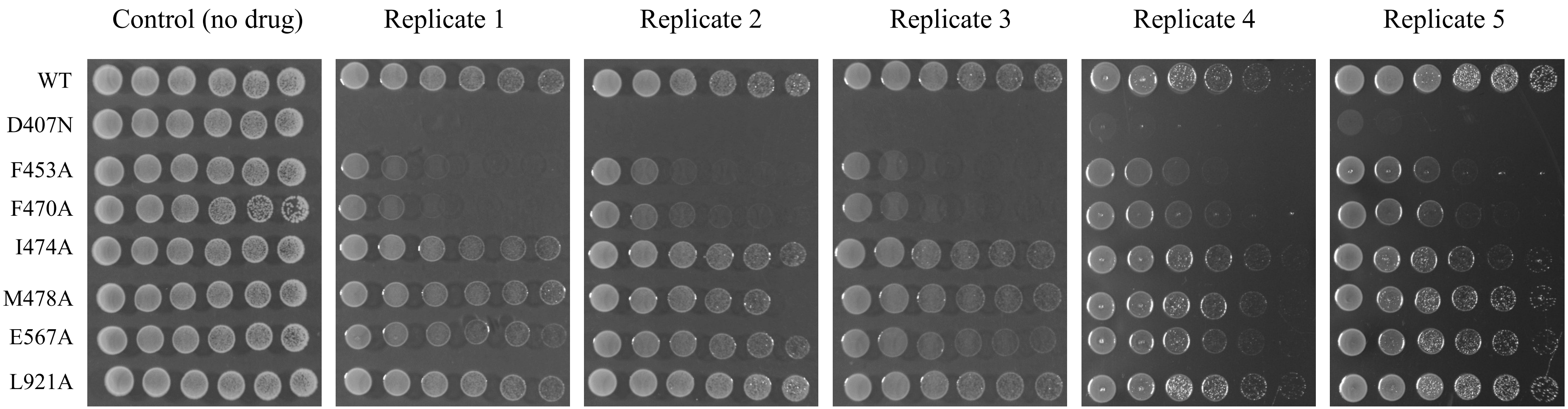

Control (no drug) – representative

Cells with OD_{600} of 10^{-1}–10^{-6} were pipetted from left to right.

Linezolid (15 µg/ml)

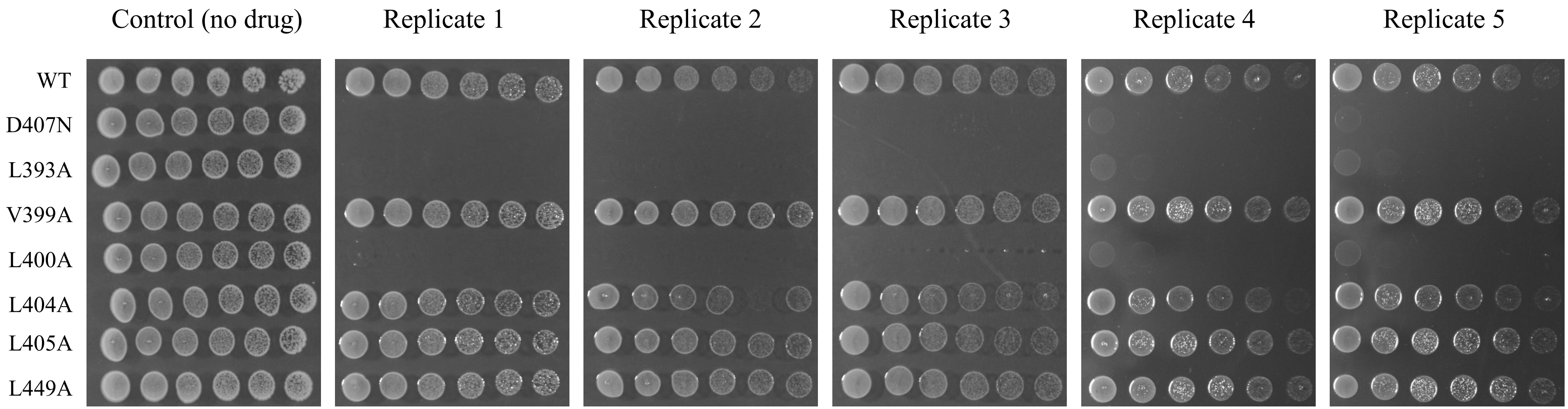

Control (no drug) – representative

Cells with OD_{600} of 10^{-1}–10^{-6} were pipetted from left to right.
