# Supplementary material for: Allosteric drug transport mechanism of multidrug transporter AcrB
Source: Nat Commun. 2021 Jun 29;12:3889. doi: 10.1038/s41467-021-24151-3 (PMC8242077; doi:10.1038/s41467-021-24151-3)
Supplement: Supplementary file 8 — Source Data [file 41467_2021_24151_MOESM8_ESM.zip › Source_Data_Supplementary_Data-1_2c.zip!/Source_Data_Supplementary_Data-1_Data-2c_Western_blot.pdf]

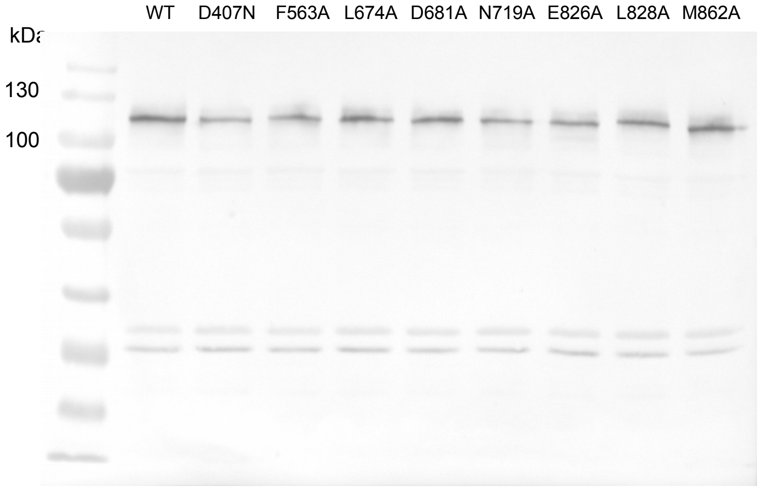

WT
D407N
F563A
L674A
D681A
N719A
E826A
L828A
M862A
kDa
130
100

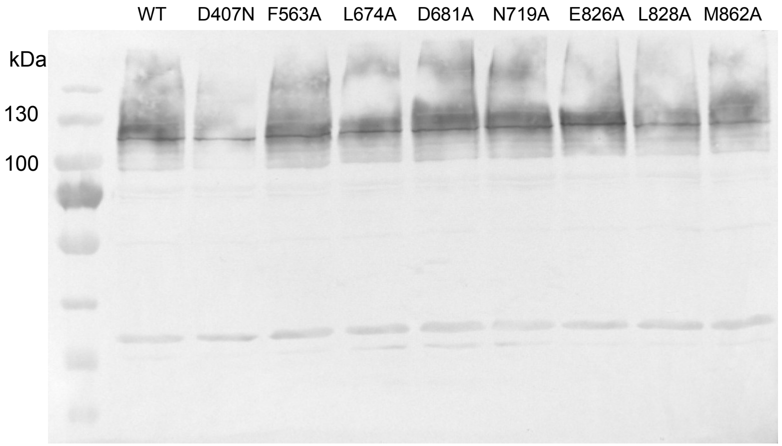

WT
D407N
F563A
L674A
D681A
N719A
E826A
L828A
M862A
kDa
130
100

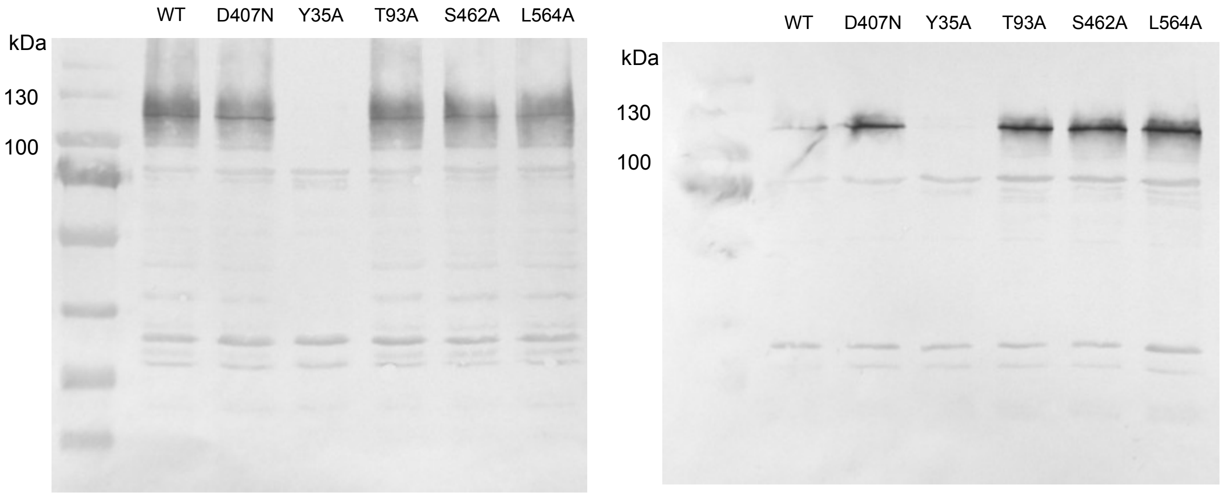

WT
D407N
Y35A
T93A
S462A
L564A
kDa
130
100
WT
D407N
Y35A
T93A
S462A
L564A
kDa
130
100

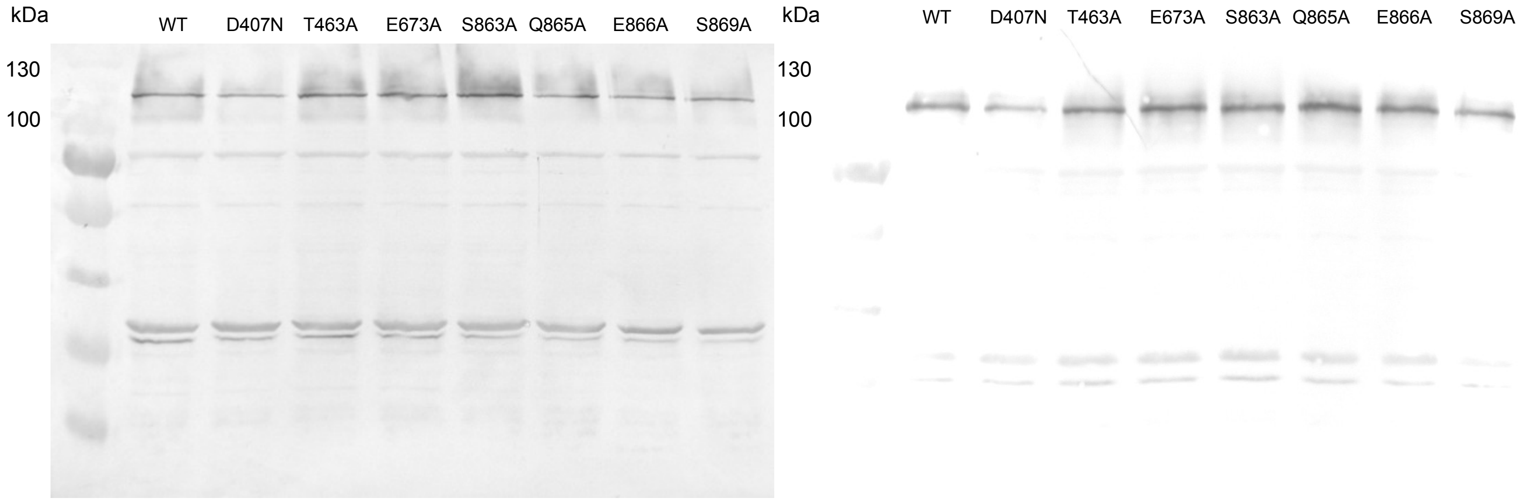
kDa
WT
D407N
T463A
E673A
S863A
Q865A
E866A
S869A
130
100
kDa
WT
D407N
T463A
E673A
S863A
Q865A
E866A
S869A
130
100

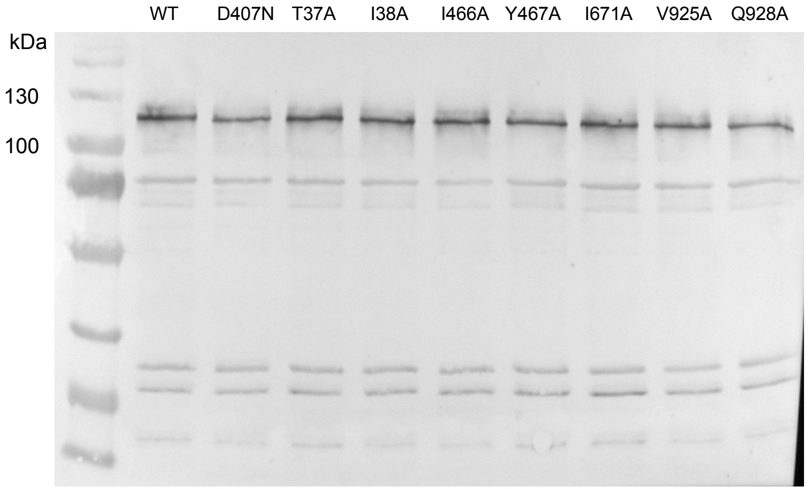
WT
D407N
T37A
I38A
I466A
Y467A
I671A
V925A
Q928A
kDa
130
100

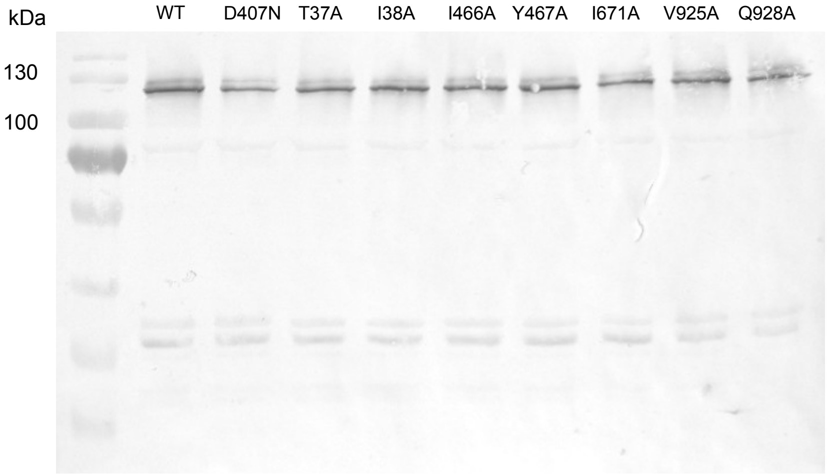
kDa
WT
D407N
T37A
I38A
I466A
Y467A
I671A
V925A
Q928A
130
100

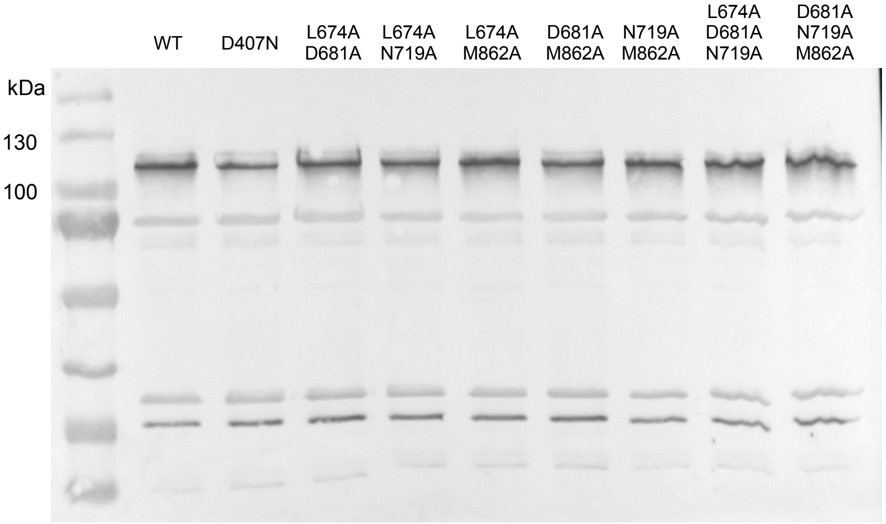
WT
D407N
L674A D681A
L674A N719A
L674A M862A
D681A M862A
N719A M862A
L674A D681A N719A
D681A N719A M862A
kDa
130
100

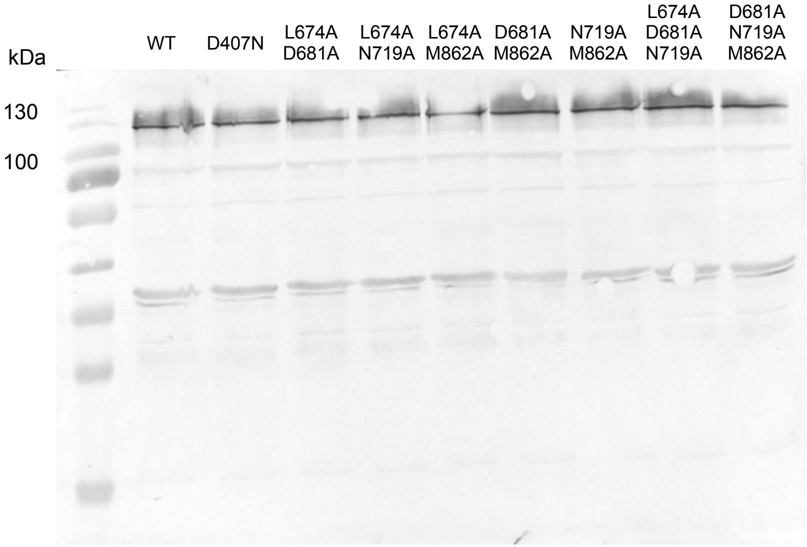
WT
D407N
L674A D681A
L674A N719A
L674A M862A
D681A M862A
N719A M862A
L674A D681A N719A
D681A N719A M862A
kDa
130
100

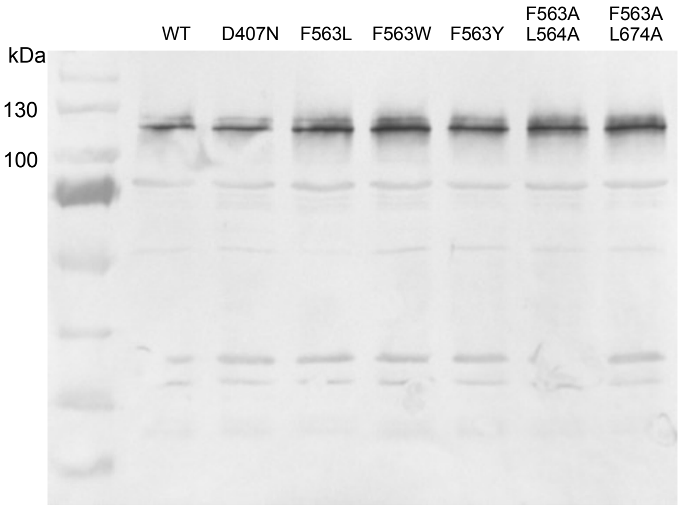

WT
D407N
F563L
F563W
F563Y
F563A L564A
F563A L674A
kDa
130
100

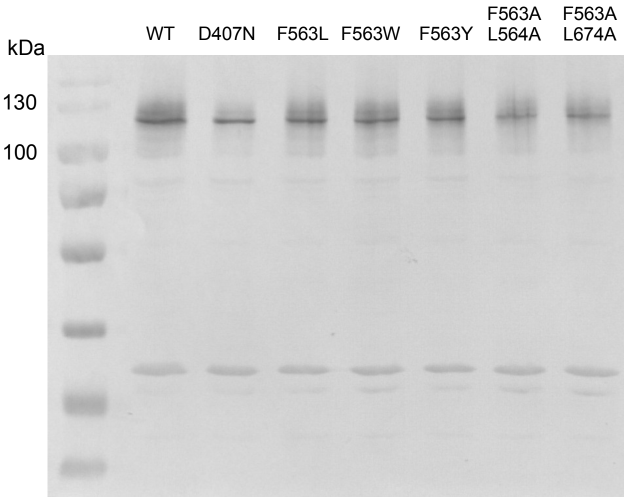

WT
D407N
F563L
F563W
F563Y
F563A L564A
F563A L674A
kDa
130
100

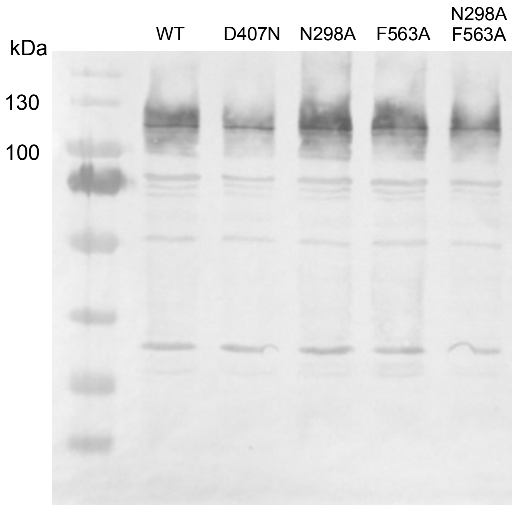
WT
D407N
N298A
F563A
N298A
F563A
kDa
130
100

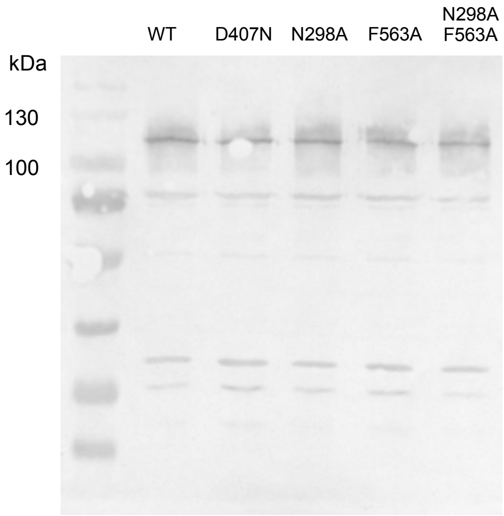
WT
D407N
N298A
F563A
N298A
F563A
kDa
130
100

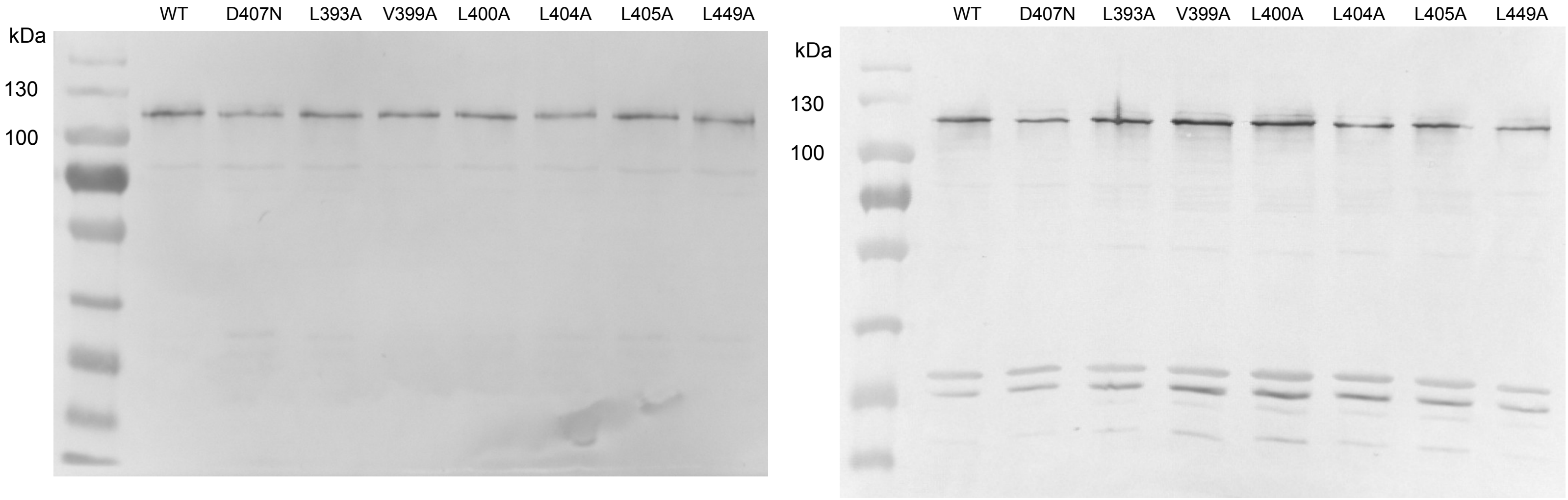
kDa
130
100
WT
D407N
L393A
V399A
L400A
L404A
L405A
L449A
kDa
130
100
WT
D407N
L393A
V399A
L400A
L404A
L405A
L449A

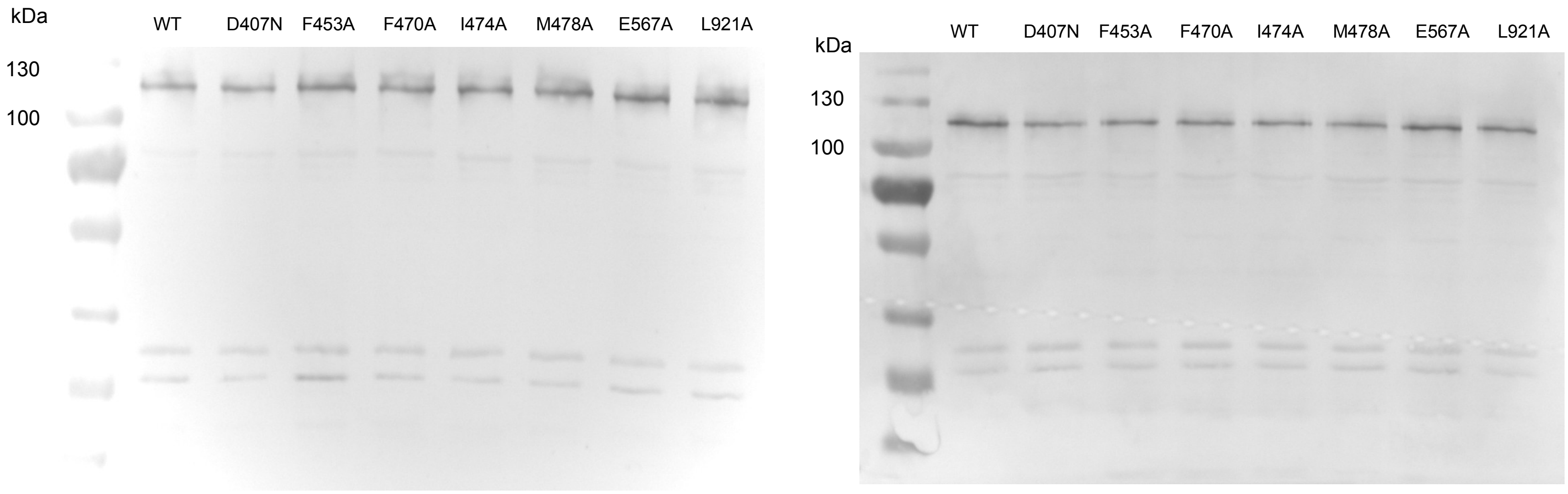

kDa
WT
D407N
F453A
F470A
I474A
M478A
E567A
L921A
130
100
kDa
WT
D407N
F453A
F470A
I474A
M478A
E567A
L921A
130
100

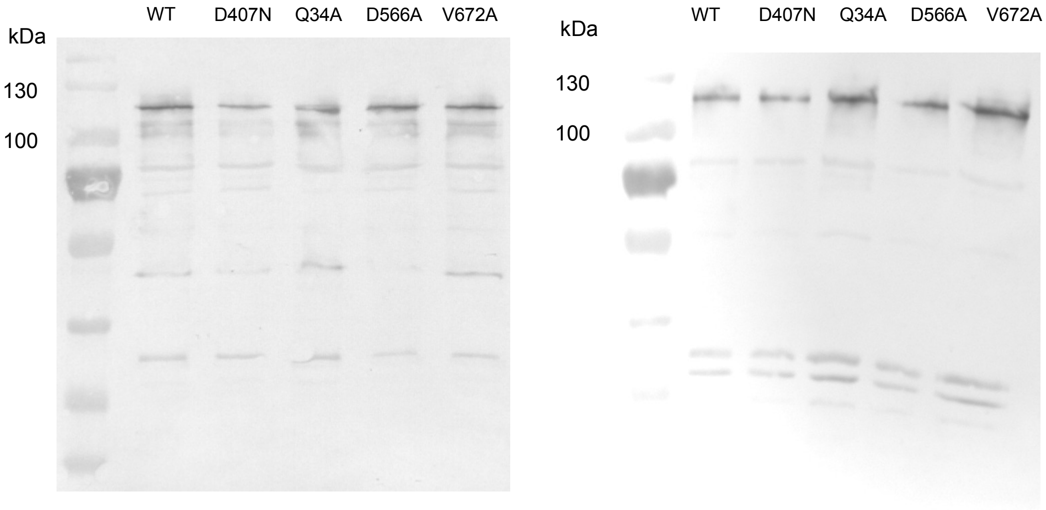
kDa
130
100
WT
D407N
Q34A
D566A
V672A
kDa
130
100
WT
D407N
Q34A
D566A
V672A

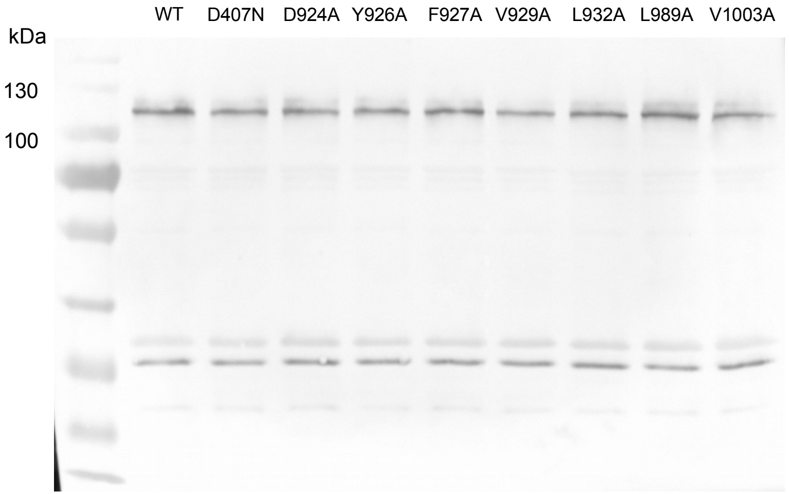
WT
D407N
D924A
Y926A
F927A
V929A
L932A
L989A
V1003A
kDa
130
100

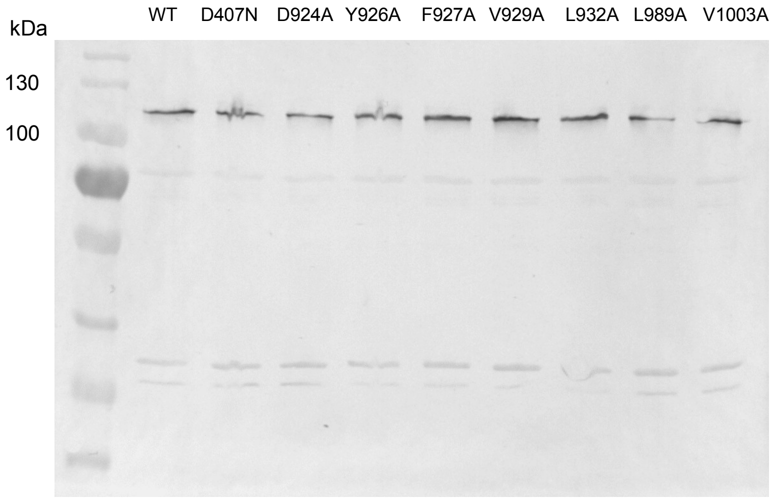
WT
D407N
D924A
Y926A
F927A
V929A
L932A
L989A
V1003A
kDa
130
100

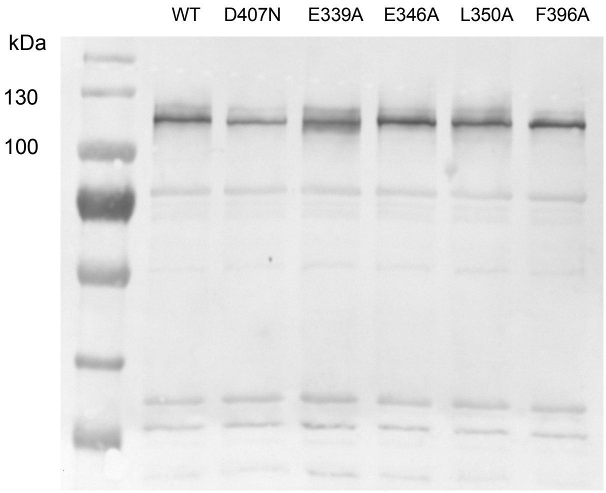
WT
D407N
E339A
E346A
L350A
F396A
kDa
130
100

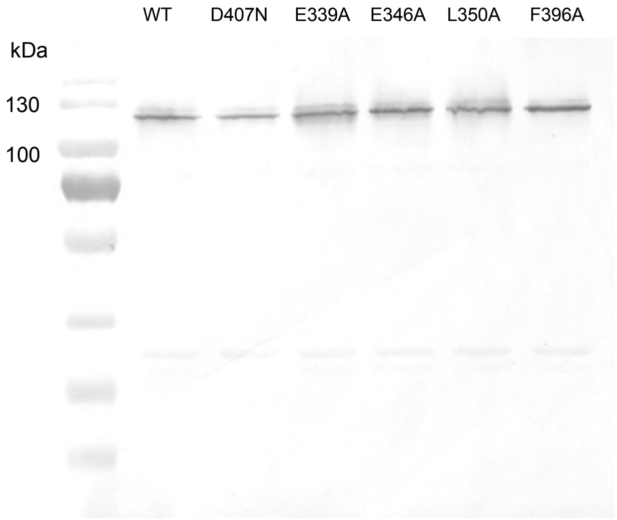
WT
D407N
E339A
E346A
L350A
F396A
kDa
130
100

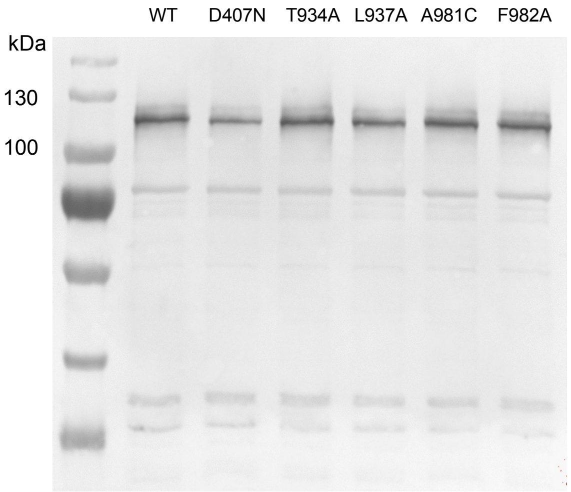
WT
D407N
T934A
L937A
A981C
F982A
kDa
130
100

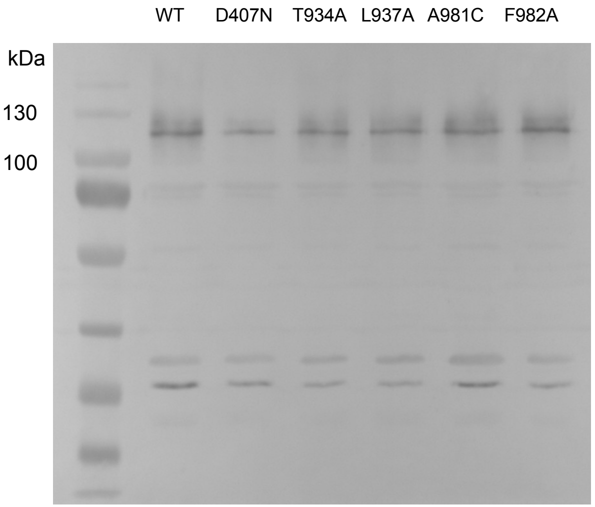
WT
D407N
T934A
L937A
A981C
F982A
kDa
130
100

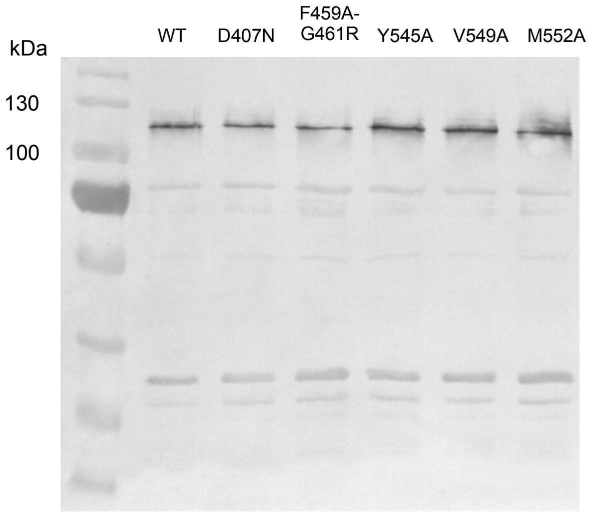
kDa
130
100
WT
D407N
F459A-
G461R
Y545A
V549A
M552A

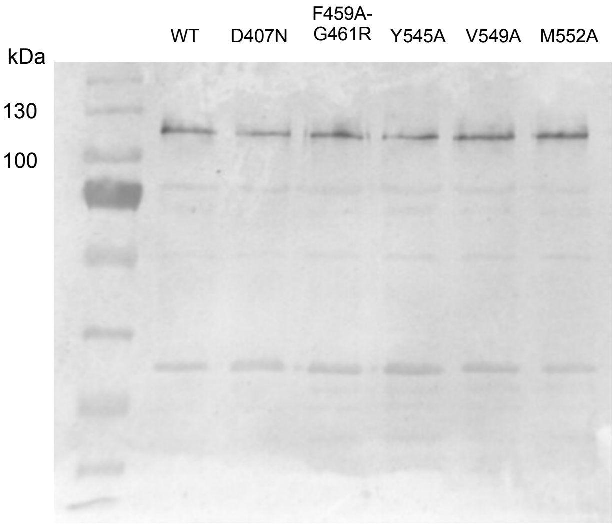
kDa
130
100
WT
D407N
F459A-
G461R
Y545A
V549A
M552A

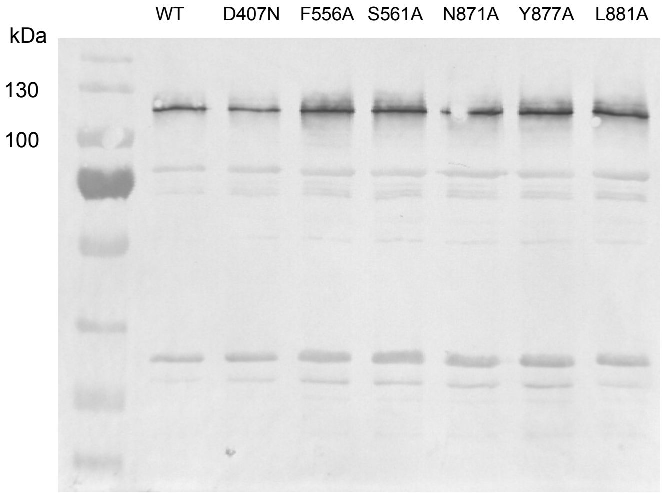
WT
D407N
F556A
S561A
N871A
Y877A
L881A
kDa
130
100
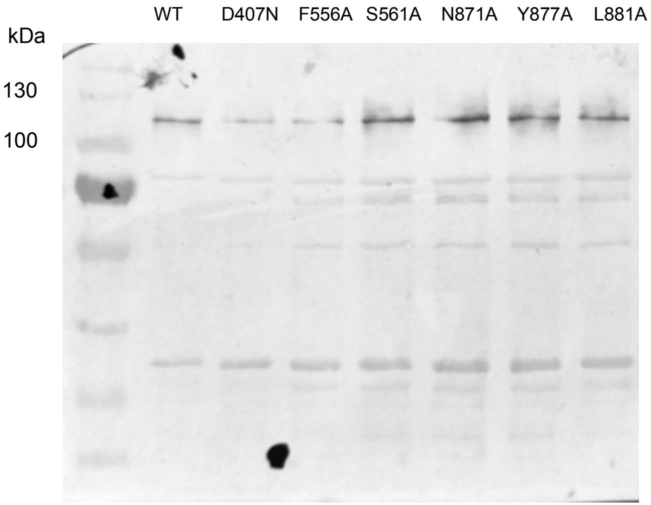
WT
D407N
F556A
S561A
N871A
Y877A
L881A
kDa
130
100

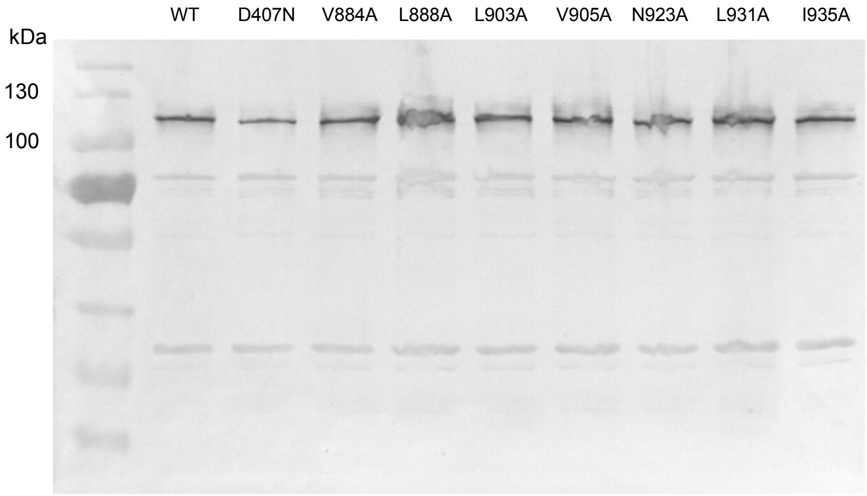
WT
D407N
V884A
L888A
L903A
V905A
N923A
L931A
I935A
kDa
130
100

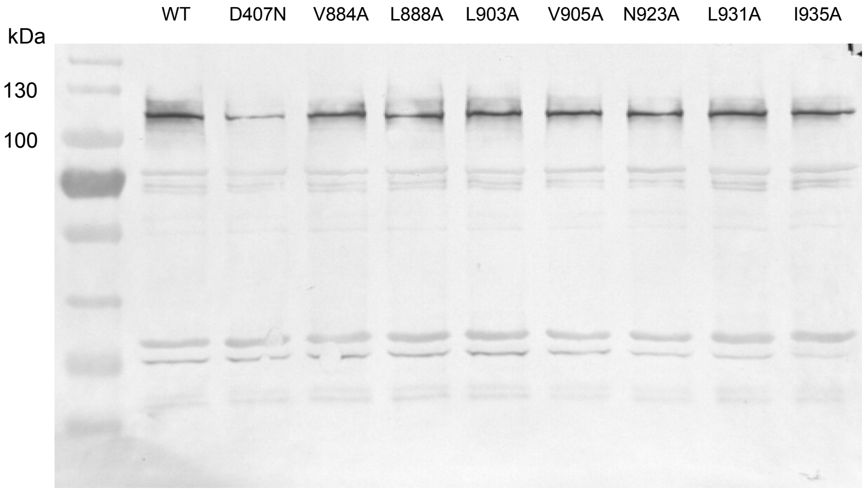
WT
D407N
V884A
L888A
L903A
V905A
N923A
L931A
I935A
kDa
130
100

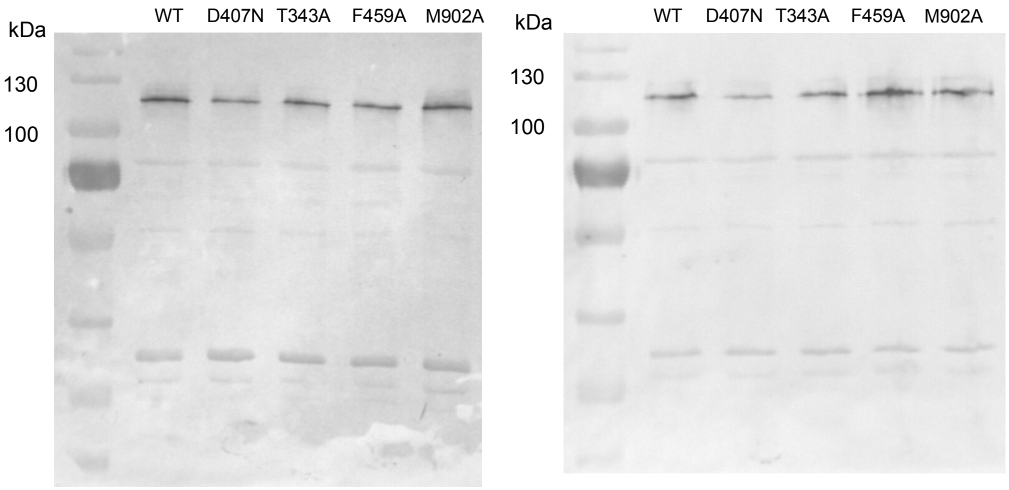
kDa
WT
D407N
T343A
F459A
M902A
130
100
kDa
WT
D407N
T343A
F459A
M902A
130
100

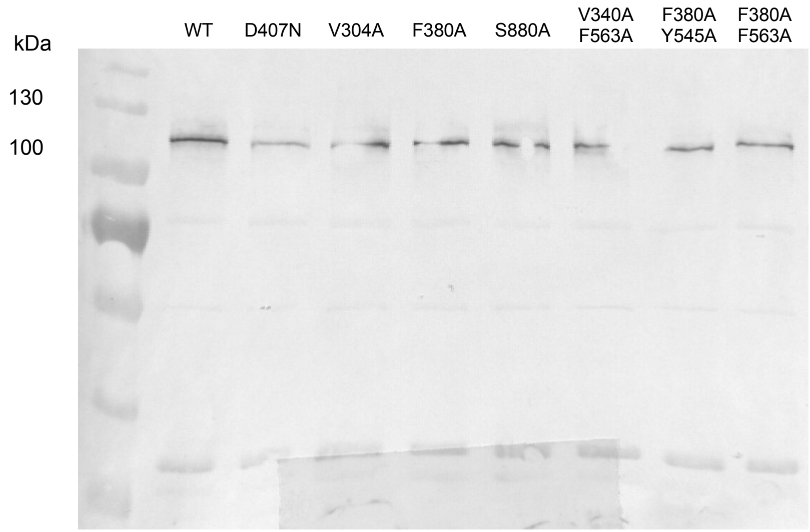
kDa
130
100
WT
D407N
V304A
F380A
S880A
V340A F563A
F380A Y545A
F380A F563A

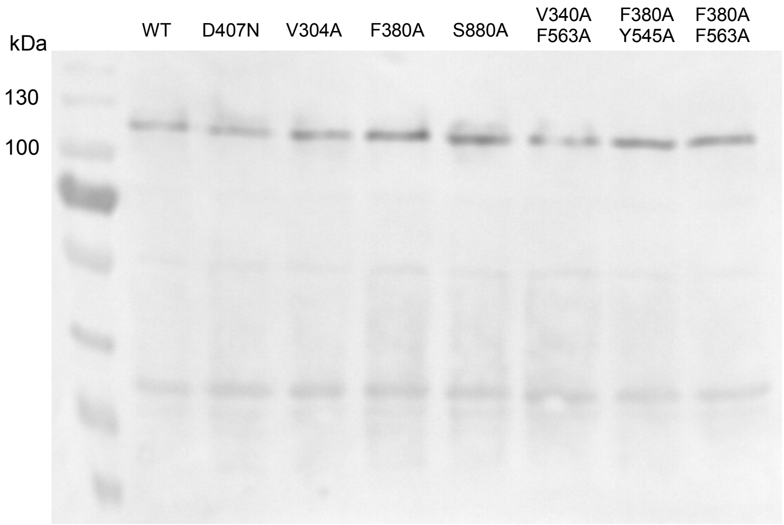
kDa
130
100
WT
D407N
V304A
F380A
S880A
V340A F563A
F380A Y545A
F380A F563A
