# Supplementary material for: Allosteric drug transport mechanism of multidrug transporter AcrB
Source: Nat Commun. 2021 Jun 29;12:3889. doi: 10.1038/s41467-021-24151-3 (PMC8242077; doi:10.1038/s41467-021-24151-3)
Supplement: Supplementary file 2 — Peer Review File [file 41467_2021_24151_MOESM2_ESM.pdf]

Editorial Note: This manuscript has been previously reviewed at another journal that is not operating a transparent peer review scheme. The manuscript was considered suitable for publication without further review at *Nature Communications.*
